# Weak Interactions and Conformational Changes in Core-Protonated A_2_- and A_x_-Type Porphyrin Dications

**DOI:** 10.3390/molecules25143195

**Published:** 2020-07-13

**Authors:** Christopher J. Kingsbury, Keith J. Flanagan, Hans-Georg Eckhardt, Marc Kielmann, Mathias O. Senge

**Affiliations:** School of Chemistry, Trinity Biomedical Sciences Institute, Trinity College Dublin, The University of Dublin, Pearse St, 152-160 Dublin 2, Ireland; ckingsbu@tcd.ie (C.J.K.); kflanaga@tcd.ie (K.J.F.); hans.eckhardt@ucd.ie (H.-G.E.); kielmanm@tcd.ie (M.K.)

**Keywords:** porphyrins, conformational analysis, weak interactions, crystallography, nonplanar macrocycles

## Abstract

Individual chemical motifs are known to introduce structural distortions to the porphyrin macrocycle, be it in the core or at the periphery of the macrocycle. The interplay when introducing two or more of these known structural motifs has been scarcely explored and is not necessarily simply additive; these structural distortions have a chance to compound or negate to introduce new structural types. To this end, a series of compounds with complementary peripheral (5,15-disubstitution) and core (acidification) substitution patterns were investigated. The single-crystal X-ray structures of 18 5,15-diphenylporphyrin, 5,15-diphenylporphyrindi-ium diacid, and related compounds are reported, including the first example of a 5,15-dialkylporphyrindi-ium. Normal-coordinate structural decomposition (NSD) analysis is used for a detailed analysis of the conformation of the porphyrin subunit within the crystal structures. An elongation of porphyrin macrocycles along the C_5_,C_15_- axis (*B*_2*g*_ symmetry) is observed in all of the free base porphyrins and porphyrin dications; distance across the core is around 0.3 Å in the free base and diacid compounds, and more than doubled in 5,15-dipentylporphyrin and 5,15-dipentylporphyrindi-ium diacid. While the free base porphyrins are largely planar, a large out-of-plane distortion can be observed in 5,15-diphenylporphyrin diacids, with the expected “projective saddle” shape characteristic for such systems. The combination of these two distortions (*B*_2*u*_ and *B*_2*g*_) from regular porphyrin structure results in a macrocycle best characterized in the chiral point-group *D*_2_. A rare structural type of a *cis*-hydrogen bond chelate is observed for 5,15-dipentylporphyrindi-ium diacid, which adopts an achiral *C*_2*v*_ symmetry. Crystallographic data indicate that the protonated porphyrin core forms hydrogen bonding chelates (N-H⋯X⋯H-N) to counter-anions. Weaker interactions, such as induced intramolecular C-H⋯O interactions from the porphyrin periphery are described, with distances characteristic of charge-assisted interactions. This paper offers a conceptual framework for accessing porphyrin macrocycles with designable distortion and symmetry, useful for the selective perturbation of electronic states and a design-for-application approach to solid state porphyrin materials.

## 1. Introduction

Porphyrins (**1**) are key natural compounds with a rich history in synthetic, biological, medicinal, and materials chemistry [1,2,3,4]. They have unique catalytic, electro-, and photochemical properties and can chelate a wide variety of metal ions with the added ability to function as receptors for substrates and analytes [5,6]. In addition, structural aspects of the porphyrin macrocycle conformation play a key role in their in vivo function [7]. Modulation of porphyrin 3D structures gives rise to new structural binding motifs in supramolecular chemistry [8] and can be used to fine-tune their chemical reactivity, metal insertion, and utility as receptors/catalysts [9].

Studies of peripherally crowded porphyrin macrocycles (highly substituted porphyrins) conformations have shown that inducing a dodecasubstitution pattern, such as in 5,10,15,20-tetraaryl-2,3,7,8,12,13,17,18-octaethylporphyrin **2**, often yields structures in which individual pyrrole units are forced from the mean plane of the macrocycle, to adopt a saddled shape [10,11]. Modifications to the substitution regime, such as different steric demands of substituents and the number of *peri*-interaction sites, can tweak the degree of steric crowding and affect macrocycle conformation [12,13]. Pyrrole out-of-plane tilting allows for free base N-H protons, normally locked in a characteristic four-center two-proton hydrogen bond, to form intermolecular H-bonds [9,14]. Nonplanar free base porphyrins have shown great promise as sensors for H-bond acceptor analytes, due to the induced colorimetric change in the porphyrin core upon hydrogen-bond coordination [15]. Additionally, free base nonplanar porphyrins have been investigated as multiple-action catalysts, with Brønsted-acid and -base sites, as well as for their photocatalytic activity [9,16,17,18].

**Figure 1:**
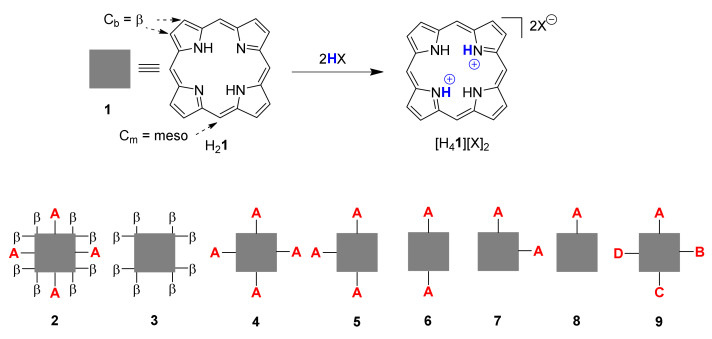
Illustration of porphyrin dication formation and general types of porphyrin substitution patterns.

Core-protonated species, aka porphyrin dications or porphyrindi-ium compounds ([H_4_**1**][X]_2_), are some of the best-known nonplanar porphyrin systems (Figure 1). Their investigation goes back to Fischer’s use thereof in porphyrin analysis [19] and Fleischer’s pioneering studies on the structural flexibility of porphyrins [20]. Over the years we have looked at them in the context of studying highly substituted porphyrins and investigated the additional impact core protonation has on the conformational distortion of nonplanar porphyrins [7,13,21]. However, most of these and related studies [22] used A_4_-type **4**, i.e., 5,10,15,20-tetraphenylporphyrin **10**; symmetric, meso-substituted or β-octasubstituted porphyrins **3,** or the dodecasubstituted systems **2** mentioned above [23,24]. Only limited information is available on porphyrins of the A_x_-series **5**–**8**, i.e., porphyrins with 1–3 meso substituents or unsymmetrical ABCD-type porphyrins **9** [25].

As the first step in a more comprehensive analysis of the structural chemistry of cationic porphyrinoids, protonation of A_2_-type porphyrins **6** was selected as the simplest mechanism of charge introduction, relying on well-established methods for crystal growth [23,24]. Structural analyses of the cations resulting from protonation of all four of the pyrrole nitrogen atoms provide specific information in conformational changes resulting from core modification and general information on the role of charge in porphyrin conformation.

**Figure 2:**
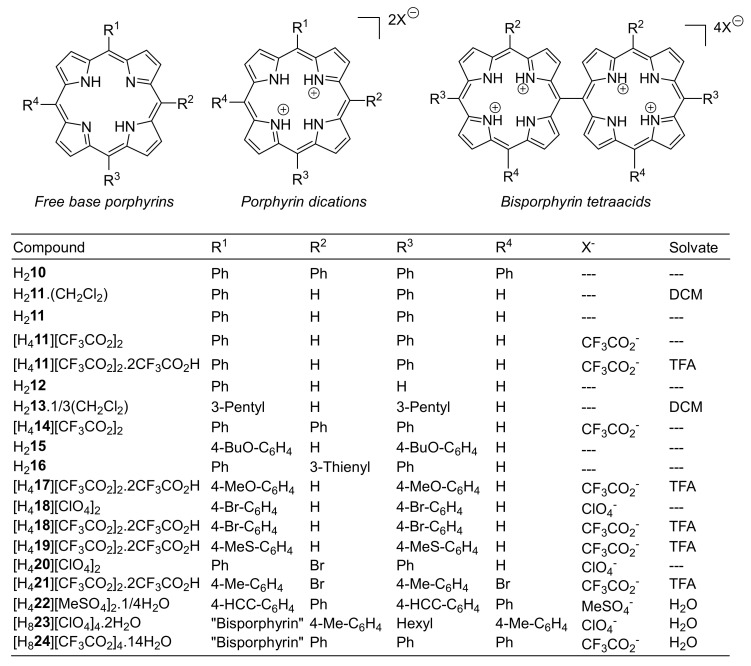
Porphyrins and crystal modifications studied herein.

5,15-Diarylporphyrin compounds are simple, generally air-stable porphyrins, conceptually intermediate between the parent compound of the class, porphyrin (H_2_**1**, Figure 1), and the often used 5,10,15,20-tetraarylporphyrins such as **10** (Figure 2, R^1–4^ = Ph). 5,15-Diarylporphyrins (general formula **6**, Figure 1) are some of the most widely used workhorses in porphyrin chemistry. Some, like 5,15-diphenylporphyrin **11,** are commercially available and/or can be prepared on a large scale [26,27]. They typically serve as starting materials for post-cyclization modification of porphyrins [28,29,30,31,32,33,34] but have also found applications in their own right, e.g., as photosensitizers in photodynamic therapy [35].

The crystal structures of several 5,15-disubstituted free base porphyrins have been previously reported, including the parent 5,15-diphenylporphyrin (H_2_**11**) [36], variously substituted 5,15-diarylporphyrins such as 5,15-bis(*p*-pentyloxyphenyl)porphyrin (AFIFOM) [37], -bis(*o*-aminophenyl)porphyrin (CUYCOQ), and -bis(*p*-carbomethoxyphenyl)porphyrin (AQIQOI), [38] as well as 5,15-dialkylporphyrins (alkyl = ethyl (SAZDUU), *n*-butyl (SAZFAQ) [39]) and 5,15-bis(2-thienyl)porphyrin (REVQIT) [40]. A related structural analysis of free base and diacid mono- (**12**) and diphenylporphyrins has been published, which included the structure of the bistrifluoroacetate of 5,15-bis(3,5-dimethoxyphenyl)porphyrin (MANJAN), the sole prior example of a 5,15-disubstituted porphyrin diacid [25].

As shown in Figure 1, the porphyrin free base can be diprotonated to a porphyrin dication by acids (HX) of sufficient strength to form the dication salt [H_4_Por][X]_2_ [19,20]. The tilting of pyrrole units not only makes the N-H units accessible for external hydrogen bonds it also affects the electronic structure of the aromatic unit and has an associated color change, often from red to green. We present herein a comparative analysis of the crystallographic structural characteristics of porphyrin diacids using data from the CCDC CSD [41] and show and quantify a general structural motif that is consistent among the majority of porphyrin diacids.

## 2. Results and Discussion

### 2.1. Scope of Study

We report 19 crystal structures of compounds bearing an unsymmetrically substituted porphyrin substructure. The focus of this study is on the supramolecular interactions induced by core protonation and the extent to which the conformation of the macrocycle is affected by the introduction of protons and positive charge to a porphyrin ring with an unsymmetric substitution pattern. Hydrogen bonding from the core of each macrocycle is investigated; additionally, weaker intermolecular interactions such as C-H⋯O close contacts from the macrocycle periphery induced by an increasing charge on the macrocycle are discussed. As a model compound for investigating porphyrins with meso- C-H atoms, it is found that protonation of 5,15-diarylporphyrins increases the propensity of meso- and β-C-atoms to act as donors in non-standard hydrogen bonds, and these are investigated. Additional related structures, such as 5,10,15-triphenylporphyrindi-ium bis(trifluoroacetate) and two porphyrin meso-meso linked dimer tetracations, are reported as well.

5,15-Porphyrin diacids are some of the simplest readily accessible porphyrins which have chiral point-group symmetry; these are poised to be important in the enantioselective sensing of chiral analytes. The compounds reported herein expand the number of known porphyrin diacids crystal structures significantly. Where possible, examples have been compared to a similar porphyrin crystal structure in the free base state; this allows a rationalization of the structural effect of protonation on the porphyrin core.

### 2.2. Database Analysis

In order to place the structures reported here in the context of previously reported crystal structures, and to investigate the structural effects of protonation on porphyrin structures, aggregated crystallographic data was analyzed for conformational change and intermolecular interactions. To our knowledge 78 crystal structures of porphyrin diacids of any variety have been reported previously; a full list with CSD codes is given in Table S1.1. Protonation induces intermolecular interactions, conformational changes, and changes to the bond distances and angles of the porphyrin core, summarized here.

**Figure 3:**
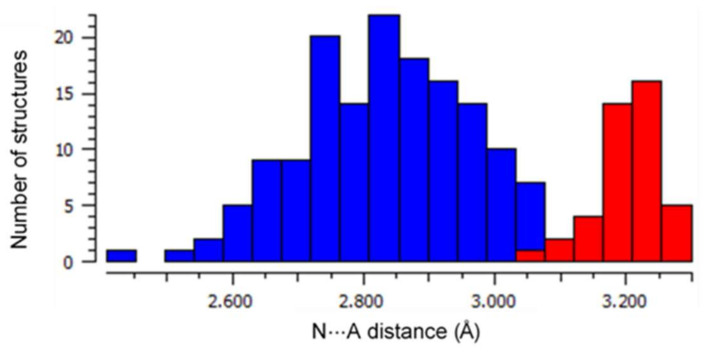
A histogram of the frequency of N⋯A distances for H-bonding N-H⋯A close contacts within porphyrin acid structures in the CSD with A = ^−^OR (in blue) and A = Cl^−^ (in red). Median distances for each of these groups (A = O, 2.846 Å; A = Cl, 3.203 Å) are indicative of charge-assisted H-bond formation.

A common motif is the interaction between the pyrrole N-H and the counter-anions which balances the charge of the porphyrin dication, occurring in all but two of previously reported cases. Counter-anions are often protonating acid species—chloride, trifluoroacetate, and perchlorate—encountered within crystal structures of porphyrin diacids. Hydrogen bonding between the acidic species and anionic guest is ubiquitous for O and Cl acceptors; this charge-assisted hydrogen bond chelate is generally between 0–0.4 Å shorter than the sum of the Van der Waals radii of the donor and acceptor atoms, as can be seen in Figure 3.

Bond distances and angles are affected by protonation of the porphyrin, notably the mean C_a_-N-C_a_ angle, which becomes more obtuse, and the mean N-C_a_-C_b_ angle, which becomes more acute. The N⋯N separation between adjacent pyrroles is generally increased. Tilting of the pyrrole units from the mean plane is observed; whereas free base porphyrin pyrrole subunits are generally coplanar, the pyrrole units in [H_4_]^2+^ porphyrins are generally rotated 20–40° out of the macrocycle mean plane [21,22].

**Figure 4:**
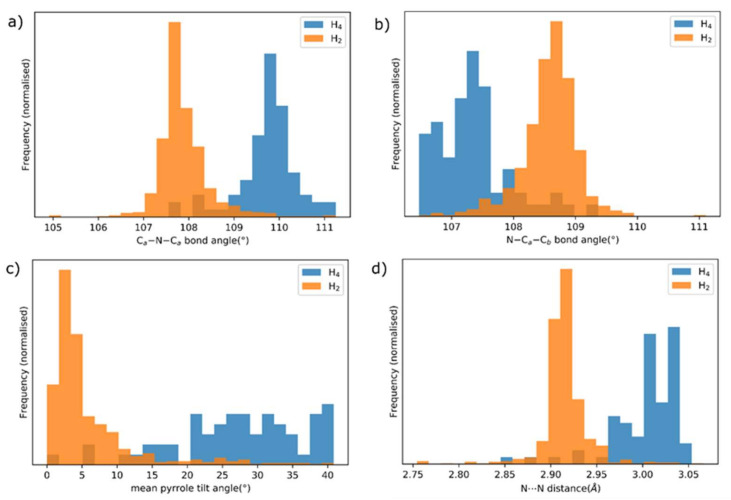
Normalized histograms of selected mean bond distances, angles and derived parameters for the populations of free base porphyrins (orange) and porphyrin dications (blue) for crystal structures reported in the CCDC CSD: (**a**) the C_a_-N-C_a_ angle, (**b**) the N-C_a_-C_b_ angle, (**c**) inclination of the mean plane of each pyrrole from the porphyrin mean plane, (**d**) adjacent N⋯N separation (e.g., N21⋯N22).

These transformations are indicated by obvious changes in the bond distances derived from the populations of the free base and [H_4_]^2+^ porphyrin structures reported in the CCDC Crystal Structure Database; histograms of selected parameters are shown in Figure 4.

### 2.3. Single Crystal X-ray Structure Determinations

#### 2.3.1. 5,15-Diphenylporphyrin and its Dication Salt as Parent Compounds

The crystal structure of 5,15-diphenylporphyrin H_2_**11** crystallized from dichloromethane/hexane, was determined by single crystal X-ray diffraction. In contrast to the previously collected monoclinic diffraction pattern for a sample of the same molecule crystallized from chloroform/hexane [36], the collected diffraction pattern indicated a triclinic cell setting, and a satisfactory solution to the collected data was found in triclinic P$\bar{1}$. A labelled diagram of the atoms within the asymmetric unit of this molecule is presented in Figure S2.2.1.

**Figure 5:**
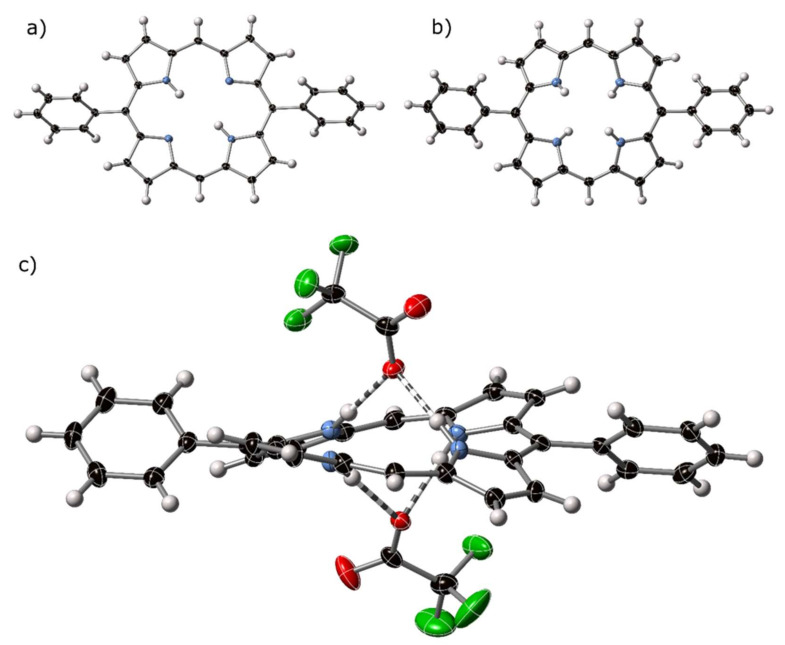
Thermal ellipsoid plots (50%) of the crystal structures of (**a**) H_2_11_triclinic_, (**b**) [H_4_11][CF_3_CO_2_]_2_, with counter anions omitted from view, and (**c**) side view of [H_4_11][CF_3_CO_2_]_2_, with counter anions shown. Hydrogen bonding is indicated by striped bonds.

The asymmetric unit H_2_**11**_triclinic_ shows one half of the 5,15-diphenylporphyrin macrocycle, encompassing two pyrrole subunits, a methine CH, and a phenyl ring; a center of inversion sits at the center of the porphyrin core. A complete molecule in the triclinic form is depicted in Figure 5a. An approximately planar modality can be observed for the porphyrin core, as expected for free-base porphyrins without peripheral steric conflicts. The bond distances and angles are within the expected margins for free base porphyrins [42]. An elongation along the 5,15-axis in this compound can be observed, which is a common motif in the 5,15-porphyrin series [43]; the C_5_⋯C_15_ distance is 7.0778(19) Å vs. 6.703(3) Å for C_10_⋯C_20_.

This compound is a crystalline polymorph of a previously reported structure with the same formula [36]. Both polymorphs appear at low temperatures (150 K for H_2_**11**_mon_ and 100 K for H_2_**11**_tri_) implying that the realignment of the crystals does not occur as a low-temperature phase transition. The monoclinic and triclinic phases have similar C–C and C–N bond distances and angles, as shown in Table S1.4. Minor structural details, such as orientation of phenyl rings with respect to the porphyrin core in H_2_**11**_tri_, are imposed by symmetry constraints for P$\bar{1}$, and cannot be superimposed with their arrangement in the monoclinic form.

Intramolecular aryl-to-porphyrin edge-to-face short contacts are observed, as has been previously observed for H_2_**11**_mon_. These interactions occur on both faces of each molecule of H_2_**11** and form supramolecular chains which extend along the crystallographic *a*-axis. A separation of mean porphyrin planes within this chain, at 3.607(10) Å, is much less than observed in the prior polymorph, at 4.117(10) Å. Additional C–H⋯H–C Van der Waals interactions aggregate these chains into sheets perpendicular to the mean plane of the porphyrin, which extends in the (0 1 1) Miller plane; no significant intermolecular interactions can be observed between sheets. A plot of the molecules within the crystallographic cell is shown in Figure S2.2.1.b.

5,15-Diphenylporphyrin Dichloromethane Solvate–H_2_**11**·CH_2_Cl_2_

The asymmetric unit of the crystal structure of H_2_**11**·CH_2_Cl_2_ shows a molecule of H_2_**11** with similar molecular conformation to that for monoclinic H_2_**11**. This molecule is a remeasurement of a previously reported compound at a lower temperature (108 K versus 150 K) and shows no structural difference to this compound [36]. Relevant plots are shown in Figure S2.2.2.

5,15-Diphenylporphyrindi-ium bis(trifluoroacetate)–[H_4_**11**][CF_3_CO_2_]_2_

The crystal structure of the bis-trifluoroacetate salt of H_2_**11**, 5,15-diphenylporphyrindi-ium bis(trifluoroacetate) [H_4_**11**][CF_3_CO_2_]_2_ was determined by X-ray diffraction; the atoms of the porphyrin component are shown as thermal ellipsoids in Figure 5b and the interaction with counter-anions shown in Figure 5c. Related molecular plots are shown in Figure S2.2.3.

In [H_4_**11**][CF_3_CO_2_]_2_, the pyrrole subunits of the porphyrin dication are tilted out of the central plane of the macrocycle, as expected for porphyrin dications. These protonated pyrroles act as donors in forming hydrogen bonds to trifluoroacetate anions at distances of between 2.761(2) and 2.833(3) Å (N⋯O); each with refinement indicating atom positions centered on nitrogen and N–H⋯O angles of <169°. C–H⋯O interactions can be observed from β- and meso-carbon atoms of one porphyrin to trifluoroacetate anions associated with proximal macrocycles. Non-standard hydrogen-mediated bonding interactions are approximately linear, with distances indicating associative interactions, the shortest of which is 3.064 Å, well below the sum of the Van der Waals radii for C and O. These types of C–H⋯O are common from cationic moieties [44].

5,15-Diphenylporphyrindi-ium bis(trifluoroacetate) trifluoroacetic acid solvate–[H_4_**11**][CF_3_CO_2_]_2_·2CF_3_CO_2_H

A second crystal structure of [H_4_**11**][CF_3_CO_2_]_2_—[H_4_**11**][CF_3_CO_2_]_2_·2CF_3_CO_2_H—was obtained as the trifluoroacetic acid solvate; this compound exhibits a similar conformation, as evidenced by its normal structural decomposition (NSD) profile to [H_4_**11**][CF_3_CO_2_]_2_ (see Section 2.4), and similar bond distances and angles for both porphyrin molecules in the asymmetric unit. Thermal ellipsoid plots and a view of the molecules in the unit cell are shown in Figure S2.2.4. In this structure, the arrangement of porphyrin diacid and counter-anions is identical to the one in [H_4_**11**][CF_3_CO_2_]_2_; each of the trifluoroacetic acid solvates forms a hydrogen bond with a trifluoroacetate anion which is chelated by the porphyrin core. C–H⋯O interactions can be observed from β-carbon atoms, the shortest of which is C318-H318⋯O1B (3.140 Å, 150.7°); these interactions are discussed further in the ‘C–H⋯O Bonding’ Section 2.5.2.

#### 2.3.2. Other 5,15-Disubstituted Porphyrins

The investigation of the cumulative effect of designable distortion in porphyrin molecules necessarily requires accurate determination of more than one 5,15-disubstituted porphyrin diacid crystal structure. The CCDC reference codes of previously reported crystal structures of porphyrin diacid structures are listed in Table S1.1, and reference codes for 5,15-di- and 5,10,15-trisubstituted porphyrins are listed in Table S1.2. The crystal structure of 5,15-bis(3,5-dimethoxyphenyl)porphyrindi-ium ditrifluoroacetate (MANJAN) [25] is the sole previous example of a 5,15-diacid crystal structure and exhibits a similar structure to the 5,15-diphenylporphyrindi-ium structures discussed here.

5,15-Bis(3-pentyl)porphyrin Dichloromethane Solvate–H_2_**13**·⅓CH_2_Cl_2_

**Figure 6:**
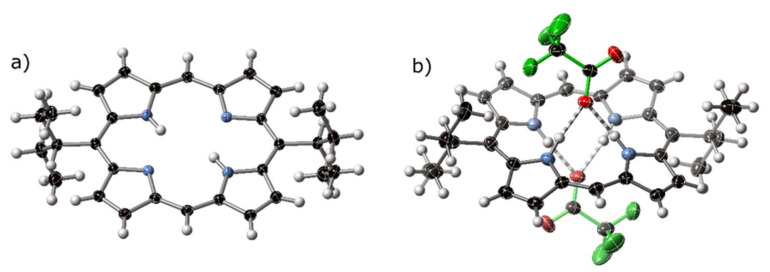
(**a**) View of the molecular structure of H_2_13 in the crystal structure of H_2_13·⅓CH_2_Cl_2_. (**b**) The ‘*cis*’-type hydrogen bonding pattern of [H_4_**13**][CF_3_CO_2_H]_2_. Trifluoroacetate anions are highlighted with green bonds, with H-bonding denoted with striped bonds. Thermal ellipsoids are shown at 50%.

The crystal structure of 5,15-bis(3-pentyl)porphyrin trient(dichloromethane) solvate—H_2_**13**·⅓CH_2_Cl_2_—shown in Figure 6a, crystallizes in a hexagonal cell setting, and could be solved in spacegroup R$\bar{3}$; the asymmetric unit features half of a porphyrin molecule and a 1/6 occupancy disordered DCM molecule. The porphyrin core is approximately planar; alkyl groups are clearly defined at the 5,15-position; these chains project perpendicular to the porphyrin plane. The core of the porphyrin exhibits an exaggerated characteristic 5,15-stretch of 5,15-disubstituted porphyrins [36,43]. C_5_⋯C_15_ separation across the core of this porphyrin is 7.307(4) Å versus 6.481(4) Å for C_10_⋯C_20_. A similarly pronounced meso-stretch has previously been observed for 2,3,5,7,8,12,13,15,17,18-decaalkylporphyrins [45], which are similarly more distorted than 5,15-diphenyl-2,3,7,8,12,13,17,18-octaalkylporphyrin free bases. The in-plane distortion attributed to this compound is exceeded by only a handful of porphyrin structures known, with most of these exceptionally distorted structures resulting from core modification [46,47].

The DCM solvate is situated in a pocket created by three porphyrin molecules above, and three below, arranged by edge-to-face interactions. One orientation of this pocket is shown in supplementary Figure S2.2.5c. DCM is disordered over a six-fold symmetric site at the crystallographic origin; each porphyrin molecule interacts with two solvate pockets, as shown in Figure S2.2.5d.

5,15-Bis(3-pentyl)porphyrindi-ium Bis(trifluoroacetate)–[H_4_**13**][CF_3_CO_2_]_2_

Precipitation of H_2_**13** in acidic solution yields crystals of [H_4_**13**][CF_3_CO_2_]_2_. The alkyl groups on the 5,15-positions of the porphyrin extend perpendicular to the porphyrin mean plane and are disordered over two conformations. This structure exhibits a similarly exaggerated 5,15-stretch of the free base porphyrin H_2_**13**; the C5⋯C5′ separation across the porphyrin core is 7.333(4) Å versus 6.552(5) Å separation for C10⋯C10′ (i.e., C_10_⋯C_20_ due to reduced asymmetric unit).

This compound displays unusual hydrogen bonding for a porphyrin diacid–the neighboring N21, N24-hydrogen atoms are engaged in a hydrogen bonding chelate, as opposed to the N21, N23-chelate observed in all other diacid structures known, with the exception of the tetra(cyclohexyl)porphyrindi-ium we reported some time ago [21]. The H-bonding interaction is shown in Figure 6b; additional crystal structure plots are given in Figure S2.2.6. Similar distortion modes have previously been observed for porphyrin bis(rhodium) chelates [46,47].

Other Crystal Structures of Free Base Porphyrins and Dication Salts

Additionally, the crystal structures of compounds H_2_**15,** H_2_**16**, [H_4_**17**][CF_3_CO_2_]_2_·2CF_3_CO_2_H, [H_4_**18**][ClO_4_]_2_, [H_4_**18**][CF_3_CO_2_]_2_·2CF_3_CO_2_H, [H_4_**19**][CF_3_CO_2_]_2_·2CF_3_CO_2_H, [H_4_**20**][ClO_4_]_2_, [H_4_**21**][CF_3_CO_2_]_2_·2CF_3_CO_2_H, [H_4_**22**][MeSO_4_]_2_·¼H_2_O, [H_8_**23**][ClO_4_]_4_·2H_2_O, and [H_8_**24**][CF_3_CO_2_]_4_·14H_2_O were reported. These molecules are each represented in the skeletal formula shown in Figure 1. Crystallographic data for these compounds is attached as Supplementary Information (SI). The crystal structures of these compounds are depicted in the SI (Sections S2.2.4–S2.2.18), with in-plane and out-of-plane skeletal plots and NSD profile summaries of these crystal structure determinations and selected literature compounds presented in Section S2.3 and Figures S2.3.1–S2.3.22.

**Figure 7:**
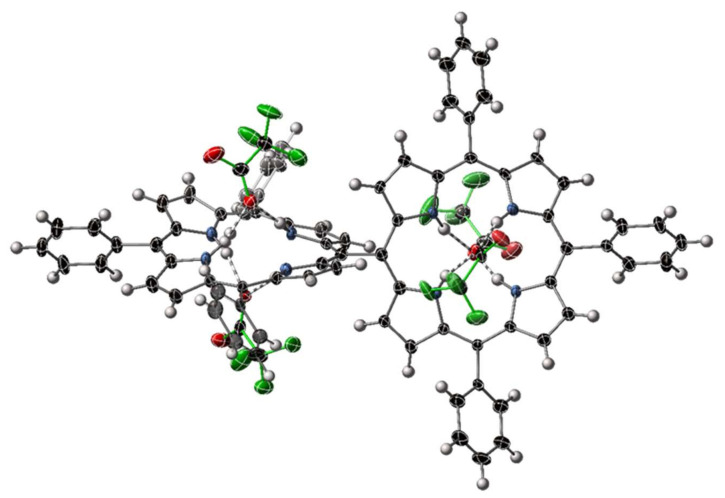
View of an individual molecule of [H_8_**24**][CF_3_CO_2_]_4_ within [H_8_**24**][CF_3_CO_2_]_4_·14H_2_O. Solvents are omitted from this representation; thermal ellipsoids are shown at the 50% level. Trifluoroacetate anions are highlighted with green bonds.

Notable entries in the list are [H_8_**23**][ClO_4_]_4_·2H_2_O and [H_8_**24**][CF_3_CO_2_]_4_·14H_2_O the first examples of structurally characterized bisporphyrin tetracations. The structural features of the individual porphyrin macrocycles are similar to the ‘monoporphyrins’ discussed above. As an example, Figure 7 shows a side view of bisporphyrin tetraacid complex [H_8_**24**][CF_3_CO_2_]_4_.

#### 2.3.3. A_3_-Type Porphyrins

Conceptually intermediate between the 5,15-diaryl and 5,10,15,20-tetraarylporphyrins are porphyrins with three aryl rings at the meso positions—the 5,10,15-triarylporphyrins are represented by general formula **5**. Triarylporphyrins have shown novel reactivity and simple entry to porphyrins of asymmetrical substitution patterns and dimeric species [48,49,50]. Investigation of these species offers the possibility of fine-tuning the reactivity of porphyrin compounds and offers an additional vector for modification.

Crystal structures of triarylporphyrins of general formula **5** (Figure 1) reveal a conformation with characteristics of both the 5,15-diaryl and 5,10,15,20-tetraarylporphyrins. These compounds are listed in Table S1.2, and generally exhibit less pronounced 5,15-stretching (anisotropy) to that observed for 5,15-diarylporphyrins. The crystal structure of the free base parent compound of this class, 5,10,15-triphenylporphyrin has been reported previously [51].

5,10,15-Triphenylporphyrindi-ium bis(trifluoroacetate)–[H_4_**14**][CF_3_CO_2_]_2_

**Figure 8:**
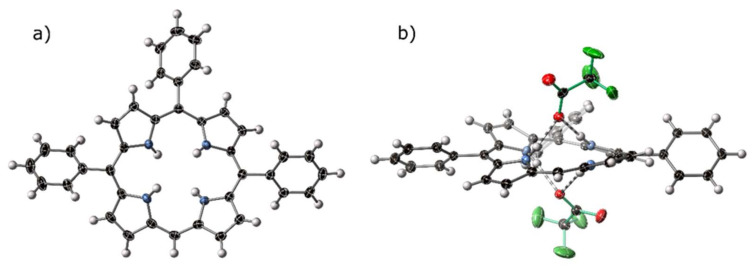
(**a**) A view of the porphyrin component of the crystal structure of [H_4_**14**][CF_3_CO_2_]_2_. (**b**) Hydrogen-bonding bis-chelate to two trifluoroacetate anions within the crystal structure of [H_4_**14**][CF_3_CO_2_]_2_. Trifluoroacetate anions are highlighted with green bonds, hydrogen bonds are indicated with striped bonds, thermal ellipsoids are drawn at 50%.

The title compound ([H_4_**14**][CF_3_CO_2_]_2_) crystallized in a triclinic cell setting, and a solution to the collected diffraction pattern could be obtained in triclinic space group P$\bar{1}$. A view of the porphyrin diacid macrocycle is shown in Figure 8a, with hydrogen-bonding of the core of this macrocycle shown in Figure 8b. A labeled plot of the atoms in the asymmetric unit, as well as a cell packing diagram, are shown in Figure S2.2.7.

The crystal structure of [H_4_**14**][CF_3_CO_2_]_2_ displays the expected hydrogen-bond chelate of porphyrin dications with anionic acceptors, both above and below the plane of the macrocycle. A short contact can be observed from the lone meso- C–H to a trifluoroacetate counter-ion associated with a proximal macrocycle. Standard and non-standard hydrogen bonding behavior is discussed in the following Section 2.4.

With the A_2_-, A_3_-, A_4_-type (A = Ph) porphyrin dications now known, we can compare the effect of incremental addition of aryl rings—the mean out-of-plane distortion (Δ24, deviation from the least-squares-plane of the 24 macrocycle atoms), a common metric for assessing induced nonplanarity, increases nonlinearly from 0.26(4) Å (A_2_)–0.29 Å (A_3_)–0.41(3) Å (A_4_), implying that, with the presence of meso hydrogen atoms, these porphyrins are able to adopt a more planar conformation. This is consistent with the observation of a 5,15-stretch described for 5,15-A_2_- and A_3_-type porphyrins—as nonplanarity is caused by H⋯H repulsion, the rotation of pyrroles in a concerted manner (i.e., that represented by the *B*_2*g*_(1) normal mode presented in Figure S3.1) lessens the out-of-plane deviation required to counteract this repulsion. Measured N⋯N distance across the core (i.e., N_21_–N_23_) remains approximately consistent across this series, 4.22–4.28 Å, even as nonplanar distortion varies considerably.

### 2.4. Normal Structural Decomposition Analysis

Normal-coordinate structural decomposition analysis (NSD) is a method of comparing porphyrin compounds, whereby structural deviations from idealized modality can be quantified by comparison to known distortion modes in each of the symmetry classes for an idealized molecule with D_4h_ symmetry. This method of porphyrin structural analysis was pioneered by Shelnutt and coworkers, based on a least-squares fit of the calculated Raman distortion modes of an idealized, planar molecule [(5,10,15,20-tetraphenylporphyrinato)copper(II)] [52,53]. For metal complexes, the lowest frequency modes of each symmetry operation (6 in-plane, 6 out-of-plane) are usually sufficient to model 90–95% of the structural deviation of metallated, mostly planar porphyrins from normality. This method was used extensively for the analysis and quantification of out-of-plane structure but also yields useful information on the in-plane asymmetric distortions of the porphyrin macrocycle such as that induced by a 5,15-substitution pattern.

NSD methodology thus allows for the investigation of the structural effect of meso-substitution and protonation; nonplanarity and symmetry breaking has been shown to influence both the absorption characteristics and photochemical reactivity of porphyrin compounds [53,54]. With respect to porphyrindi-iums, a multicomponent model is required, due to the 2nd *B*_2*u*_ and 2nd *B*_2*g*_ modes being unusually large for acid porphyrins with the saddle shape, and 5,15-disubstituted porphyrins dependent on β-substitution, respectively. Additionally, the 2nd *B*_2*u*_ mode can quantify the N-H activation of the porphyrin core, an important aspect of sensing with porphyrins acting as H-bond donors. Due to the symmetric nature of the symmetry-breaking modes, these distortional quantifications can be given as the absolute value without changing the chemical interpretation; second ((2)) distortion modes are also normalized by the corresponding primary modes’ mathematical sign.

**Figure 9:**
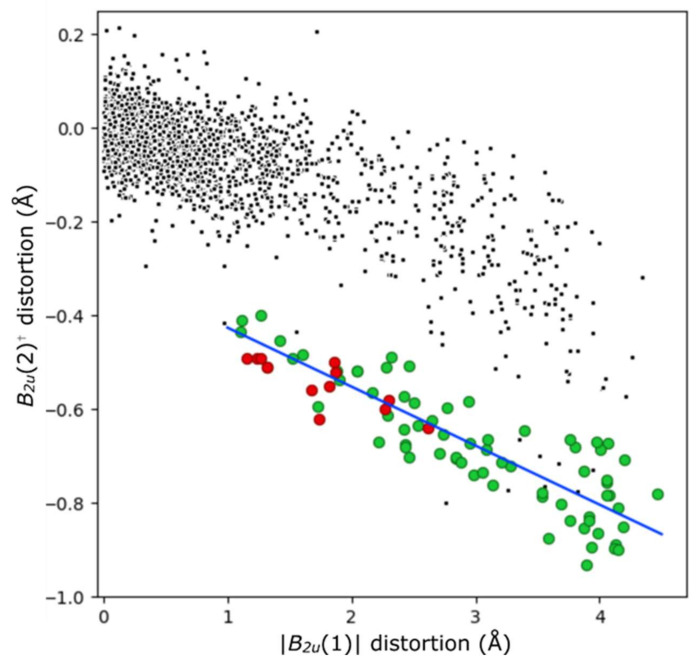
The *B*_2*u*_(1) and *B*_2*u*_(2) distortions in acid (H_4_) porphyrins (green) versus non-cationic porphyrins (black) from crystal structures identified from the CSD. Saddle-shaped diacid compounds reported in this paper are highlighted in red, and the indicated least-squares relation (*B*_2*u*_(2)^†^ = −0.126 (|*B*_2*u*_(1)|) -0.300) with a blue line. The distortion modes are shown in Figure S3.2. ^†^(y-axis) *B*_2*u*_(2) values are multiplied by the sign of the *B*_2*u*_(1) mode; as this mode is symmetrical through the plane, subsequent modes can be aligned to it.

In most cases, and as described above, acid porphyrins are often distorted in a saddled mode. By inducing tilting of pyrrole units in an up-down-up-down manner, N–H atoms avoid steric conflict across the porphyrin core. This rotation additionally allows activated N–H donors to form hydrogen bonds with counter-anions. In NSD terms, this displacement can be modeled by the first two terms of *B*_2*u*_ distortion, which are related by the formula (*B*_2*u*_(2) = −0.126(*B*_2*u*_(1)) −0.300). This relation is shown in Figure 9; the distortion modes involved are shown in Figure S3.2. This out-of-plane distortion mode is not observed for the free base porphyrins reported here to a notable degree, given their planar character. As can be seen in Figure 9, each of the other compounds reported here (indicated with red circles) are consistent with this relation; they are less distorted out-of-plane than the median porphyrin dication. The sole exception, compound [H_4_**13**][CF_3_CO_2_]_2_, possesses centrosymmetry in the solid state due to the previously indicated “*cis*” mode of out-of-plane distortion. This “*cis*” mode is quantified here by *E_g_* distortion aligned with molecular coordinate x and y axes, the summation of which also has *E_g_* symmetry; this molecule approximates *C_2v_* symmetry, with a *C_2_* axis aligned with C_5_–C_15_. 

As noted previously [25] and in the cases presented here, the 5,15-disubstitution pattern of free base porphyrins induces a stretching of the molecules on the 5,15-containing axis. Meso-carbon atoms C_5_ and C_15_ diverge, and C_10_ and C_20_ converge—the meso–meso distances in the 5,15- disubstituted compounds presented here with otherwise free meso-H substitution have a difference in these distances of approximately 0.3A. In NSD terms, this corresponds with the m-stretching first and second mode of *B_2g_* symmetry, which has values between 0.19–0.36 Å for the first mode, and 0.03-0.07 Å for the second. The relevant modes are shown in Figure S3.1. Both modes of *B*_2*g*_ symmetry are exaggerated greatly in compounds of type **3** and **4** if they have alkyl rather than aryl R-groups at the 5- and 15-positions. Distortions along this mode are approximately doubled from the aryl-5,15 structures, to 0.67-0.69 Å for *B*_2*g*_(1) and 0.11–0.16 Å for *B*_2*g*_(2), a reasonable explanation for why this adopts a different mode of out-of-plane distortion.

The combination of a large *B*_2*g*_ (*m-str*) and *B*_2*u*_ (*sad*) mode in 5,15-diarylporphyrin dications (e.g., [H_4_**11**][CF_3_CO_2_]_2_ reduces symmetry of the porphyrin element from the idealized *D*_4h_ to *D*_2_, a chiral group. While previous studies have shown that there would be no persistent chirality among these components allowing for separation, the induction of a favorable intermolecular interaction, such as those towards anions described in the following section, could skew the population of chiral components towards the preferred handedness in the solution state, thus allowing for differential interaction with circularly polarized light. Similarly, in the 5,10,15-triarylporphyrins [H_4_**5**]^2+^, idealized symmetry is reduced further to C_2_; the measured porphyrin NSD profile of [H_4_**14**][CF_3_CO_2_]_2_ is very similar to that of the 5,15-disubstituted molecules, however, indicating that the additional aryl ring imposes minimal structural distortion on the macrocyclic unit. The dication [H_4_**13**]^2+^ in [H_4_**13**][CF_3_CO_2_]_2_ is centrosymmetric, and therefore achiral, but perhaps presents an intermediate state in dicationic porphyrin inversion necessary for spontaneous chiral enrichment.

Induced chirality in the asymmetric acid compounds and deviation from *D*_4h_ symmetry is most likely to affect the absorption bands involving the *A*_1*u*_ -symmetry “*b*2” HOMO in the four-orbital model of porphyrin spectra [55]. Additionally, the presence of the *B*_2*u*_(2) mode is specifically indicative of N–H-activation of the core of the porphyrin. This indicates that 5,15-diarylporphyrin diacids are strong candidates for sensor components for chiral H-bond acceptors in visible light CD spectroscopy.

### 2.5. Intermolecular Interactions

#### 2.5.1. N–H⋯X Bonding from Cationic Porphyrins

Following from the investigation of porphyrin dications reported in the literature, it is expected that the interaction between cationic 5,15-porphyrins and the counter-anions, which balance the charge, will be an important structural motif. Hydrogen-bonding and the formation of a predictable chelate is critical to the use of these compounds as sensor components; porphyrins with core H-atoms able to participate in H-bonding (i.e., not forming the intramolecular characteristic free base porphyrin core arrangement) have been shown to interact with a range of acceptors [9]. This tilting out of the macrocycle plane can be achieved by core protonation or by the introduction of steric bulk around the porphyrin periphery.

**Figure 10:**
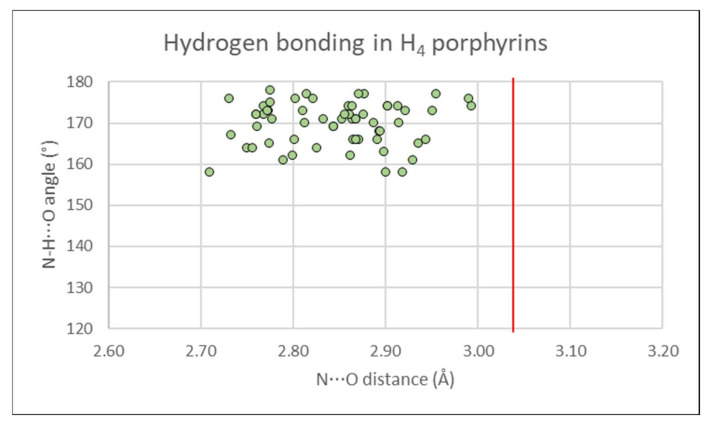
Donor-acceptor distances (2.7–3.0 Å) and D-H⋯X angles (>155°) from porphyrin dication molecules to the counter-anions which balance the charge, in hydrogen-bonding chelates in the crystal structures reported in this paper. A red line indicates the sum of the Van der Waals radii for N and O (3.02 Å).

Each of the free base 5,15-diarylporphyrins show hydrogen-bonding chelates from the core N_21_, N_23_ to one counter-monoanionic (trifluoroacetate, perchlorate, or methylsulfate), matched by an additional chelate on the obverse face. When refined, H-atom electron density is located nearer the N-atom, close to the N⋯O vector. Hydrogen bonds are close to linear (>155°) and well below the sum of the Van der Waals radii of N and O as shown in Figure 10. These are textbook examples of a classical hydrogen-bonding chelate, as previously reviewed in porphyrin structures [56].

In contrast to the 5,15-diaryl structures, the 5,15-dialkyl compound [H_4_**13**][CF_3_CO_2_]_2_ forms a hydrogen-bonding chelate with adjacent pyrroles acting as donors. This approximate shape of porphyrin was observed previously in hexadecahydro-benzoporphyrin, which similarly exhibits a 5,15-axis elongation [21]. The hydrogen-bonding distances of this compound are among the shortest presented here, implying that this interaction is favorable.

#### 2.5.2. C–H⋯O Bonding from Cationic Porphyrins

**Figure 11:**
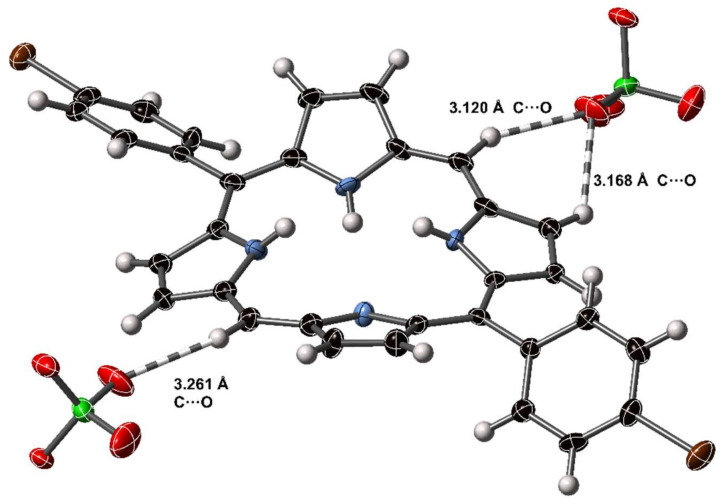
C–H⋯O interactions in the crystal structure of [H_4_**18**][ClO_4_]_2_. Dashed lines indicate H–O close contact interactions; non-H atoms are represented as thermal ellipsoids at 50% probability, H atoms as spheres of fixed radius.

In addition to the conventional hydrogen bonding arrangements of the porphyrin dication core, the periphery of the porphyrin unit is able to engage in non-standard hydrogen-mediated close contacts. As an aromatic unit that becomes charged under acidic conditions, the periphery C–H⋯O interactions are analogous to the peripheral interactions of the pyridinium cation which have been extensively studied [57,58]. To our knowledge, the C–H⋯O interactions of porphyrins have not been specifically investigated, despite often being an important supramolecular motif. The 5,15-porphyrindi-ium dication offers an ideal case, being resplendent with peripheral H-atoms and positive charge, and closely interacting with anionic H-bond acceptors, such as perchlorate. Porphyrin dications are observed to make close, charge-assisted C–H⋯A interactions with anions through β- and meso-carbon atoms. An example from compound [H_4_**18**][ClO_4_]_2_ is shown in Figure 11.

Using the definitions suggested by Steiner [59] for a comparatively strong C–H⋯O interaction, the C–H⋯O atoms would form an approximately linear arrangement around or below the sum of the Van der Waals radii of the donor and acceptor atoms (3.22 Å for C and O). Six such interactions were observed in the group of porphyrindi-ium ions to suitable acceptors; expanding the definition to close contact H⋯A interactions implicates many more interactions with distances of the range 3.06–3.33 Å C⋯O.

The prevalence of C–H⋯O interactions in porphyrin dications implies that porphyrin-containing systems may display similar non-standard hydrogen bonding behavior in transient electronic states where the charge on the core is also high [60]. C–H⋯O interactions are not regularly observed in porphyrin molecules, salts, or complexes without additional charge. With the outlined criteria, we can find 15 porphyrin structures in the CSD with C–H⋯O -bonding, of which 5 have charges on the porphyrin donor. The identified compounds are listed in Table S3.4.

Systematic reviews of C–H⋯A bonding have noted that charge plays a role in the strength of these interactions, as does the presence of large aromatic systems [60]. C-H⋯O bonds, while much weaker than traditional H-bonding, can significantly affect intermolecular packing. As can be seen in this series of compounds, the prevalence of close interactions increases with compound protonation; a plot of distances and angles of these close-contact interactions is presented in Figure S3.3, with a list of these interactions presented in Table S3.5.

While the presence of additional charge on the porphyrin component would likely increase propensity toward H-bond formation, the introduction of suitable anionic acceptors is also a consequence of protonation—the deconvolution of these effects is beyond the scope of this study. From these results, we can clearly observe that charge plays a critical role in the intermolecular interactions of porphyrin units.

## 3. Materials and Methods 

### 3.1. Synthesis

The respective free base porphyrins 5,15-diphenylporphyrin (H_2_**11**) [26], 5,15-bis(3-pentyl)porphyrin (H_2_**13**) [61], 5,10,15-triphenylporphyrin (H_2_**14**) [50], 5,15-bis(4-butoxyphenyl)porphyrin (H_2_**15**) [62], 5,15-diphenyl-10-(3-thienyl)porphyrin (H_2_**16**) [63], 5,15-bis(4-methoxyphenyl)porphyrin (H_2_**17**) [62], 5,15-bis(4-bromophenyl)porphyrin (H_2_**18**) [61], 5,15-bis(4-methylthiophenyl)porphyrin (H_2_**19**) [64], 5-bromo-10,20-diphenylporphyrin (H_2_**20**) [50], 5,15-dibromo-10,20-bis(4-tolyl)porphyrin (H_2_**21**) [65], 5,15-bis(4-ethynylphenyl)-10,20-diphenylporphyrin (H_2_**22**) [66], 10,20,10′20′-tetrakis(4-methoxyphenyl)-15,15′-bis(*n*-hexyl)-5,5′-diporphyrin (H_4_**23**) [67], and 10,15,20,10′,15′,20′-hexaphenyl-5,5′-diporphyrin (H_4_**24**) [68] were prepared by previously reported methods.

Porphyrin dication salts were prepared in situ during the crystal growth from solution, by addition of an excess of trifluoroacetic acid in methanol, aqueous perchloric acid in methanol, or sulfuric acid in methanol layered above a dichloromethane solution of the porphyrin in a capped crystallization tube. Crystals appeared over the course of weeks at the solvent interface.

### 3.2. Crystallography

#### 3.2.1. Instrumentation and Analysis

Crystals were grown by a slow diffusion technique [69]. Table 1, Table 2 and Table 3 and SI section S2.1 give details of the single-crystal X-ray determinations. Diffraction patterns were collected using a Bruker APEX2 DUO or Bruker P4 area detector diffractometer, with Cu_K__α_ or Mo_K__α_ radiation used as indicated in crystal data tabulations (Table 1). Data reduction was performed using the Rigaku CrystalClear [70] and Bruker SAINT programs [71]. Crystal structures were solved using ShelXT [72] and refined using ShelXL [73] with the Shelxle graphical interface [74]. Positional and anisotropic thermal parameters were refined for all non-H atoms except where otherwise indicated. H atoms held to riding U_iso_ parameters; C-bound H atoms were held to riding positions, N- and O-bound H atoms positions were refined and held to DFIX restrictions only where necessary to prevent anomalous refined distances.

Comparison crystal structures were sourced from the CCDC version 2020.1 [41] and compared using data extracted with Conquest [75].

CCDC 2012807–2012825 contain the supplementary crystallographic data for this paper. These data can be obtained free of charge from The Cambridge Crystallographic Data Centre.

#### 3.2.2. Refinement Details

Refinement was performed by the usual method, using restraints where necessary as described in S2.1. Compounds exhibiting disorder were modeled with appropriate sum occupancies and restricted thermal parameters.

### 3.3. Normal Structural Decomposition Analysis

Normal-coordinate decomposition (NSD) analysis was performed to allow for comparison of the distortion modes present within each compound and to investigate symmetry of the porphyrin core. The concept was developed by Shelnutt and coworkers [52,53]; full tables of NSD profiles are presented in Tables and Figures S2.3.1–2.3.22. NSD analyses in this study were performed with a newly developed program using the method of Shelnutt [52]; a description of this new program is currently in preparation; the program is available online at https://chemistry.tcd.ie/staff/people/mos/NSD.html.

## 4. Conclusions

This systematic study of meso-free porphyrin molecules in the acidified [H_4_]^2+^ state has shown that the porphyrin dications adopt two different configurations depending on the meso substituents. N–H atoms reliably form hydrogen bonding chelates with anions, of both the expected common N_21_-N_23_ type as well as the rarer “*cis*” hydrogen bonding chelate. Peripheral meso- and β-C–H donors are induced to form linear, short-range interactions with anionic acceptors. A stretching of the porphyrin along the meso-containing axis is present in both free base and acidified porphyrins to a similar degree; this distortion is present in 5,10,15-triarylporphyrin and related compounds to a lesser degree and exaggerated in 5,15-dipentylporphyrin. Molecules with a large saddle (*B*_2*u*_) and meso-stretch (*B*_2*g*_) deviations from the ideal *D*_4h_ symmetry, such as the diarylporphyrins presented here, are reduced in symmetry to the chiral *D*_2_ point group; these deviations have been quantified using the NSD method. Chiral porphyrins are of ongoing special interest as sensor and optical device components.

## Figures and Tables

**Table 1:** Crystallographic tables for compounds studied.

#	H_2_11	H_2_11·CH_2_Cl_2_	[H_4_11][CF_3_CO_2_]_2_	[H_4_11][CF_3_CO_2_]_2_ ·2CF_3_CO_2_H	H_2_13·⅓(CH_2_Cl_2_)	[H_4_13][CF_3_CO_2_]_2_	[H_4_14][CF_3_CO_2_]_2_	H_2_15
CCCDC #	2012807	2012815	2012814	2012823	2012810	2012809	2012811	2012821
Empirical Formula	C_32_H_22_N_4_	C_33_H_24_Cl_2_N_4_	C_36_H_24_F_6_N_4_O_4_	C_40_H_26_F_12_N_4_O_8_	C_30.33_H_34.67_Cl_0.67_N_4_	C_34_H_36_F_6_N_4_O_4_	C_42_H_28_F_6_N_4_O_4_	C_40_H_38_N_4_O_2_
Formula Weight g·mol^−1^	462.53	547.46	690.59	918.65	478.92	678.67	766.68	606.74
Crystal system	triclinic	monoclinic	monoclinic	monoclinic	trigonal	monoclinic	triclinic	monoclinic
Space Group	P$\bar{1}$	P2_1_/*c*	P2_1_/*c*	P2_1_/*c*	R$\bar{3}$	P2_1_/*c*	P$\bar{1}$	P2_1_/*c*
Z	1	4	4	8	9	2	2	2
λ (Å)	0.71073	0.71075	0.71073	0.71073	0.71075	0.71075	1.54178	0.71073
T (K)	90(2)	108(2)	90(2)	100(2)	108(2)	108(2)	100(2)	90(2)
a (Å)	6.6737(6)	18.190(7)	10.9500(6)	18.0414(17)	19.1340(7)	12.149(4)	10.3071(4)	9.5377(2)
b (Å)	9.7034(9)	13.682(5)	8.3278(5)	37.627(4)	19.1340(7)	10.177(3)	10.4727(4)	18.7751(5)
c (Å)	10.1696(10)	10.688(4)	34.2043(18)	12.3290(12)	18.5994(9)	14.348(4)	16.9482(7)	9.8937(4)
α (°)	61.894(2)	90	90	90	90	90	98.049(2)	90
β (°)	87.318(2)	99.159(6)	94.1210(10)	108.688(2)	90	115.88(2)	96.564(2)	118.8140(10)
γ (°)	76.631(2)	90	90	90	120	90	99.110(2)	90
V (Å^3^)	563.67(9)	2626.1(17)	3111.0(3)	7928.3(13)	5897.1(5)	1596.1(9)	1771.06(12)	1552.32(8)
ρ_calc_ (g·cm^−3^)	1.363	1.385	1.474	1.539	1.214	1.412	1.438	1.298
μ(Mo K_α_), mm^−1^	0.082	0.082	0.279	0.122	0.145	0.137	0.117	0.981
Independent reflections	2934	2934	4623	8371	21398	3847	4335	6004
Data/restraints/parameters	2934/166/0	2934/0/166	4623/2/358	8371/1/463	21398/232/1247	3847/14/185	4335/133/295	6004/0/517
R_1_ (I > 2σ(I))	0.0379	0.0954	0.0588	0.0798	0.0804	0.0621	0.0487	0.042
wR_2_ (all)	0.1082	0.1818	0.1505	0.2282	0.235	0.2237	0.1275	0.1243
GoF	1.029	1.224	0.998	1.298	1.073	0.994	1.009	1.005

**Table 2:** Crystallographic tables for compounds studied.

#	[H_4_17][CF_3_CO_2_]_2_ ·2CF_3_CO_2_H	[H_4_18][ClO_4_]_2_	[H_4_18][ClO_4_]_2_	[H_4_18][CF_3_CO_2_]_2_ ·2CF_3_CO_2_H	[H_4_19][CF_3_CO_2_]_2_ ·2CF_3_CO_2_H	H_2_19	[H_4_20][ClO_4_]_2_
CCCDC #	2012817	2012813	2012812	2012822	2012819	2012808	2012816
Empirical Formula	C_42_H_30_F_12_N_4_O_10_	C_32_H_22_Br_2_Cl_2_N_4_O_8_	C_32_H_22_Br_2_Cl_2_N_4_O_8_	C_40_H_24_Br_2_F_12_N_4_O_8_	C_42_H_30_F_12_N_4_O_8_S_2_	C_36_H_24_N_4_S	C_32_H_22.81_Br_1.19_Cl_2_N_4_O_8_
Formula Weight g·mol^−1^	978.70	821.25	821.25	1076.45	1010.82	544.65	757.46
Crystal system	triclinic	orthorhombic	orthorhombic	triclinic	triclinic	monoclinic	monoclinic
Space Group	P$\bar{1}$	P*bca*	P*bca*	P$\bar{1}$	P$\bar{1}$	P2_1_/*c*	P2_1_/*c*
Z	2	8	8	2	2	4	4
λ (Å)	0.71073	1.54178	1.54178	0.71073	0.71073	1.54178	1.54178
T (K)	108(2)	100(2)	100(2)	100(2)	296(2)	293(2)	100(2)
a (Å)	12.609(4)	14.6001(5)	14.5948(6)	12.691(3)	12.844(3)	12.7897(7)	9.5151(7)
b (Å)	13.290(4)	20.2708(7)	20.2652(8)	13.110(3)	13.378(3)	18.5630(11)	21.4363(15)
c (Å)	13.901(4)	21.2555(8)	21.2737(9)	13.926(3)	14.088(3)	12.9972(8)	15.0162(10)
α (°)	107.622(3)	90	90	107.04(3)	107.629(4)	90	90
β (°)	106.525(2)	90	90	107.06(3)	107.249(4)	119.381(4)	104.174(2)
γ (°)	98.348(3)	90	90	99.59(3)	99.277(5)	90	90
V (Å^3^)	2059.1(11)	6290.7(4)	6292.0(4)	2035.3(9)	2117.8(7)	2688.8(3)	2969.6(4)
ρ_calc_ (g·cm^−3^)	1.579	1.734	1.734	1.756	1.585	1.345	1.694
μ(Mo K_α_), mm^−1^	0.148	5.353	5.352	2.106	0.238	1.326	4.385
Independent reflections	11910	5464	2759	10952	13232	4136	5092
Data/restraints/parameters	11910/4/633	5464/152/485	2759/18/449	10952/2/610	13232/1/633	4136/230/413	5092/1/447
R_1_ (I > 2σ(I))	0.0664	0.0375	0.0333	0.0844	0.0504	0.0474	0.0505
wR_2_ (all)	0.2236	0.1018	0.0771	0.1767	0.1436	0.1341	0.125
GoF	1.028	1.021	1.055	1.188	1.029	0.932	1.253

**Table 3:** Crystallographic tables for compounds studied.

#	[H_4_21][CF_3_CO_2_]_2_·2CF_3_CO_2_H	[H_4_22][MeSO_4_]_2_·¼H_2_O	[H_8_23][ClO_4_]_4_·2H_2_O	[H_8_24][CF_3_CO_2_]_4_·14H_2_O
CCCDC #	2012820	2012818	2012824	2012825
Empirical Formula	C_42_H_28_Br_2_F_12_N_4_O_8_	C_50_H_38.50_N_4_O_8.25_S_2_	C_80_H_82_Cl_4_N_8_O_22_	C_92_H_86_F_24_N_8_O_30_
Formula Weight g·mol^−1^	1104.5	891.47	1649.33	2239.68
Crystal system	monoclinic	triclinic	monoclinic	monoclinic
Space Group	P2_1_/*n*	P$\bar{1}$	C2/*c*	C2/*c*
Z	4	2	4	4
λ (Å)	1.54178	1.54178	0.71073	0.71073
T (K)	100(2)	100(2)	100(2)	100(2)
a (Å)	12.5000(9)	8.0315(4)	28.7894(11)	36.144(11)
b (Å)	16.3340(12)	13.8957(7)	17.0458(6)	13.956(4)
c (Å)	20.9760(14)	20.1428(9)	16.0437(6)	24.094(7)
α (°)	90	99.575(3)	90	90
β (°)	91.671(3)	99.419(3)	94.7000(10)	126.501(5)
γ (°)	90	104.745(3)	90	90
V (Å^3^)	4281.0(5)	2093.07(18)	7846.8(5)	9770(5)
ρ_calc_ (g·cm^−3^)	1.714	1.414	1.396	1.523
μ(Mo K_α_), mm^−1^	3.41	1.687	0.232	0.142
Independent reflections	7322	6701	9042	8925
Data/restraints/parameters	7322/1/636	6701/741/673	9042/297/612	8925/196/696
R_1_ (I > 2σ(I))	0.0313	0.0460	0.0448	0.1181
wR_2_ (all)	0.082	0.1259	0.129	0.3597
GoF	1.037	1.037	1.070	1.687

## Supplementary Materials

The following are available online, Section S2.1; Details of disorder and deviation from general crystallographic procedure. Table S1.1; Porphyrin diacid CCDC structure codes. Table S1.2; 5,15-disubstituted and 5,10,15 trisubstituted porphyrin freebase structure codes. Table S3.4; non-standard C-H⋯O close contacts in previously reported porphyrin molecules from the CCDC CSD. Table S3.5; non-standard C-H⋯X (X = O, S, F) close contacts from the porphyrin core in porphyrindi-ium molecules reported in this paper. Figures S2.2.1–S2.2.18; crystal structure images. Figures S2.3.1–S2.3.22; NSD profiles and skeletal plots derived from crystal structure determinations. Figure S3.1 and S3.2; Defined normal modes of porphyrin distortion. Figure S3.3; A plot of distances and angles of nonstandard hydrogen bonding from porphyrin dications.
