# Supplementary material for: Weak Interactions and Conformational Changes in Core-Protonated A2- and Ax-Type Porphyrin Dications
Source: Molecules. 2020 Jul 13;25(14):3195. doi: 10.3390/molecules25143195 (PMC7397311; doi:10.3390/molecules25143195)
Supplement: Supplementary file 1 [file molecules-25-03195-s001.pdf]

Supporting information for
Weak interactions and conformational changes in core protonated A_2- and A_x-type porphyrin dications

Christopher J. Kingsbury, Keith J. Flanagan, Hans-Georg Eckhardt, Marc Kielmann and Mathias O. Senge*

Contents

S1. Tables of structure codes referenced in the statistical analyses 4

1.1 Table S1.1; Porphyrin diacid CCDC structure codes 4

1.2 Table S1.2. 5,15-disubstituted and 5,10,15-trisubstituted porphyrin free base structure codes 5

S2. Single Crystal X-ray diffraction studies 6

2.1 Details of disorder and deviation from general crystallographic procedure 6

2.1.1 General disorder modelling 6

2.1.2 H_2**11**·CH_2Cl_2 5,15-Diphenylporphyrin DCM 6

2.1.3 [H_4**11**][CF_3CO_2]$_2$ 5,15-Diphenylporphyrindi-ium bis(trifluoroacetate) 6

2.1.4 [H_4**11**][CF_3CO_2]$_2$·2CF_3CO_2H 5,15-Diphenylporphyrindi-ium bis(trifluoroacetate) bis(trifluoroacetic acid solvate) 6

2.1.5 H_2**13**·⅓(CH_2Cl_2) 5,15-Bis(3-pentyl)porphyrin 1/3(DCM) 6

2.1.6 [H_4**13**][CF_3CO_2]$_2$ 5,15-Bis(3-pentyl)porphyrindi-ium bis(trifluoroacetate) 6

2.1.7 [H_4**17**][CF_3CO_2]$_2$·2CF_3CO_2H 5,15-Bis(4-methoxyphenyl)porphyrindi-ium bis(trifluoroacetate) bis(trifluoroacetic acid solvate) 7

2.1.8 [H_4**18**][ClO_4]$_2$ 5,15-bis(4-bromophenyl)porphyrindi-ium bis(perchlorate) 7

2.1.9 H_2**19** 5,15-Diphenyl-10-(thiophen-3-yl)porphyrin 7

2.1.10 [H_4**20**][ClO_4]$_2$ 5-Bromo-10,20-diphenylporphyrindi-ium diperchlorate 7

2.1.11 [H_4**21**][CF_3CO_2]$_2$·2CF_3CO_2H 5,15-Dibromo-10,20-bis(4-tolyl)porphyrindi-ium bis(trifluoroacetate) bis(trifluoroacetic acid) solvate 7

2.1.12 [H_4**22**][$MeSO_4$]$_2$·¼H_2O 5,15-Bis(4-ethynylphenyl)-10,20-diphenylporphyrindi-ium bis(methylsulfate) hemiaqua solvate 7

2.1.13 [H_8**23**][ClO_4]$_4$·2H_2O 5,5'-Bis(15-hexyl-10,20-bis(4-methoxyphenyl)porphyrindi-ium) tetrakis(perchlorate) diaqua solvate 7

2.1.14 [H_8**24**][CF_3CO_2]$_4$·14H_2O 5,5'-Bis(10,15,20-triphenylporphyrindi-ium) tetrakis(trifluoroacetate) 7

2.2 Plots of individual crystal structures 9

2.2.1 Figure S2.2.1; H_2**11** (5,15-diphenylporphyrin) 9

2.2.2 Figure S2.2.2; H_2**11**·CH_2Cl_2 (5,15-diphenylporphyrin) DCM 10

2.2.3 Figure S2.2.3; [H_4**11**][CF_3CO_2]$_2$ 5,15-diphenylporphyrindi-ium bis(trifluoroacetate).... 11

2.2.4 Figure S2.2.4; $[H_4$**11**$][CF_3CO_2]_2 \cdot 2CF_3CO_2H$ 5,15-diphenylporphyrindi-ium bis(trifluoroacetate)bis(trifluoroacetic acid solvate) 13

2.2.5 Figure S2.2.5; H_2**13**$\cdot$⅓(CH_2Cl_2) 5,15-bis(3-pentyl)porphyrin 1/3(DCM) 15

2.2.6 Figure S2.2.6; $[H_4$**13**$][CF_3CO_2]_2$ 5,15-bis(4-(3-pentyl))porphyrindi-ium bis(trifluoroacetate) 17

2.2.7 Figure S2.2.7; $[H_4$**14**$][CF_3CO_2]_2$ 5,10,15-triphenylporphyrindi-ium bis(trifluoroacetate) 19

2.2.8 Figure S2.2.8; H_2**15** 5,15-bis(4-butoxyphenyl)porphyrin 21

2.2.9 Figure S2.2.9; $[H_4$**17**$][CF_3CO_2]_2 \cdot 2CF_3CO_2H$ 5,15-bis(4-methoxyphenyl)porphyrindi-ium bis(trifluoroacetate) bis(trifluoroacetic acid solvate) 22

2.2.10 Figure S2.2.10; $[H_4$**18**$][ClO_4]_2$ 5,15-bis(4-bromophenyl)porphyrindi-ium bis(perchlorate) 24

2.2.11 Figure S2.2.11; $[H_4$**18**$][CF_3CO_2]_2 \cdot 2CF_3CO_2H$ 5,15-bis(4-bromophenyl)porphyrindi-ium bis(trifluoroacetate) bis(trifluoroacetic acid solvate) 26

2.2.12 Figure S2.2.12; $[H_4$**19**$][CF_3CO_2]_2 \cdot 2CF_3CO_2H$ 5,15-bis(4-methylthiophenyl)porphyrindi-ium bis(trifluoroacetate) bis(trifluoroacetic acid) solvate 28

2.2.13 Figure S2.2.13; H_2**16** 5,15-diphenyl-10-(thiophen-3-yl)porphyrin 30

2.2.14 Figure S2.2.14; $[H_4$**20**$][ClO_4]_2$ 5-bromo-10,20-diphenylporphyrindi-ium diperchlorate32

2.2.15 Figure S2.2.15; $[H_4$**21**$][CF_3CO_2]_2 \cdot 2CF_3CO_2H$ 5,15-dibromo-10,20-bis(4-tolyl)porphyrindi-ium bis(trifluoroacetate) bis(trifluoroacetic acid) solvate 34

2.2.16 Figure S2.2.16; $[H_4$**22**$][MeSO_4]_2 \cdot$¼H_2O 5,15-bis(4-ethynylphenyl)-10,20-diphenylporphyrindi-ium bis(methylsulfate) hemiaqua solvate 36

2.2.17 Figure S2.2.17; $[H_8$**23**$][ClO_4]_4 \cdot 2H_2O$ 5,5'-Bis(15-hexyl-10,20-bis(4-methoxyphenyl)porphyrindi-ium) tetrakis(perchlorate) diaqua solvate 38

2.2.18 Figure S2.2.18; $[H_8$**24**$][CF_3CO_2]_4 \cdot 14H_2O$ 5,5’-bis(10,15,20-triphenylporphyrindi-ium) tetrakis(trifluoroacetate) 41

2.3 Normal-coordinate Structural Decomposition (NSD) 44

2.3.1 NSD result generated from H_2**11** (5,15-diphenylporphyrin) 45

2.3.2 NSD result generated from H_2**11**$\cdot CH_2Cl_2$ (5,15-diphenylporphyrin DCM) 46

2.3.3 NSD result generated from $[H_4$**11**$][CF_3CO_2]_2$ (5,15-diphenylporphyrindi-ium bis(trifluoroacetate)) 47

2.3.4 NSD result generated from $[H_4$**11**$][CF_3CO_2]_2 \cdot 2CF_3CO_2H$ (5,15-diphenylporphyrindi-ium bis(trifluoroacetate) bis(trifluoroacetic acid solvate)) 48

2.3.5 NSD result generated from H_2**13**$\cdot$⅓(CH_2Cl_2) (5,15-bis(3-pentyl)porphyrin 1/3(DCM)) 49

2.3.6 NSD result generated from $[H_4$**13**$][CF_3CO_2]_2$ (5,15-bis(3-pentyl)porphyrindi-ium bis(trifluoroacetate)) 50

2.3.7 NSD result generated from $[H_4$**14**$][CF_3CO_2]_2$ (5,15-triphenylporphyrindi-ium bis(trifluoroacetate)) 51

2.3.8 NSD result generated from H_2**15** (5,15-bis(4-butoxyphenyl)porphyrin) 52

2.3.9 NSD result generated from [H_4**17**][CF_3CO_2]$_2$·2CF_3CO_2H (5,15-bis(4-methoxyphenyl)porphyrindi-ium bis(trifluoroacetate) bis(trifluoroacetic acid solvate)) 53

2.3.10 NSD result generated from [H_4**18**][ClO_4]$_2$ (5,15-bis(4-bromophenyl)porphyrindi-ium bis(perchlorate)) (HE) 54

2.3.11 NSD result generated from [H_4**18**][ClO_4]$_2$ (5,15-bis(4-bromophenyl)porphyrindi-ium bis(perchlorate)) (MS) 55

2.3.12 NSD result generated from [H_4**18**][CF_3CO_2]$_2$·2CF_3CO_2H (5,15-bis(4-methylthiophenyl)porphyrindi-ium bis(trifluoroacetate) bis(trifluoroacetic acid) solvate) (HE) 56

2.3.13 NSD result generated from [H_4**18**][CF_3CO_2]$_2$·2CF_3CO_2H (5,15-bis(4-methylthiophenyl)porphyrindi-ium bis(trifluoroacetate) bis(trifluoroacetic acid) solvate) (MS) 57

2.3.14 NSD result generated from H_2**19** (5,15-diphenyl-10-(thiophen-3-yl)porphyrin) 58

2.3.15 NSD result generated from [H_4**20**][ClO_4]$_2$ (5-bromo-10,20-diphenylporphyrindi-ium diperchlorate) 59

2.3.16 NSD result generated from file [H_4**21**][CF_3CO_2]$_2$·2CF_3CO_2H (5,15-dibromo-10,20-bis(4-tolyl)porphyrindi-ium bis(trifluoroacetate)bis(trifluoroacetic acid) solvate) 60

2.3.17 NSD result generated from [H_8**23**][ClO_4]$_4$·2H_2O (5,5'-Bis(15-hexyl-10,20-bis(4-methoxyphenyl)porphyrindi-ium) tetrakis(perchlorate) diaqua solvate) 61

2.3.18 NSD result generated from [H_4**22**][$MeSO_4$]$_2$·¼H_2O (5,15-bis(4-ethynylphenyl)-10,20-diphenylporphyrindi-ium bis(methylsulfate) hemiaqua solvate) 62

2.3.19 NSD result generated from [H_4**18**][CF_3CO_2]$_2$·2CF_3CO_2H (5,15-bis(4-bromophenyl)porphyrindi-ium bis(trifluoroacetate) bis(trifluoroacetic acid) solvate) 63

2.3.20 NSD result generated from [H_8**24**][CF_3CO_2]$_4$·14H_2O (5,5'-bis(10,15,20-triphenylporphyrindi-ium) tetrakis(trifluoroacetate) aqua solvate) 64

2.3.21 NSD result generated from CCDC entry ODEFOS (H_2**11** (monoclinic), 5,15-diphenylporphyrin) 65

2.3.22 NSD result generated from CCDC entry MOSQEQ (H_2**14** 5,10,15-triphenylporphyrin) 66

S3. Supplemental Images and tables 67

S1. Tables of structure codes referenced in the statistical analyses

Crystal structures are available from https:/ccdc.cam.ac.uk/structures

1.1 Table S1.1; Porphyrin diacid CCDC structure codes

Index	CCDC Refcode	Index	CCDC Refcode
1	ANIHIQ	41	PEZXAW
2	ARORUV	42	QARCAQ
3	ASUNAD	43	QEZKIP
4	BASJUA	44	QOLPAK
5	BEJDIG	45	QOSYUT
6	CETPEX	46	QURRAY
7	FARBEI	47	RALVAC
8	FATQUP	48	RARQEJ
9	FIMRAV	49	RATXUI
10	FOKZUC	50	REVROZ
11	GALROC	51	RUHQAM
12	GEFLOU	52	RUHQEQ
13	GOBSOF	53	RUHQIU
14	GOBYIF	54	SEPVIT
15	GUZMUJ	55	TIQLAH
16	KEVDAT	56	TIQLEL
17	KIBLIQ	57	TIQLIP
18	KIBMAJ	58	TPPFEC
19	KIBMEN	59	TPYPRC10
20	KIBPEQ	60	VACSIC
21	KIBPEQ01	61	VOGZAT
22	LEXSIQ	62	WINXEW
23	LEYFOK	63	WIXZAF
24	LEYFUQ	64	WUKBOT
25	LEYHIG	65	XAQKOR
26	LEYPEK	66	XARVIW
27	LEYQAH	67	XEDFOD
28	LOGMOJ	68	XEDFUJ
29	LOLPOR	69	XEDGAQ
30	MANHOZ	70	XEDGEU
31	MANHUF	71	XEKDEZ
32	MANJAN	72	YEVJAN
33	MIGNEW	73	YEVKAL
34	NICPAR	74	YEVKIT
35	NICPEV	75	YEVKIT01
36	NUFTEO	76	YEVKOZ
37	NUHKUW	77	YEVKUF
38	OCAQAN	78	WIXDIT
39	OCIQEY		
40	PACXEY		

1.2 Table S1.2. 5,15-disubstituted and 5,10,15-trisubstituted porphyrin free base structure codes

**Index**	**CCDC Refcode**	**5,15-(R)=**	**|$B_{2g}(1)$| parameter**
Free bases			
1	AFIFOM	4-OBu-C_6H_4	0.32
2	AQIQOI	4-(CO_2Me)-C_6H_4	0.43
3	BASDUX	4-OOct-C_6H_4	0.32
4	CUYCOQ	2-NH_2-C_6H_4	0.27
5	EDEVER	4-CO_2(H, Ca)-C_6H_4	0.29
6	EDEVUH	4-CO_2(H, Mg) -C_6H_4	0.25
7	IMAXAV	2-MeOMe-C_6H_4	0.26
8	ISECAM	Mesityl	0.25
9	ISECAM01	Mesityl	0.23
10	KAPCIO	4-OOc-C_6H_4	0.26
11	KIPWOX	4-O*i*-Pr-C_6H_4	0.23
12	KIPWUD	4-O*i*-Pr-C_6H_4	0.23
13	NENHAS	Strapped	0.27
14	ODEFOS	Ph	0.42
15	ODEFUY	Ph (+DCM)	0.35
16	PAMZIO	4-Tolyl/4-OBu-C_6H_4	0.29
17	QOSQOF	4-(O-Hx-6-SAc)-C_6H_4	0.26
18	SIVTEX	2,6-di(OHx)-C_6H_3	0.29
19	UGACOW	Terphenyl oxoborate dimer	0.30
20	ZAQSUF	Strapped	0.17
21	ZAQTAM	Strapped	0.14
22	ZAQTEQ	Strapped	0.17
23	GOBBAB	Py	0.31
24	GOBBEF	$PyCH_3^+(OTs^-)_2$	0.37
25	REVQIT	2-Thiophene	0.48
26	SAZDUU	Et	0.38
27	SAZFAC	*n*Bu	0.36
5,15- Diacid			
1	MANJAN	3,5-di(MeO)-C_6H_3	0.11
5,10,15-triaryl freebases		**Name**	
1	BEDPIM	2-Iodo-5,10,15-triphenylporphyrin	0.13
2	BEDPOS	2-bromo-5,10,15-triphenylporphyrin	0.15
3	EFALAZ	5,10,15-tri(3,5-di-tert-butylphenyl)-2,18-di((O-hexyl)-3-acrylate)-porphyrin	0.23
4	EFALIH	5,10,15-tri(3,5-di-tert-butylphenyl)-2,18-di(octan-3-one) -porphyrin	0.26
5	MOSQEQ	5,10,15-triphenylporphyrin	0.21
6	OYIHUB02	(2-bis(trimethylsilyloxy)methylsilanyl)-5,10,15-triphenylporphyrin	0.14
7	QOQGOS	2,3,7,8,12,13,17,18-octaethyl-5,10,15-triphenylporphyrin	0.13
8	UQUQEE	5,10,15-tri(3,5-di-tert-butylphenyl)porphyrin	0.10
9	QIYSEZ	5,15-bis(mesityl)-10-(xanthene derivative)-porphyrin	0.02
10	QIYSOJ	5,15-bis(mesityl)-10-(xanthene derivative)- porphyrin	0.08

S2. Single Crystal X-ray diffraction studies

The general procedure for X-ray analysis is outlined in the methods section of the main paper.

2.1 Details of disorder and deviation from general crystallographic procedure

2.1.1 General disorder modelling

Disorder was modelled as described in Section 3.2.1 of the main text.

Reflections were omitted when, on the advice of CheckCIF, the values presented were indicative of stark disagreement with the model, and a missed modelling in the data reduction step or an outlier.

EADP were used when two atoms of the same assignment were observed to have overlapping ellipsoids and shared a site (i.e. non-bimodal assigned electron density from the e- density map). EXYZ were also used in these cases to prevent some overfit of strong high-angle data, a consequence of free refinement of Fourier-type data.

SIMU were used for refined disorder over a two- or three-site model to constrain over-modelling. SUMP was used in three-component modelling to restrict all components to a sum occupancy of 1.

H-atoms were constrained (AFIX n3) on C, and free on N, unless otherwise indicated. Weak DFIX commands (sigma ca. 0.1 angstroms) were used to define visually identified hydrogen bonding with solvent. O-H···O H-atom distances between trifluoroacetic acid and trifluoroacetate anions, assumed to have ‘symmetric hydrogen-bond’ character due to the similarity between the donor and acceptor, were often required to be fixed using a DFIX command. Water solvent was modelled in each case as constrained with DFIX commands for O-H and H-H distances, with hydrogen bonds defined as described for other solvent.

Specific computer instructions used are available in the _shelx_res_file section of the CIF supplementary information.

2.1.2 H_2**11**$\cdot CH_2Cl_2$ 5,15-Diphenylporphyrin DCM

N21-H21 and N23-H23 were constrained with DFIX

2.1.3 [H_4**11**][CF_3CO_2]$_2$ 5,15-Diphenylporphyrindi-ium bis(trifluoroacetate)

N21-H21 was constrained with DFIX

2.1.4 [H_4**11**][CF_3CO_2]$_2\cdot 2CF_3CO_2H$ 5,15-Diphenylporphyrindi-ium bis(trifluoroacetate) bis(trifluoroacetic acid solvate)

Several low angle reflections (1 0 0, 1 1 0, 3 0 0, 1 1 1) were omitted due to presumptive interference with the beamstop. Two of the eight N-H bonds in two non-symmetrically equivalent porphyrins in the asymmetric unit were constrained with DFIX. A trifluoroacetatic acid molecule was modelled as disordered over two orientations (O1B -F7B (77%) and O1C-F7C (23%)) and this was constrained with SIMU.

2.1.5 H_2**13**$\cdot$⅓(CH_2Cl_2) 5,15-Bis(3-pentyl)porphyrin 1/3(DCM)

DCM solvent was constrained with SIMU and DFIX to idealised C-Cl distances. This solvent was disordered about a six-fold center of symmetry.

2.1.6 [H_4**13**][CF_3CO_2]$_2$ 5,15-Bis(3-pentyl)porphyrindi-ium bis(trifluoroacetate)

The pentyl group at the 5- position was modelled as disordered over two positions (C51-C56 and C61-C66, 0.86:0.14 with hydrogens) as was the trifluoromethyl component of the trifluoroacetate anion (C2A,F1A-F3A and C2B,F1B-F3B, 0.89:0.11). Reflection -3 1 2 was omitted due to stark disagreement with the model.

2.1.7 [H_4**17**][CF_3CO_2]$_2$·2CF_3CO_2H 5,15-Bis(4-methoxyphenyl)porphyrindi-ium bis(trifluoroacetate) bis(trifluoroacetic acid solvate)

5 reflections were omitted sue to interference with the beamstop, and a further 19 due to negative intensities from overzealous background correction. O-H⋯O H-atom distances between trifluoroacetic acid and trifluoroacetate anions were DFIXed.

2.1.8 [H_4**18**][ClO_4]$_2$ 5,15-bis(4-bromophenyl)porphyrindi-ium bis(perchlorate)

Perchlorate was modelled as disordered over two orientation sharing approximate Cl position. The perchlorate molecules (Cl1A & O1A-O4A, 20%, Cl1B & O1B-O4B, 80%) were constrained to a sum occupancy of 1 and to similar thermal ellipsoids using a SIMU command.

2.1.9 H_2**19** 5,15-Diphenyl-10-(thiophen-3-yl)porphyrin

The thiophene at the 10-position of the porphyrin ring was modelled as disordered over two orientations (C101-C102-S103-C104-C105, 62% and C101-C106-S107-C108-C109, 38%), representing approx. 180° rotation of the thiophene around the thiophene-porphyrin bond. SADI commands were used to constrain the C-S, C-C, and C=C bonds in the thiophene to equivalent values. Reflections 2θ > 135° were omitted.

2.1.10 [H_4**20**][ClO_4]$_2$ 5-Bromo-10,20-diphenylporphyrindi-ium diperchlorate

N24-H24A was constrained with DFIX. The hydrogen (H5 and H15) and bromine atoms (Br1 and Br2 respectively) were constrained to a sum occupancy of 1 at each site, however the two bromine atoms had a sum occupancy which exceeded 1, due to an impurity of 5,15-dibromo-10,20-diphenylporphyrindi-ium diperchlorate accounting for 19.1% of the molecules within the crystal.

2.1.11 [H_4**21**][CF_3CO_2]$_2$·2CF_3CO_2H 5,15-Dibromo-10,20-bis(4-tolyl)porphyrindi-ium bis(trifluoroacetate) bis(trifluoroacetic acid) solvate

N22-H22 was constrained with DFIX

2.1.12 [H_4**22**][$MeSO_4$]$_2$·¼H_2O 5,15-Bis(4-ethynylphenyl)-10,20-diphenylporphyrindi-ium bis(methylsulfate) hemiaqua solvate

Both of the methylsulfate anions in the asymmetric unit were modelled as disordered over two orientations (S2A,O5A-O8A and C50A, 52%, S2B,O5B-O8B and C50B, 48%) and (S1A, O1A-O4A and C49A, 92%, S1B, O1B-O4B and C49B, 8%); SADI, SIMU (between bonded atoms) and EADP (equivalent atoms) commands were used to constrain these two orientations to hold equal thermal parameters and bond distances in each case.

2.1.13 [H_8**23**][ClO_4]$_4$·2H_2O 5,5'-Bis(15-hexyl-10,20-bis(4-methoxyphenyl)porphyrindi-ium) tetrakis(perchlorate) diaqua solvate

The alkyl chain at the 15 position of the porphyrin ring was modelled as disordered over 3 orientations, sharing the positions C151 and C152 (100%), and disordered C153-C156 (63%) C163-C166 (26%) and C173-C176 (11%) constrained to a total occupancy of 1.0000(1) with a SUMP command. SIMU and DFIX commands were used to constrain the alkyl chain to a sensible geometry. Three reflections (1 1 0, 4 0 2, 4 2 1) were omitted because of disagreement with the model.

2.1.14 [H_8**24**][CF_3CO_2]$_4$·14H_2O 5,5’-Bis(10,15,20-triphenylporphyrindi-ium) tetrakis(trifluoroacetate)

Squeeze routine in PLATON was used to account for electron density from disordered water molecules. Porphyrin core hydrogen-bonding interactions (D-H⋯A, D = {N21,N22,N23,N24}) were fixed from both D-H and H⋯A in all cases due to instability of the H-atom position to refinement. This was considered preferable to fixed positional refinement, given the importance of hydrogen bonding patterns to this paper. 8 atoms {C5 C6 N22 C2B O1W O2W C12 C201 C154} had to be constrained as approximately isotropic due to lying near sites of crystallographic Fourier ripples from

the origin, which caused collapse of the ellipsoid. The trifluoromethyl component of a trifluoroacetate anion was modelled as disordered over two positions (F5A-F7A and C2A (73%) / F5B-F7B and C2B (27%)) with SIMU to constrain thermal parameters. Solvent water was modelled as two rotating rigid molecules of fractional occupancy (35% and 31%); including disordered water molecules accounted for by SQUEEZE a total of 56 water molecules were identified in the unit cell (Z = 4). 6 reflections were omitted due to disagreement with the model.

2.2 Plots of individual crystal structures

2.2.1 Figure S2.2.1; H_2**11** (5,15-diphenylporphyrin)

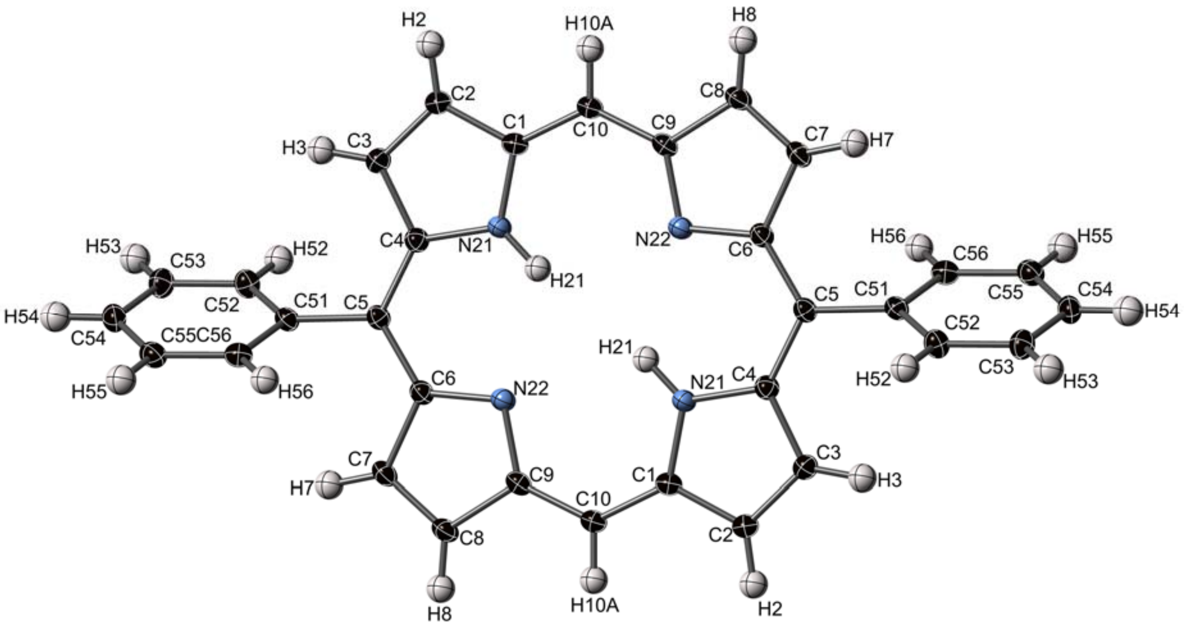

a) A labelled plot of the atoms within the asymmetric unit of compound H_2**11**. Thermal ellipsoids are shown at 50% probability level.

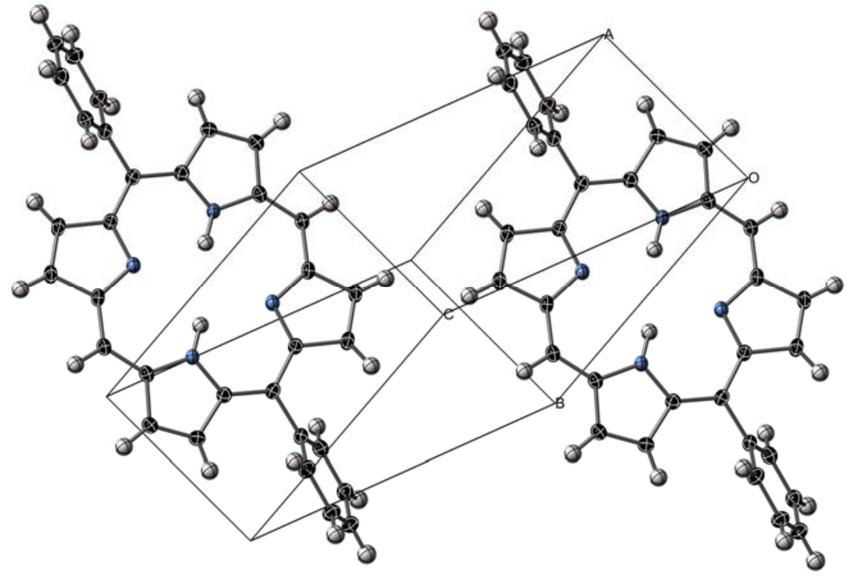

b) A plot of the molecules which comprise the unit cell of the crystal structure of compound H_2**11** (Z = 4). Thermal ellipsoids are shown at 50% probability level.

2.2.2 Figure S2.2.2; H_2**11**·CH_2Cl_2 (5,15-diphenylporphyrin) DCM

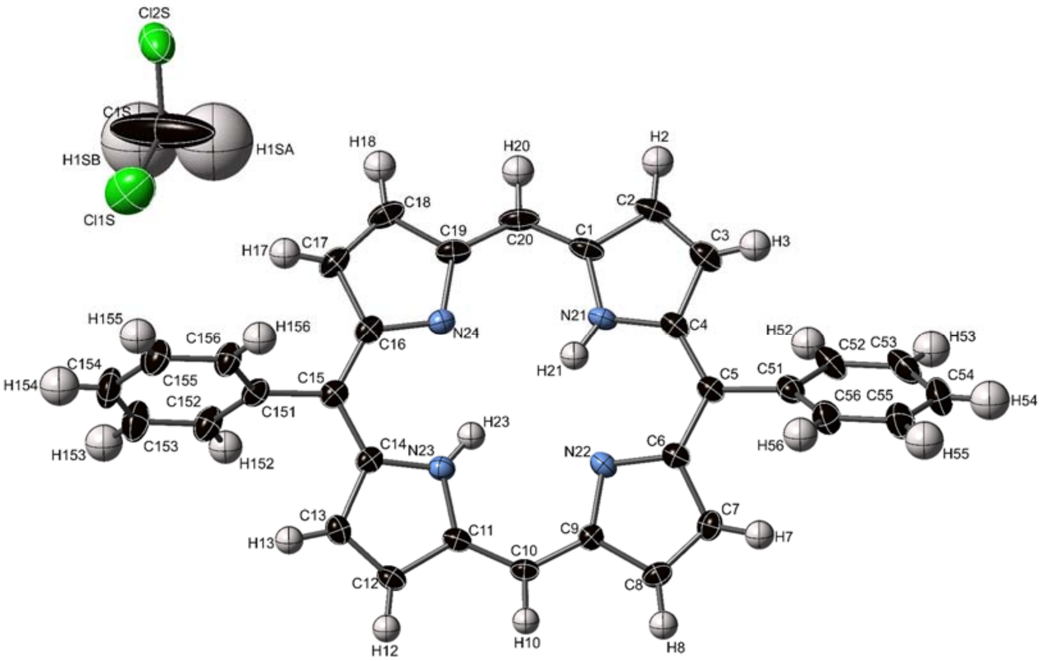

c) A labelled plot of the atoms within the asymmetric unit of compound H_2**11**·CH_2Cl_2. Thermal ellipsoids are shown at 50% probability level.

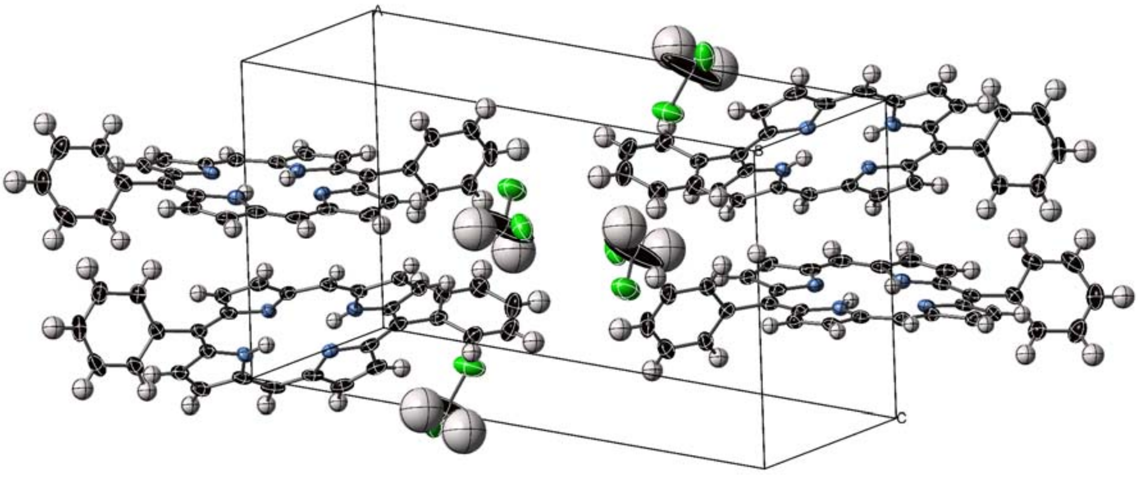

d) A plot of the molecules which comprise the unit cell of the crystal structure of compound H_2**11**·CH_2Cl_2 (Z = 4). Thermal ellipsoids are shown at 50% probability level.

2.2.3 Figure S2.2.3; $[H_4\textbf{11}][CF_3CO_2]_2$ 5,15-diphenylporphyrindi-ium bis(trifluoroacetate)

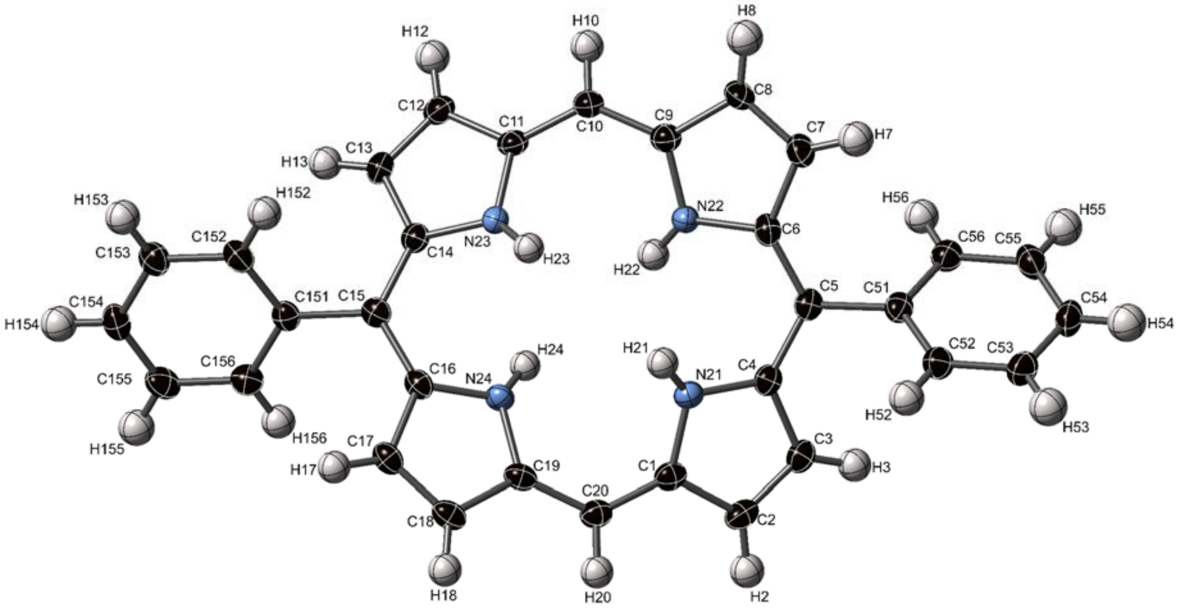

a) A labelled plot of the atoms within the asymmetric unit of compound $[H_4\textbf{11}][CF_3CO_2]_2$. Thermal ellipsoids are shown at 50% probability level; trifluoroacetate anions have been omitted.

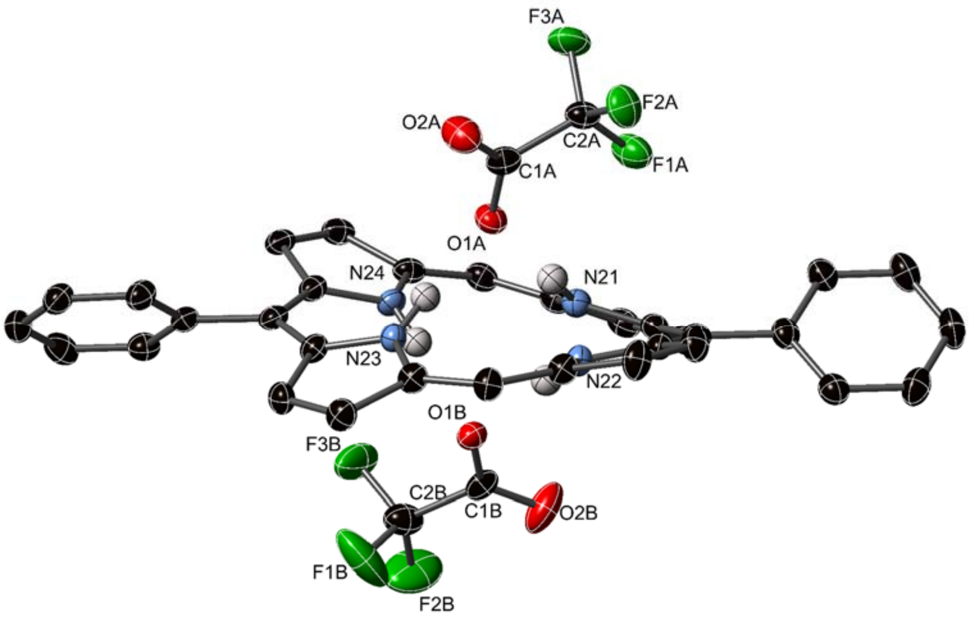

b) A labelled plot of the anions within the asymmetric unit of compound $[H_4\textbf{11}][CF_3CO_2]_2$ and the interaction with the porphyrin core. Thermal ellipsoids are shown at 50% probability level.

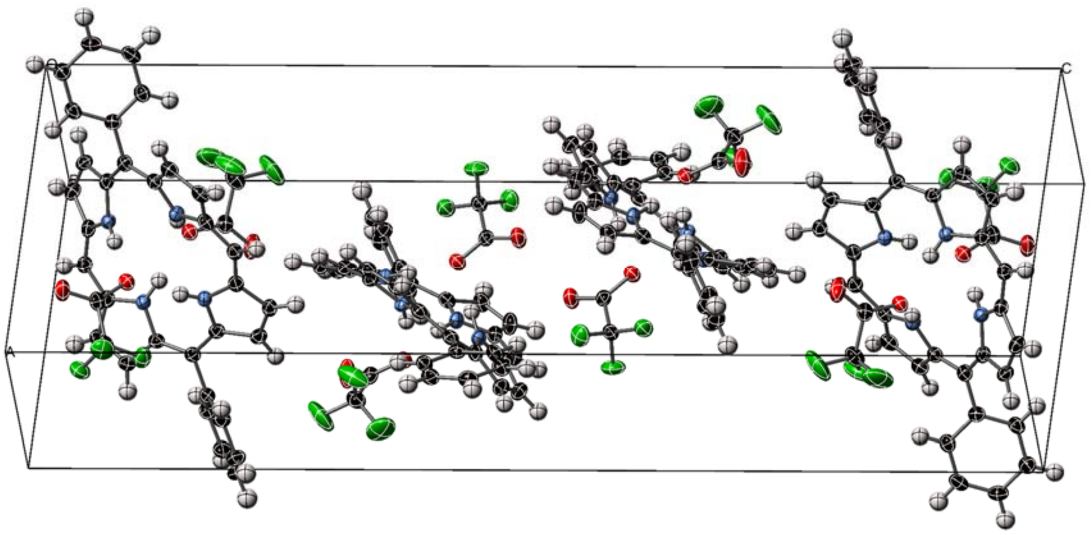

c) The molecules within the unit cell of compound $[H_4\mathbf{11}][CF_3CO_2]_2$ (Z = 4). Thermal ellipsoids are shown at 50% probability level.

2.2.4 Figure S2.2.4; $[H_4\mathbf{11}][CF_3CO_2]_2 \cdot 2CF_3CO_2H$ 5,15-diphenylporphyrindi-ium bis(trifluoroacetate)bis(trifluoroacetic acid solvate)

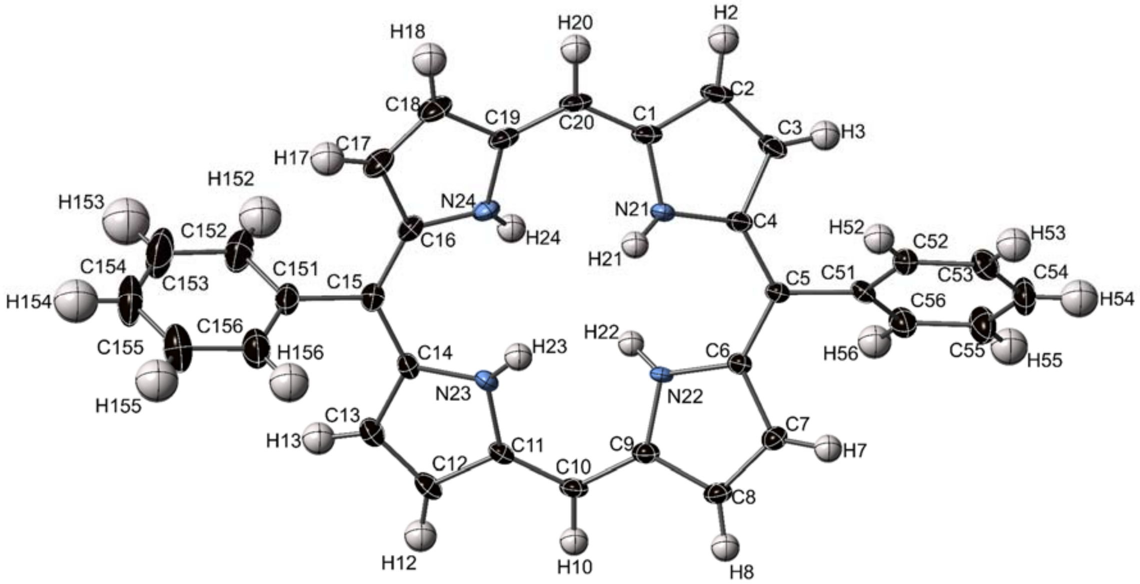

a) A labelled plot of the atoms within the asymmetric unit of compound $[H_4\mathbf{11}][CF_3CO_2]_2 \cdot 2CF_3CO_2H$. Thermal ellipsoids are shown at 50% probability level; trifluoroacetate anions have been omitted.

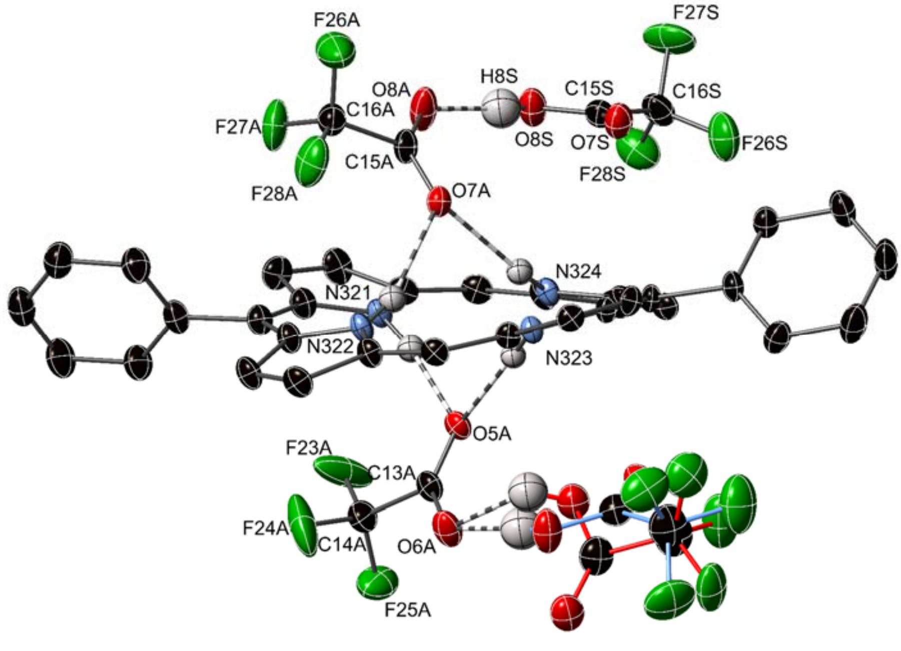

b) Labelled plot of trifluoroacetate anions and trifluoroacetic acid solvate molecules within the asymmetric unit of Compound $[H_4\mathbf{11}][CF_3CO_2]_2 \cdot 2CF_3CO_2H$ and the interaction with the porphyrin core. Thermal ellipsoids are shown at 50% probability level.

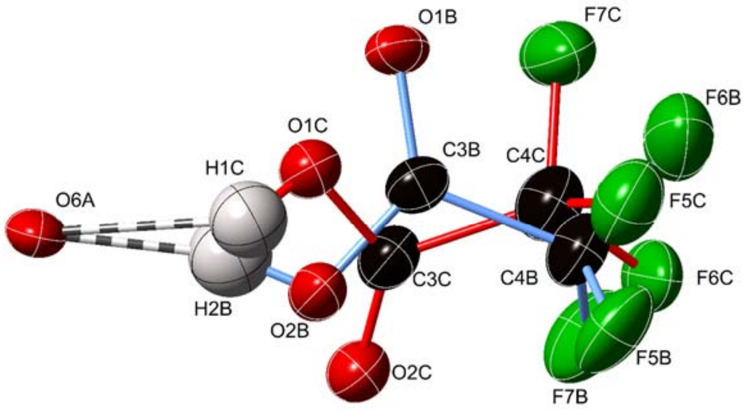

c) A labelled plot of the disordered trifluoroacetic acid solvate component of compound $[H_4$**11**$][CF_3CO_2]_2{\cdot}2CF_3CO_2H$ and the interaction with the porphyrin core. Thermal ellipsoids are shown at 50% probability level. This trifluoroacetic acid molecule was modelled as disordered over two orientations (O1B -F7B (77%) with blue bonds and O1C-F7C (23%) with red bonds). Thermal ellipsoids were constrained with SIMU commands as indicated in Section 1.1.4.

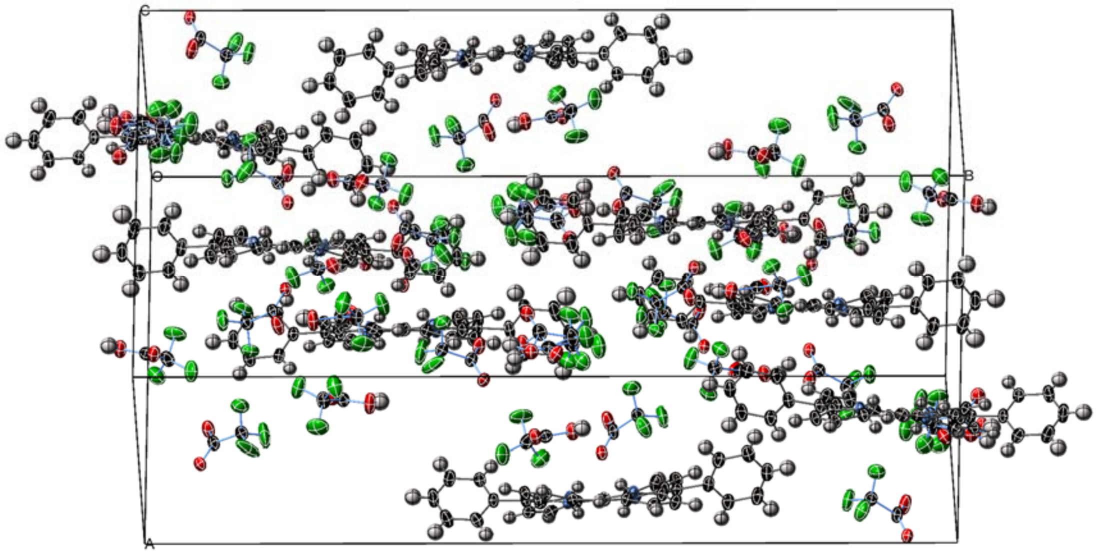

d) The molecules within the unit cell of compound $[H_4$**11**$][CF_3CO_2]_2{\cdot}2CF_3CO_2H$ (Z = 8). Thermal ellipsoids are shown at 50% probability level. Trifluoroacetate anions and trifluoroacetic acid molecules are highlighted with blue bonds.

2.2.5 Figure S2.2.5; H_2**13**·⅓(CH_2Cl_2) 5,15-bis(3-pentyl)porphyrin 1/3(DCM)

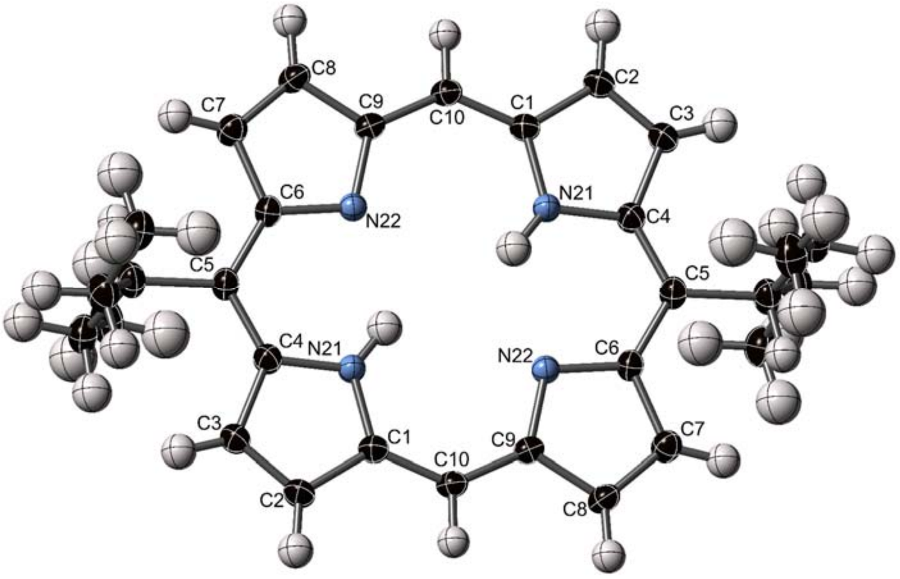

a) A labelled plot of the atoms within the porphyrin core of compound H_2**13**·⅓(CH_2Cl_2). Thermal ellipsoids are shown at 50% probability level; DCM solvate has been omitted. This molecule lies on a centre of inversion symmetry and therefore includes two asymmetric units.

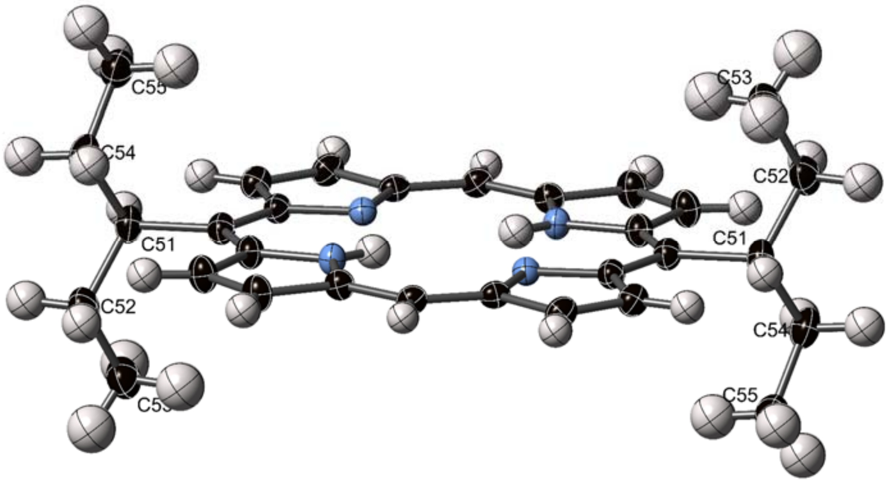

b) Labelled plot of the side chains of the porphyrin within the molecular unit of compound H_2**13**·⅓(CH_2Cl_2). Thermal ellipsoids are shown at 50% probability level.

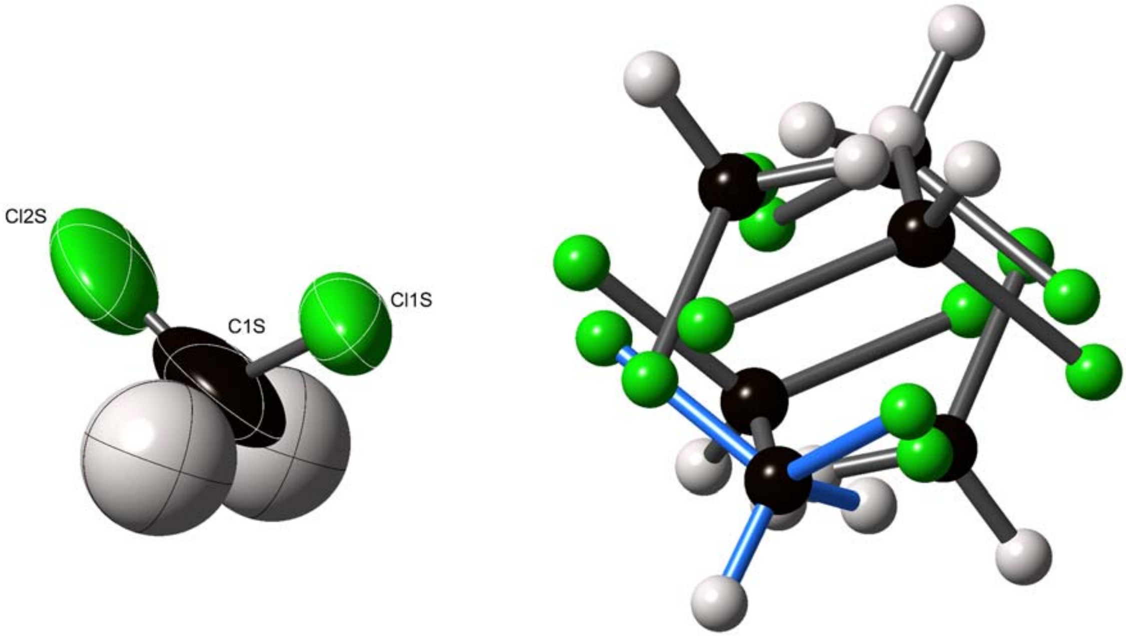

c) Left - A labelled plot of the dichloromethane solvate (thermal ellipsoids 50%) and Right – the six molecules which are disordered over the solvate site, with a single DCM molecule highlighted in blue and atoms represented as spheres. Sum occupancy of all DCM molecules at the site is constrained to 1; SIMU was used to constrain the thermal ellipsoids of the highly disordered molecule.

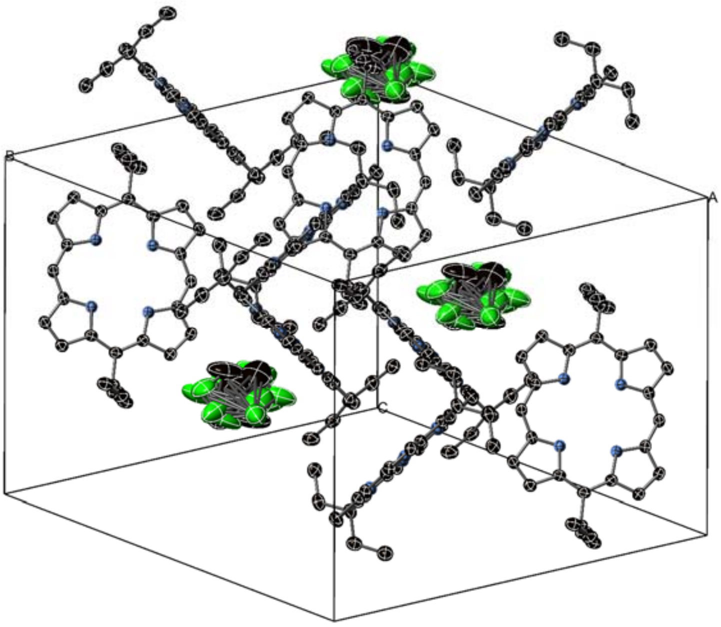

d) The molecules within the unit cell of compound $H_2\mathbf{13}\cdot\frac{1}{3}(CH_2Cl_2)$ (Z = 9). Thermal ellipsoids are shown at 50% probability level. Hydrogen atoms have been omitted.

2.2.6 Figure S2.2.6; [H_4**13**][CF_3CO_2]$_2$ 5,15-bis(4-(3-pentyl))porphyrindi-ium bis(trifluoroacetate)

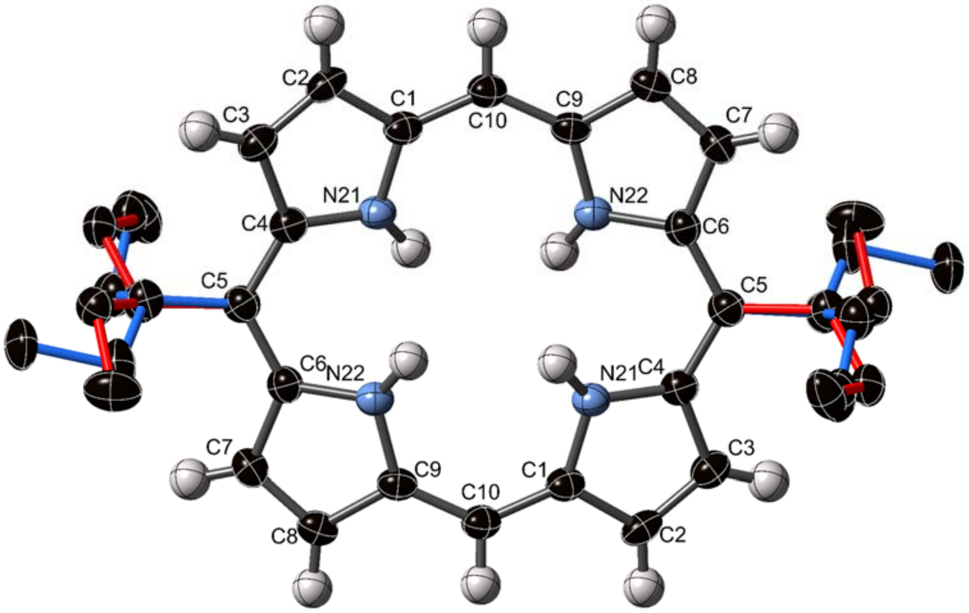

a) A labelled plot of the atoms within the porphyrin core of Compound [H_4**13**][CF_3CO_2]$_2$. Thermal ellipsoids are shown at 50% probability level. This molecule lies on a centre of inversion symmetry and therefore includes two asymmetric units.

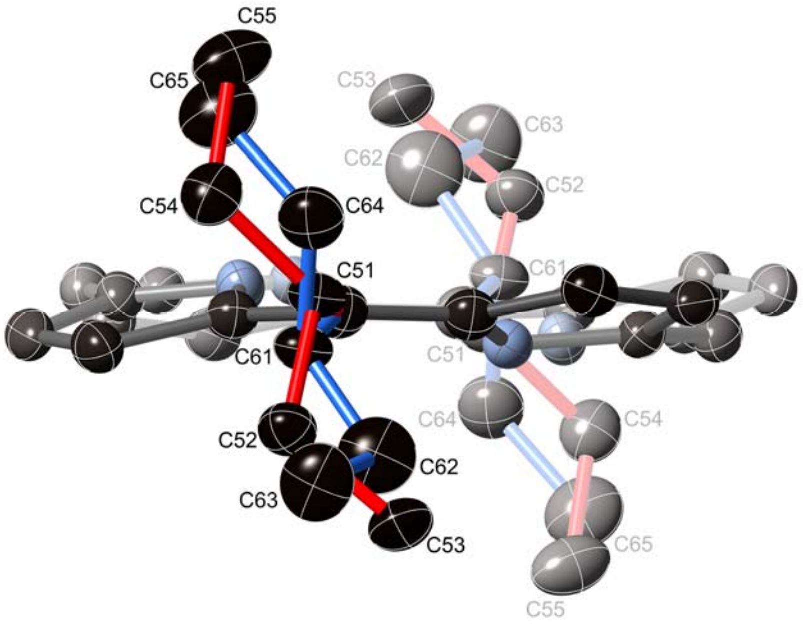

b) Labelled plot of the side chains of the porphyrin within the molecular unit of compound [H_4**13**][CF_3CO_2]$_2$. Thermal ellipsoids are shown at 50% probability level.

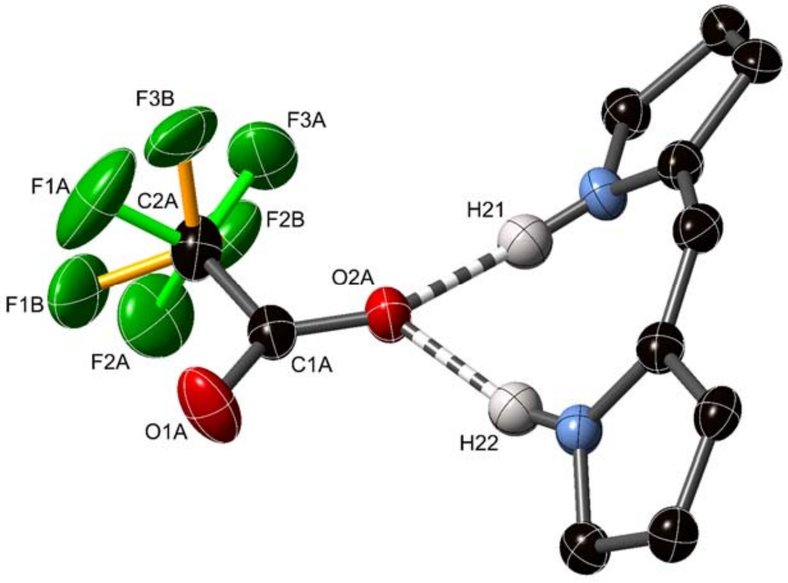

c) Left - A labelled plot of the disordered trifluoroacetate anion (thermal ellipsoids 50%) and the interaction with two adjacent pyrrole units of the porphyrin core; the three F atoms are disordered over two orientations.

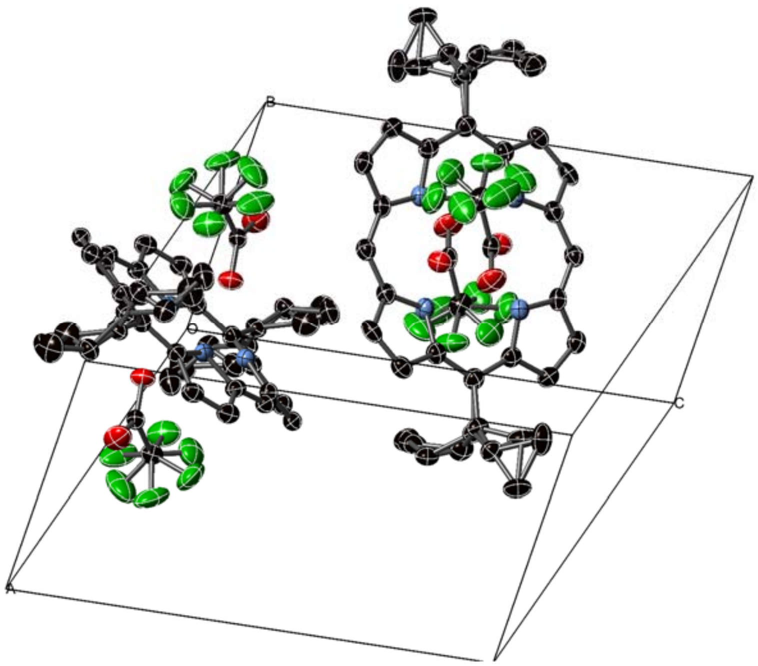

d) The molecules within the unit cell of compound $[H_4\mathbf{13}][CF_3CO_2]_2$ (Z = 2). Thermal ellipsoids are shown at 50% probability level. Hydrogen atoms have been omitted.

2.2.7 Figure S2.2.7; $[H_4\mathbf{14}][CF_3CO_2]_2$ 5,10,15-triphenylporphyrindi-ium bis(trifluoroacetate)

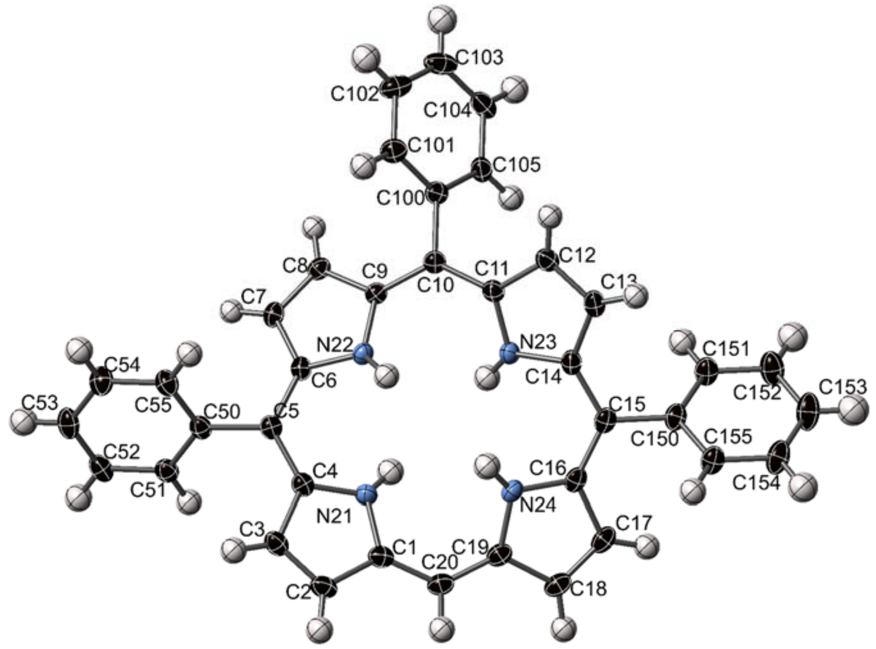

a) A labelled plot of the atoms within the porphyrin component of compound $[H_4\mathbf{14}][CF_3CO_2]_2$, 5,10,15-triphenylporphyrindi-ium bis(trifluoroacetate). Thermal ellipsoids are shown at 50% probability level; trifluoroacetate anions have been omitted.

b) Labelled plot of the hydrogen-bonded trifluoroacetate anions coordinated to the porphyrin

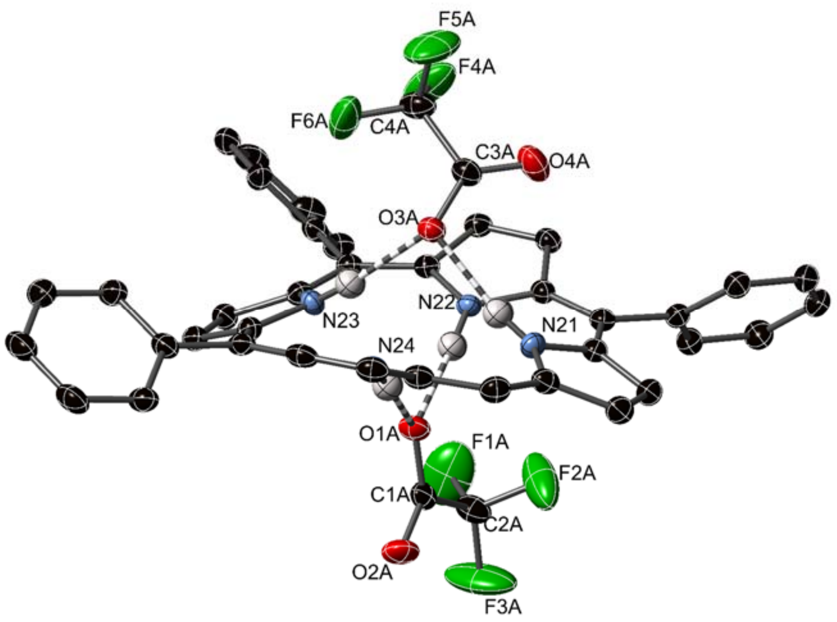

core within the molecular unit of compound $[H_4\mathbf{14}][CF_3CO_2]_2$. Thermal ellipsoids are shown at 50% probability level.

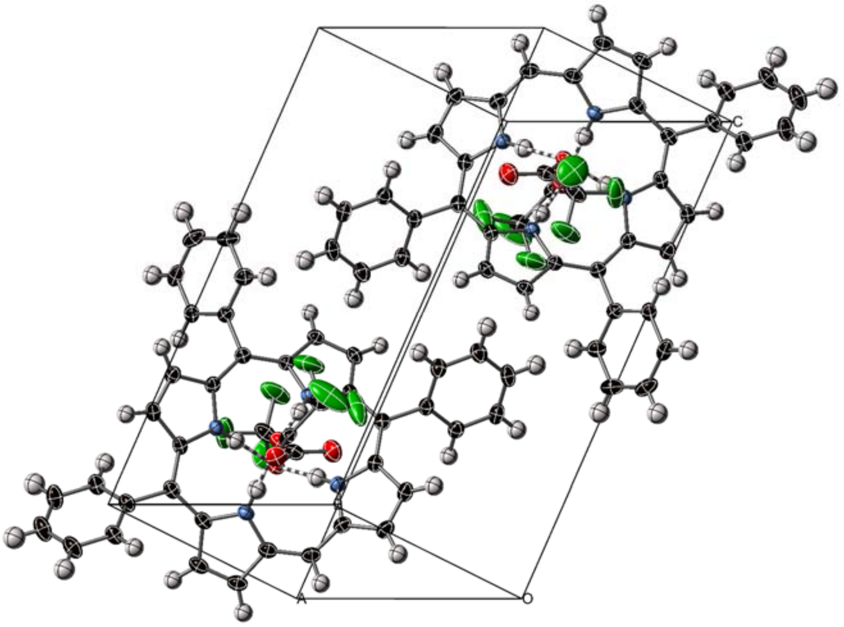

c) The molecules within the unit cell of compound [H_4**14**][CF_3CO_2]$_2$ (Z = 2). Thermal ellipsoids are shown at 50% probability level.

2.2.8 Figure S2.2.8; H_2**15** 5,15-bis(4-butoxyphenyl)porphyrin

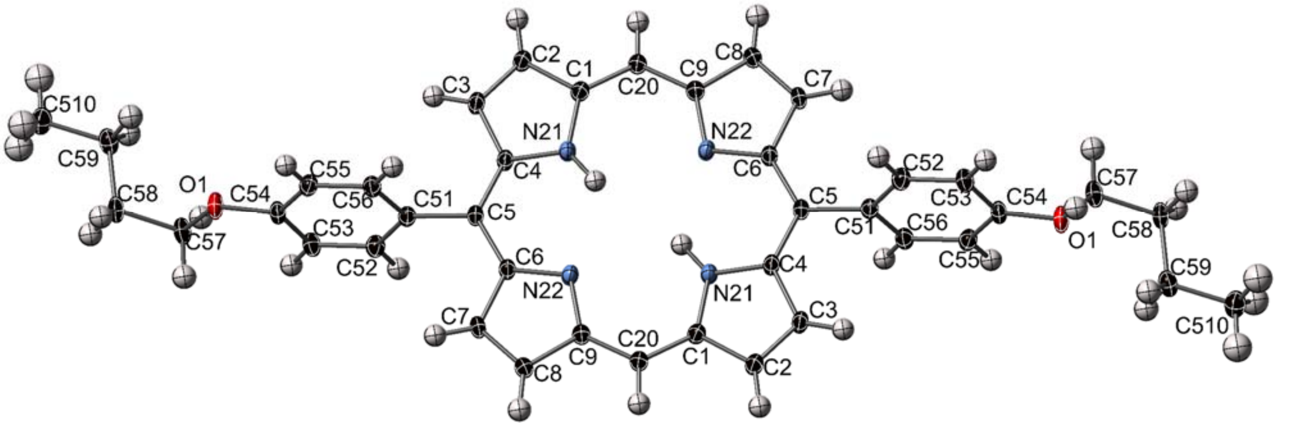

a) A labelled plot of the atoms within the asymmetric unit of compound H_2**15**, 5,15-bis(4-butoxyphenyl)porphyrin. Thermal ellipsoids are shown at 50% probability level; DCM solvate has been omitted. This molecule lies on a centre of inversion symmetry and therefore includes two asymmetric units.

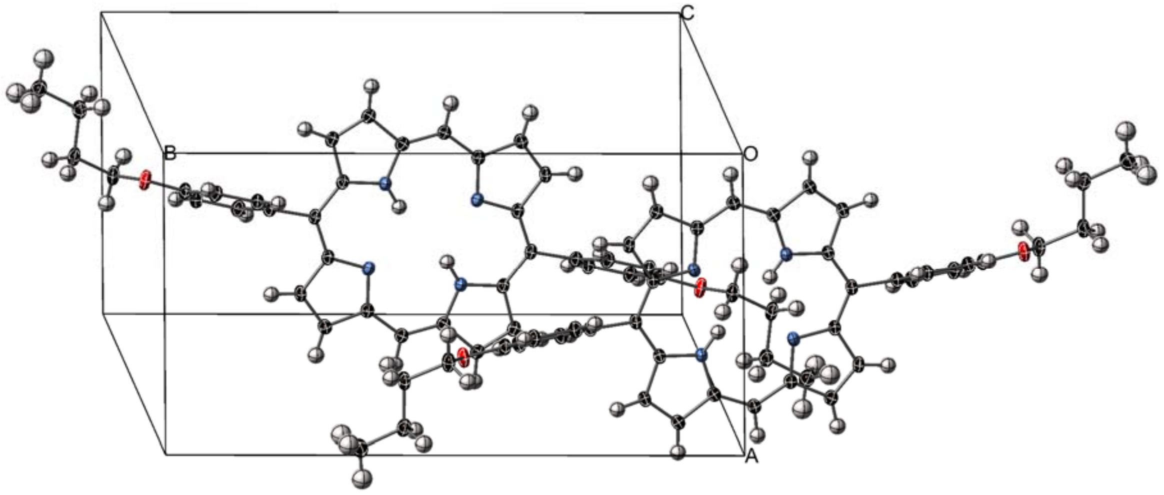

b) The molecules within the unit cell of compound H_2**15** (Z = 2). Thermal ellipsoids are shown at 50% probability level.

2.2.9 Figure S2.2.9; [H_4**17**][CF_3CO_2]$_2$·2CF_3CO_2H 5,15-bis(4-methoxyphenyl)porphyrindi-ium bis(trifluoroacetate) bis(trifluoroacetic acid solvate)

a) A labelled plot of the atoms within the porphyrin component of the asymmetric unit of

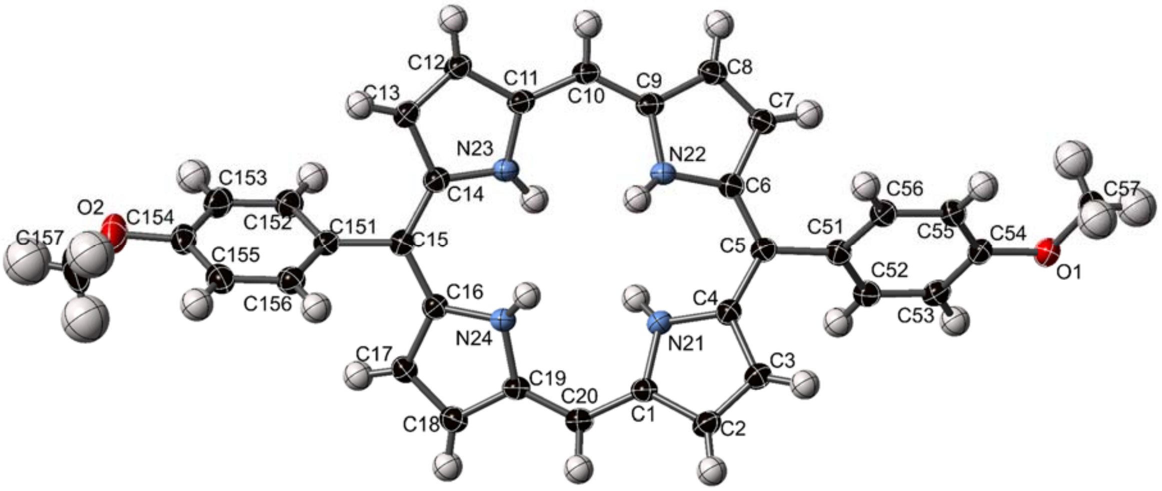

Compound [H_4**17**][CF_3CO_2]$_2$·2CF_3CO_2H, 5,15-bis(4-methoxyphenyl)porphyrindi-ium bis(trifluoroacetate) bis(trifluoroacetic acid solvate). Thermal ellipsoids are shown at 50% probability level.

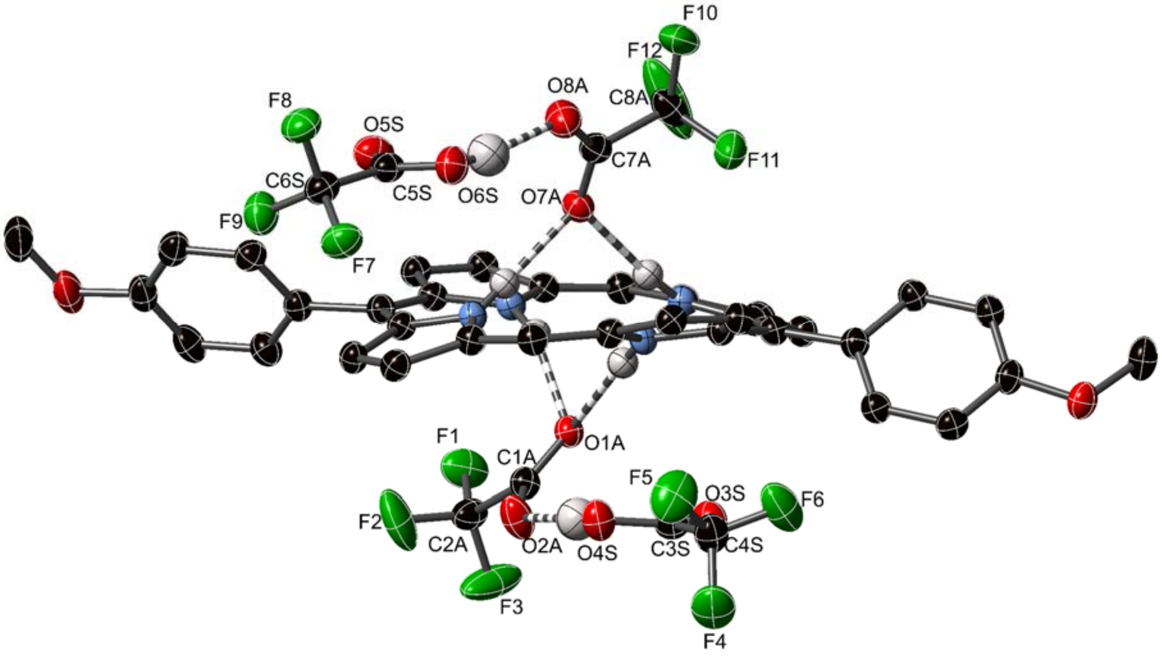

b) Labelled plot of trifluoroacetate anions and trifluoroacetic acid solvate molecules within the asymmetric unit of compound [H_4**17**][CF_3CO_2]$_2$·2CF_3CO_2H and the interaction with the porphyrin core. Thermal ellipsoids are shown at 50% probability level.

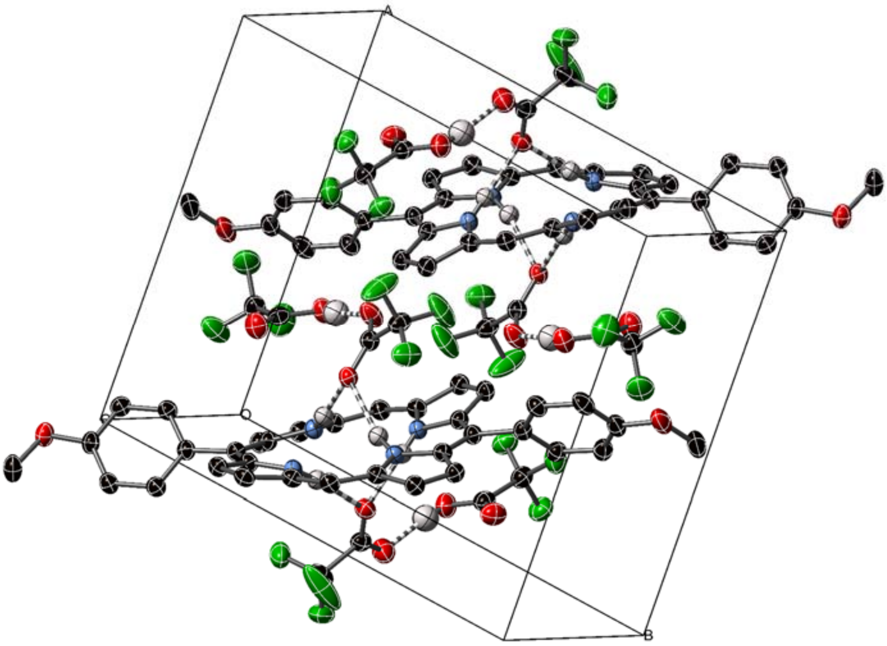

c) The molecules within the unit cell of Compound [H_4**17**][CF_3CO_2]$_2$·2CF_3CO_2H (Z = 2). Thermal ellipsoids are shown at 50% probability level. C-bound hydrogen atoms have been omitted.

2.2.10 Figure S2.2.10; [H_4**18**][ClO_4]$_2$ 5,15-bis(4-bromophenyl)porphyrindi-ium bis(perchlorate)

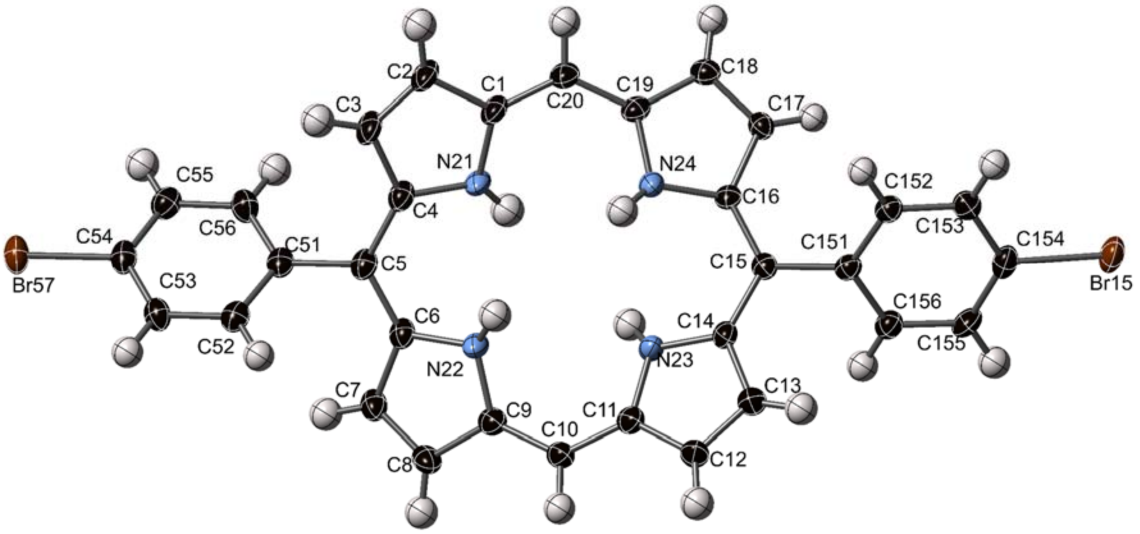

a) A labelled plot of the atoms within the porphyrin component of the asymmetric unit of Compound [H_4**18**][ClO_4]$_2$, 5,15-bis(4-bromophenyl)porphyrindi-ium bis(perchlorate). Thermal ellipsoids are shown at 50% probability level.

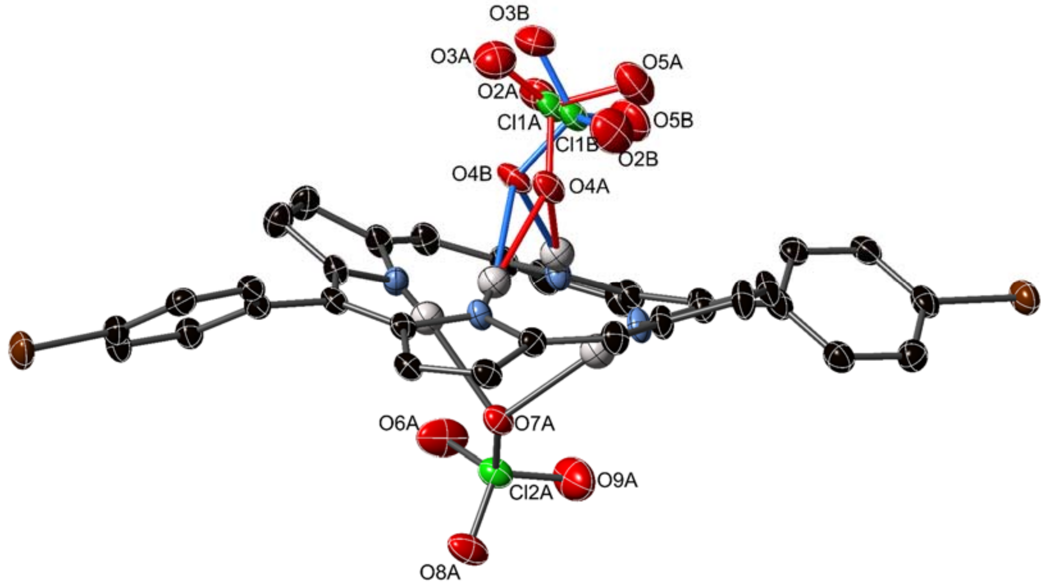

b) Labelled plot of perchlorate anions within the asymmetric unit of compound [H_4**18**][ClO_4]$_2$ and the interaction with the porphyrin core. Thermal ellipsoids are shown at 50% probability level. H-atoms not involved in hydrogen bonding have been omitted, The molecule Cl1/O1-O4 is disordered over two orientations (A and B) as indicated by red bonds (A, 20%) and blue bonds (B, 80%)

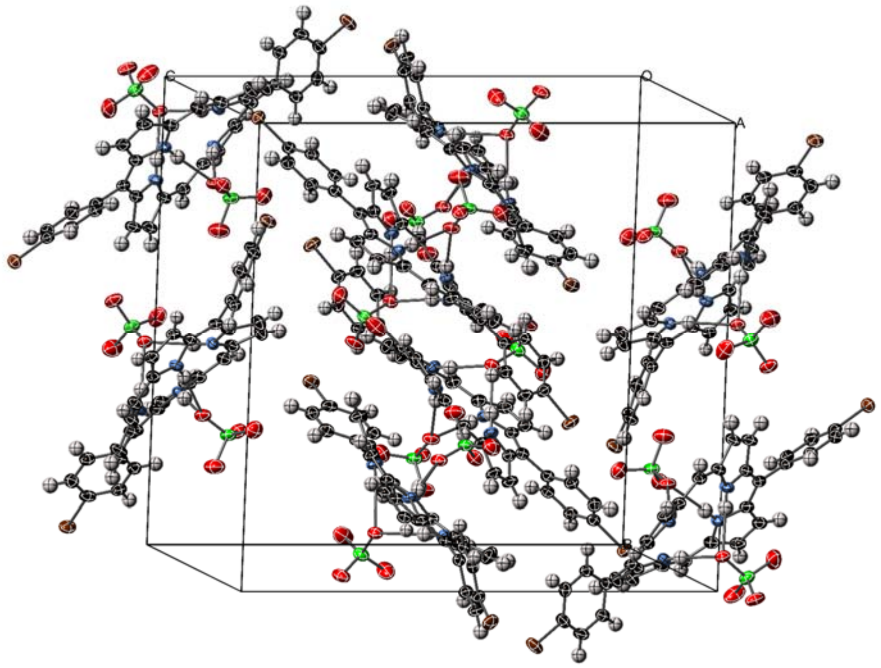

c) The molecules within the unit cell of compound [H_4**18**][ClO_4]$_2$ (Z = 8). Thermal ellipsoids are shown at 50% probability level.

2.2.11 Figure S2.2.11; $[H_4\mathbf{18}][CF_3CO_2]_2{\cdot}2CF_3CO_2H$ 5,15-bis(4-bromophenyl)porphyrindi-ium bis(trifluoroacetate) bis(trifluoroacetic acid solvate)

a) A labelled plot of the atoms within the porphyrin component of the asymmetric unit of

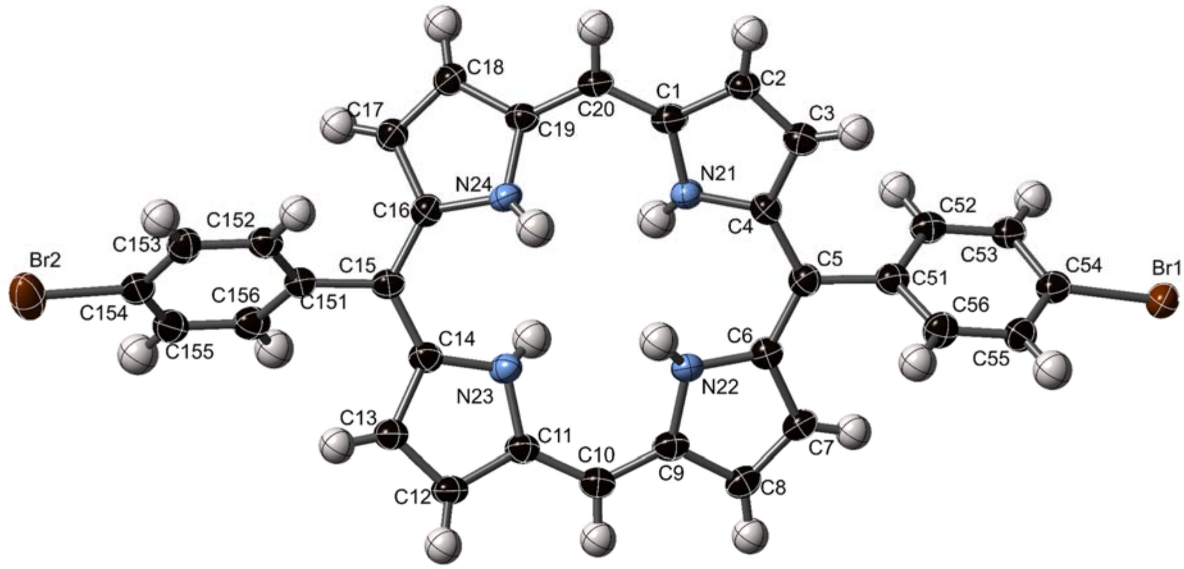

Compound $[H_4\mathbf{18}][CF_3CO_2]_2{\cdot}2CF_3CO_2H$, 5,15-bis(4-bromophenyl)porphyrindi-ium bis(trifluoroacetate) bis(trifluoroacetic acid solvate). Thermal ellipsoids are shown at 50%

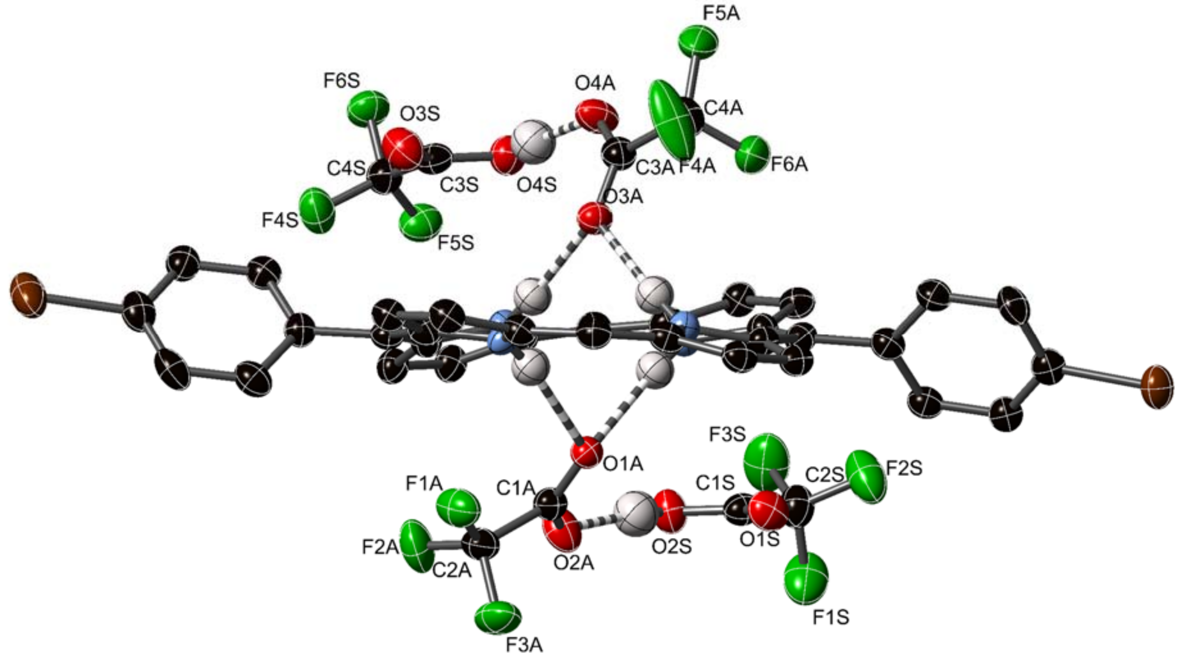

probability level.

b) Labelled plot of trifluoroacetate anions and trifluoroacetic acid solvate within the asymmetric unit of compound $[H_4\mathbf{18}][CF_3CO_2]_2{\cdot}2CF_3CO_2H$, and the interaction with the porphyrin core. Thermal ellipsoids are shown at 50% probability level. H-atoms not involved in hydrogen bonding have been omitted.

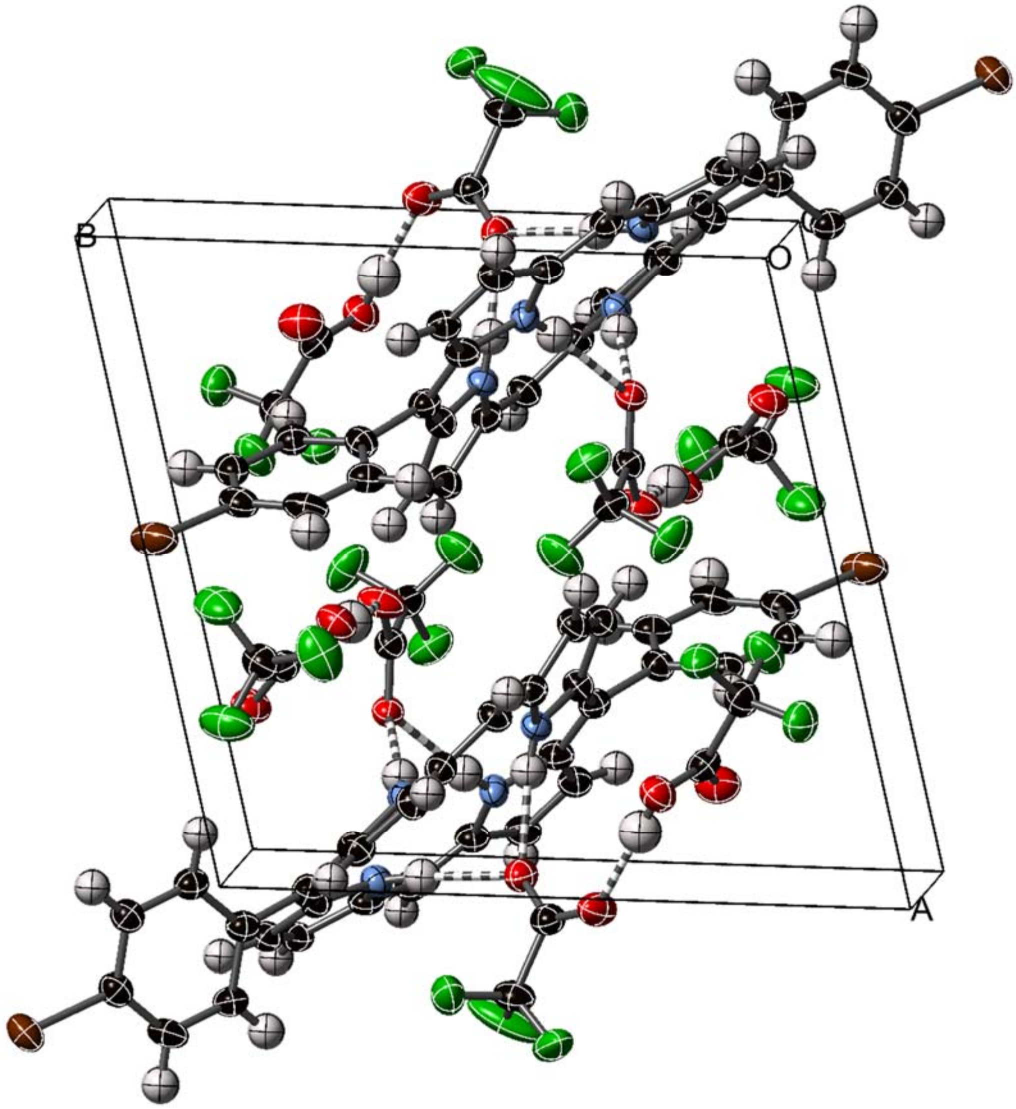

c) The molecules within the unit cell of compound [H_4**18**][CF_3CO_2]$_2$·2CF_3CO_2H (Z = 2). Thermal ellipsoids are shown at 50% probability level.

2.2.12 Figure S2.2.12; $[H_4\mathbf{19}][CF_3CO_2]_2\cdot 2CF_3CO_2H$ 5,15-bis(4-methylthiophenyl)porphyrindi-ium bis(trifluoroacetate) bis(trifluoroacetic acid) solvate

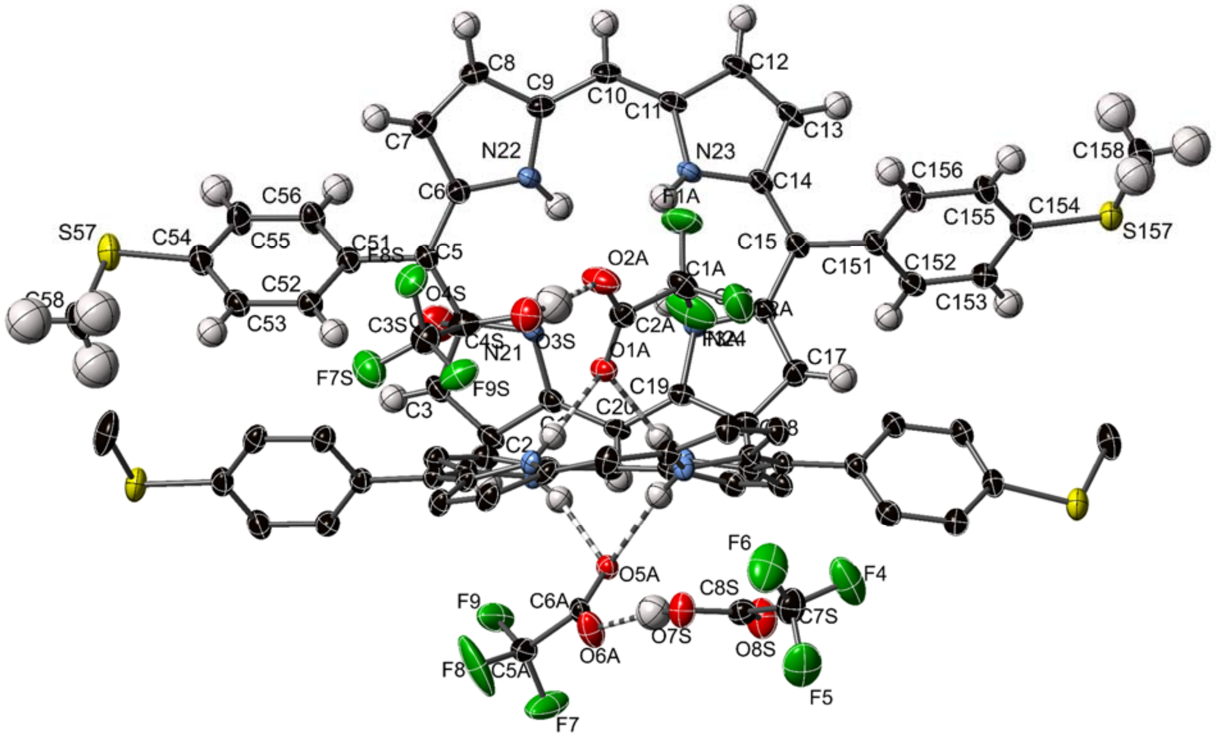

a) A labelled plot of the atoms within the porphyrin component of the asymmetric unit of compound $[H_4\mathbf{19}][CF_3CO_2]_2\cdot 2CF_3CO_2H$, 5,15-bis(4-methylthiophenyl)porphyrindi-ium bis(trifluoroacetate) bis(trifluoroacetic acid) solvate. Thermal ellipsoids are shown at 50% probability level.
b) Labelled plot of trifluoroacetate anions and trifluoroacetic acid solvate within the asymmetric unit of Compound $[H_4\mathbf{19}][CF_3CO_2]_2\cdot 2CF_3CO_2H$, and the interaction with the porphyrin core. Thermal ellipsoids are shown at 50% probability level. H-atoms not involved in hydrogen bonding have been omitted.

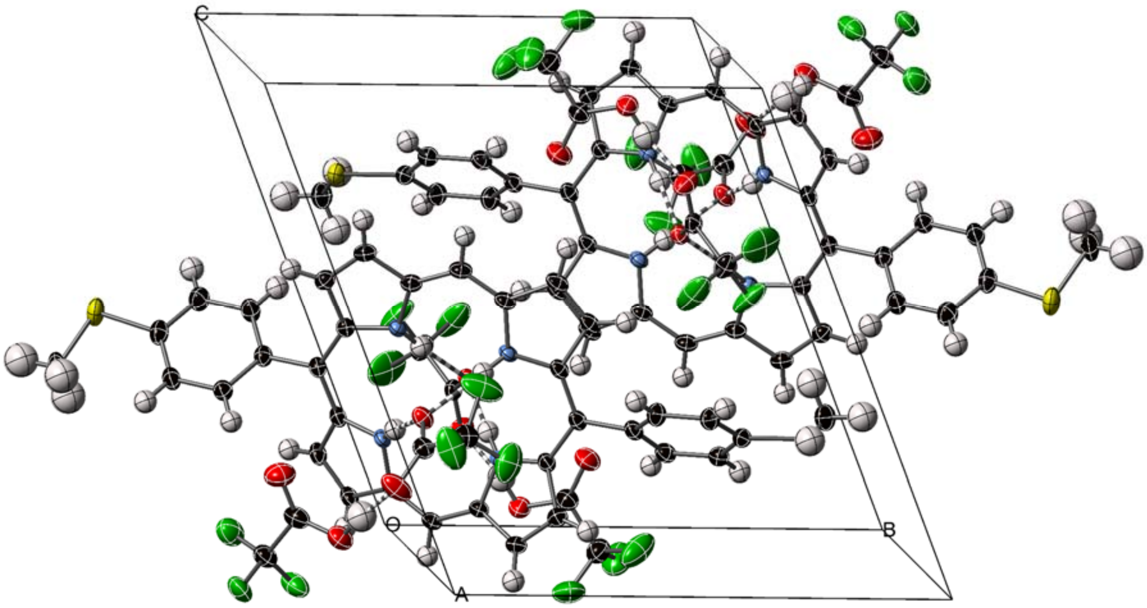

c) The molecules within the unit cell of compound [H_4**19**][CF_3CO_2]$_2$·2CF_3CO_2H (Z = 2). Thermal ellipsoids are shown at 50% probability level.

2.2.13 Figure S2.2.13; H_2**19** 5,15-diphenyl-10-(thiophen-3-yl)porphyrin

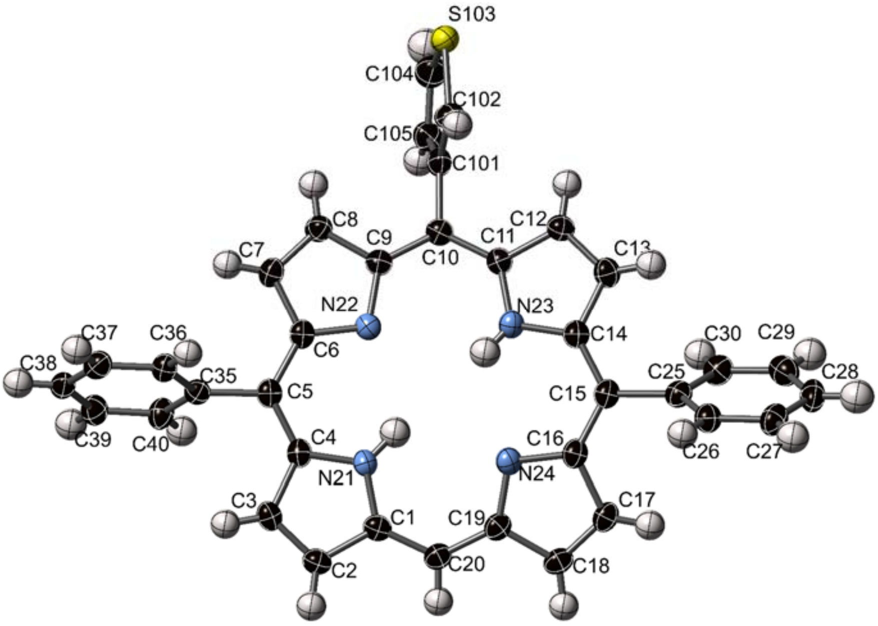

a) A labelled plot of the atoms in the asymmetric unit of compound H_2**19**, 5,15-diphenyl-10-(thiophen-3-yl)porphyrin. Thermal ellipsoids are shown at 50% probability level. Disorder of the thiophene component has been omitted from this view, and only the primary component (C101-C102-S103-C104-C105, 62%) is shown.

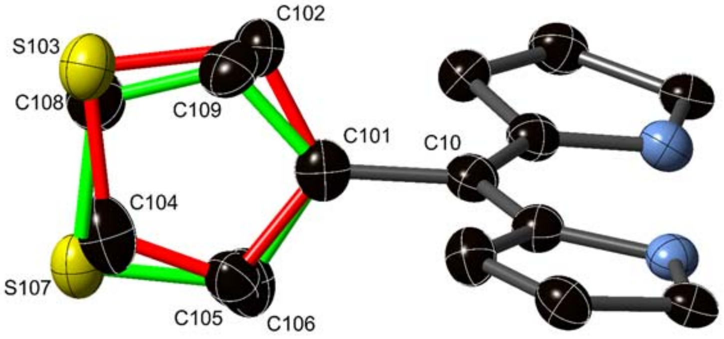

b) A view of the two-component disorder model of the thiophene at the 10-position of the porphyrin ring. These two orientations are shown in red bonds (C101-C102-S103-C104-C105, 62%) and green bonds (C101-C106-S107-C108-C109, 38%), representing approx. 180° rotation of the thiophene around the thiophene-porphyrin bond. These atoms were constrained by SADI and SIMU commands. H-atoms are omitted.

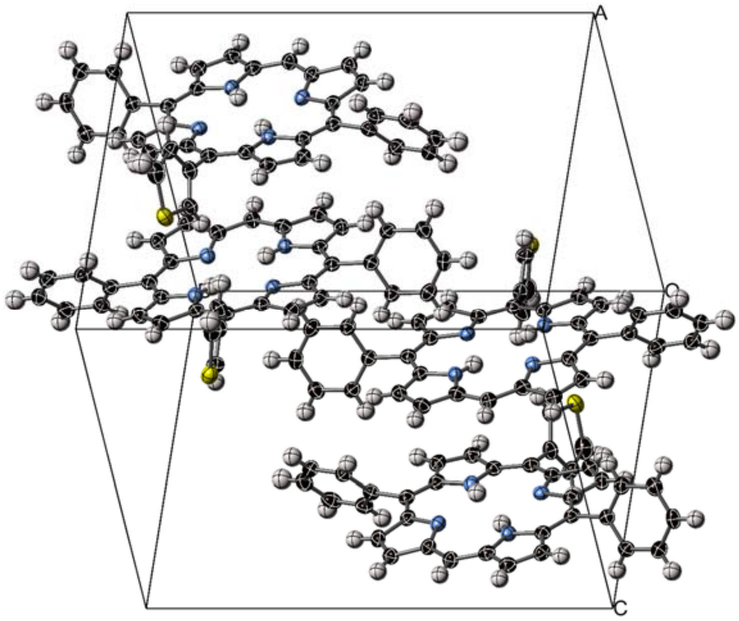

c) The molecules within the unit cell of compound H_2**19** (Z = 2). Thermal ellipsoids are shown at 50% probability level. Disorder of the thiophene component has been omitted from this view, and only the primary component (C101-C102-S103-C104-C105, 62%) is shown.

2.2.14 Figure S2.2.14; $[H_4\mathbf{20}][ClO_4]_2$ 5-bromo-10,20-diphenylporphyrindi-ium diperchlorate

a) A labelled plot of the porphyrin component of the asymmetric unit of compound

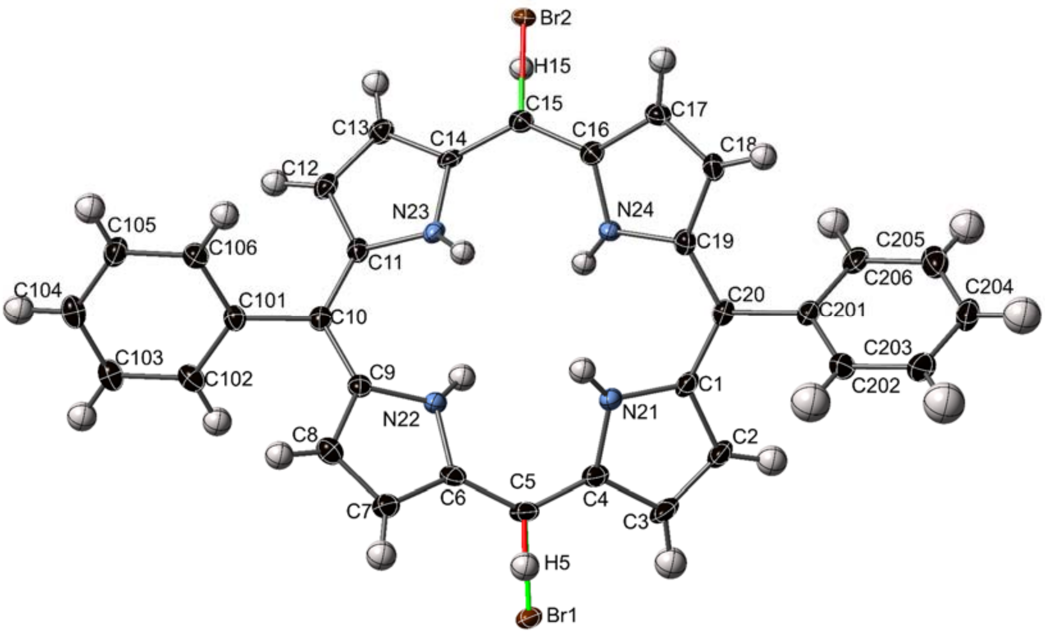

$[H_4\mathbf{20}][ClO_4]_2$, 5-bromo-10,20-diphenylporphyrindi-ium diperchlorate. Thermal ellipsoids are shown at 50% probability level. Disorder of the bromo component (Br1, 91% and Br2, 28%) is shown in red and green bonds, however a significant component of this crystal could only be accounted for by an assumed impurity of 5,15-dibromo-10,20-diphenylporphyrindi-ium diperchlorate.

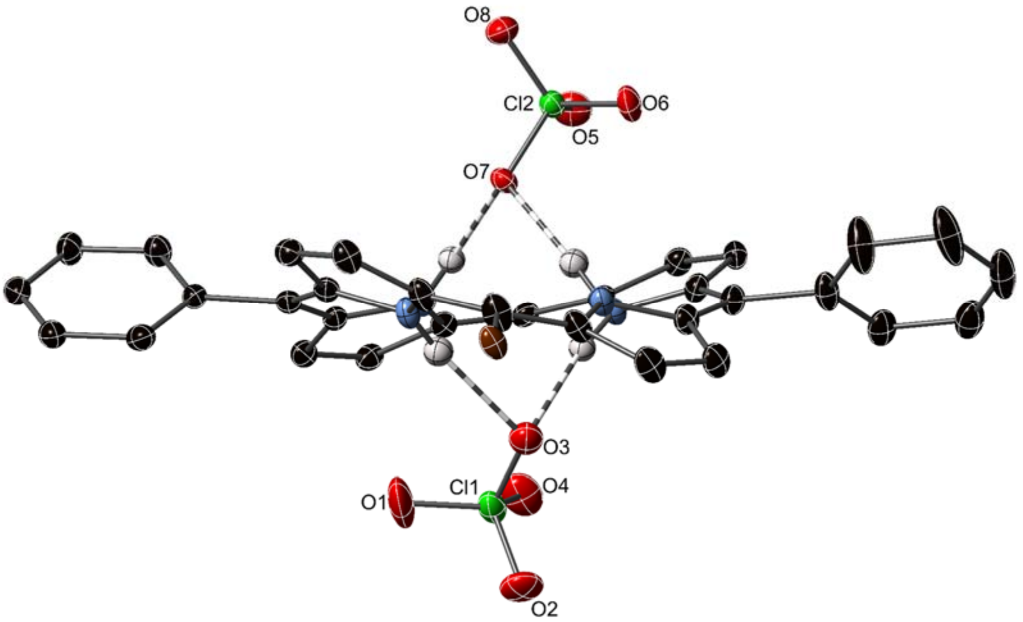

b) A labelled plot of perchlorate anions in the asymmetric unit of compound $[H_4\mathbf{20}][ClO_4]_2$. Thermal ellipsoids are shown at 50% probability level, and H-atoms not involved in hydrogen bonding are omitted. Disordered H- and Br atoms are omitted from this view, with only the principal molecular component (Br1, 91% and H15, 72%) shown.

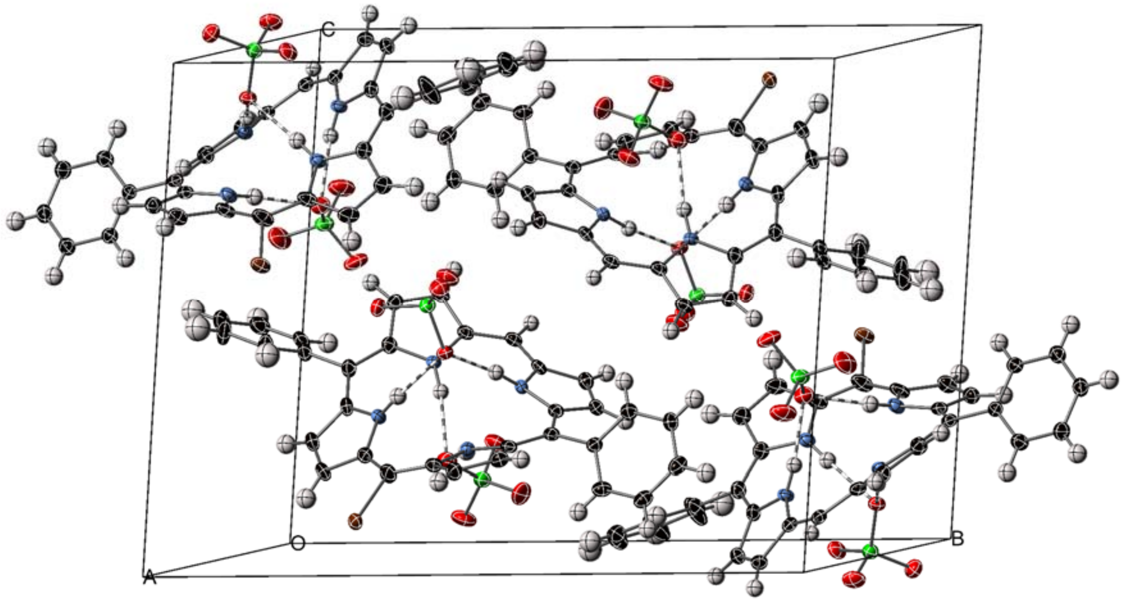

c) The molecules within the unit cell of compound $[H_4\mathbf{20}][ClO_4]_2$ (Z = 4). Thermal ellipsoids are shown at 50% probability level. Disordered H- and Br atoms are omitted from this view, with only the principal molecular component (Br1, 91% and H15, 72%) shown.

2.2.15 Figure S2.2.15; $[H_4\textbf{21}][CF_3CO_2]_2{\cdot}2CF_3CO_2H$ 5,15-dibromo-10,20-bis(4-tolyl)porphyrindi-ium bis(trifluoroacetate) bis(trifluoroacetic acid) solvate

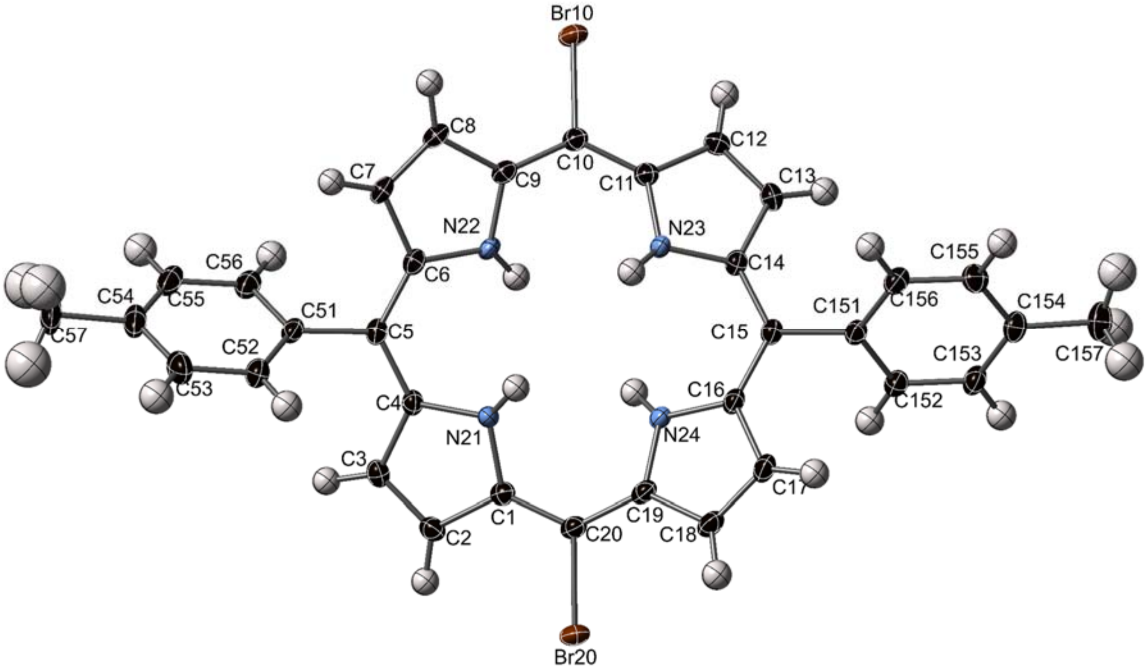

a) A labelled plot of the porphyrin component of the asymmetric unit of compound $[H_4\textbf{21}][CF_3CO_2]_2{\cdot}2CF_3CO_2H$, 5-bromo-10,20-diphenylporphyrindi-ium diperchlorate. Thermal ellipsoids are shown at 50% probability level.

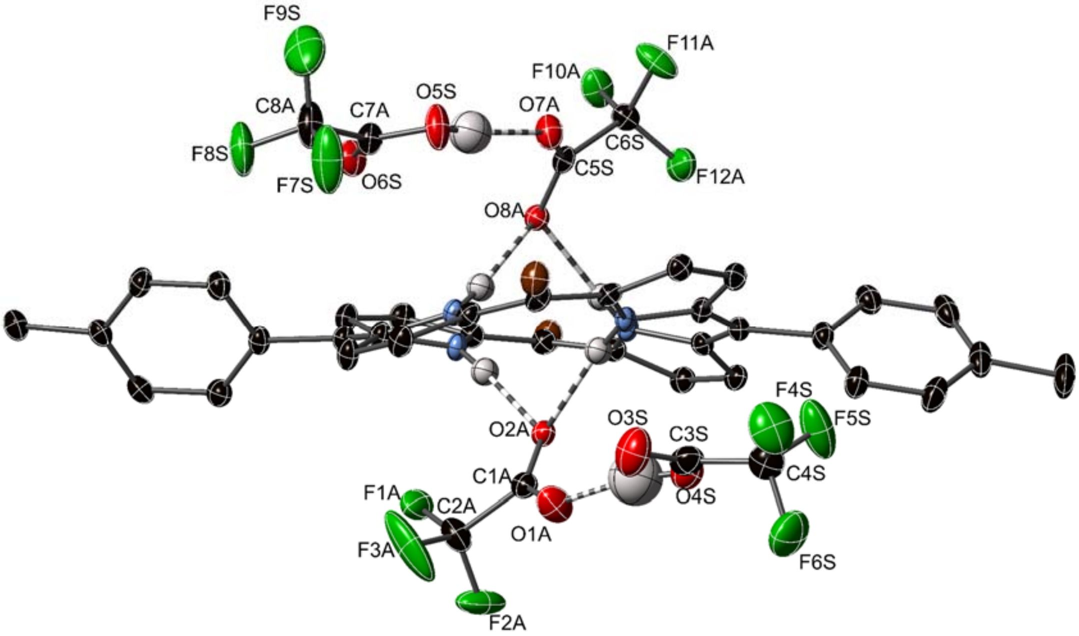

b) A labelled plot of the trifluoroacetate anions and trifluoroacetic acid solvate molecules in the asymmetric unit of compound $[H_4\textbf{21}][CF_3CO_2]_2{\cdot}2CF_3CO_2H$. Thermal ellipsoids are shown at 50% probability level, and H-atoms not involved in hydrogen bonding are omitted from this view.

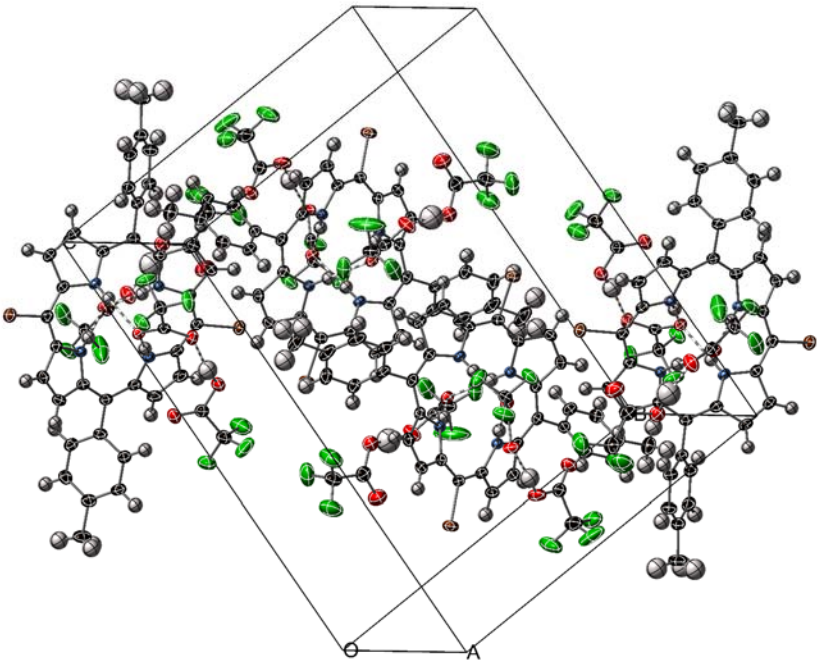

c) The molecules within the unit cell of compound [H_4**21**][CF_3CO_2]$_2$·2CF_3CO_2H (Z = 4). Thermal ellipsoids are shown at 50% probability level.

2.2.16 Figure S2.2.16; $[H_4\mathbf{22}][MeSO_4]_2 \cdot ¼H_2O$ 5,15-bis(4-ethynylphenyl)-10,20-diphenylporphyrindi-ium bis(methylsulfate) hemiaqua solvate

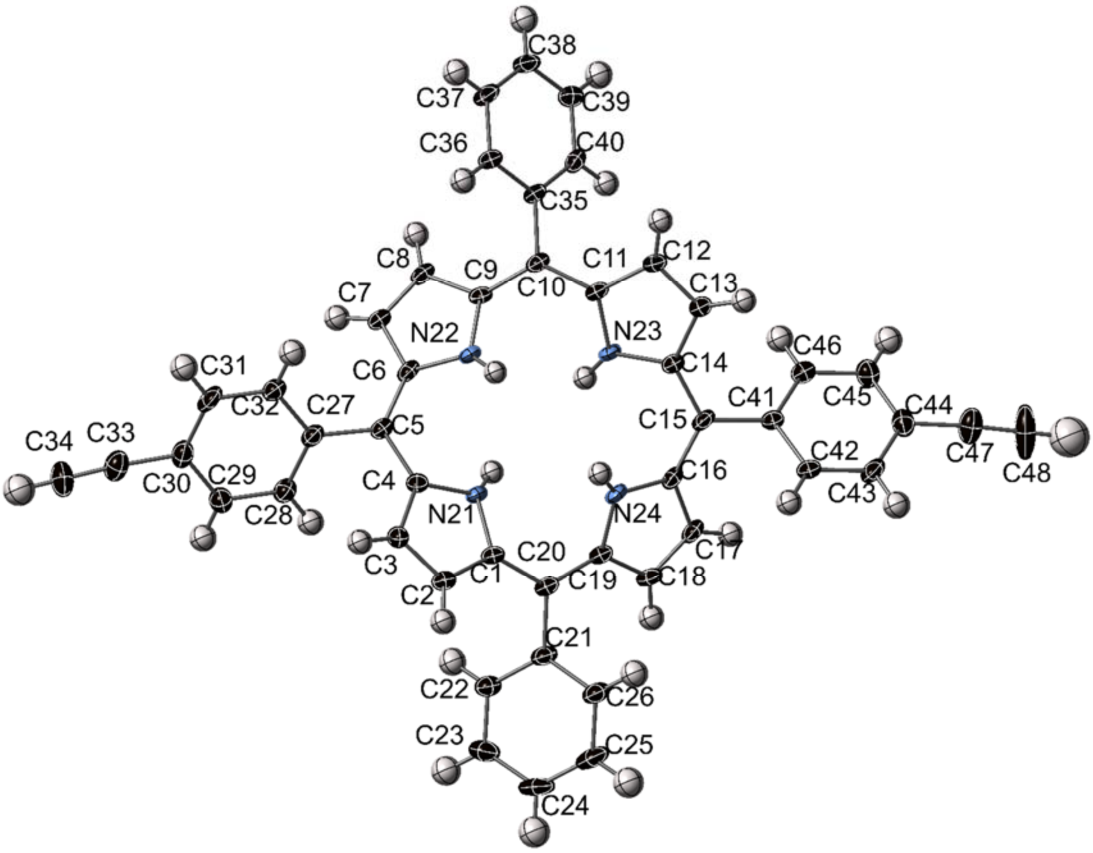

a) A labelled plot of the porphyrin component of the asymmetric unit of compound $[H_4\mathbf{22}][MeSO_4]_2 \cdot ¼H_2O$, 5,15-bis(4-ethynylphenyl)-10,20-diphenylporphyrindi-ium bis(methylsulfate) hemiaqua solvate. Thermal ellipsoids are shown at 50% probability level.

b) A labelled plot of one of the disordered methylsulfate anions. Orientations are shown in

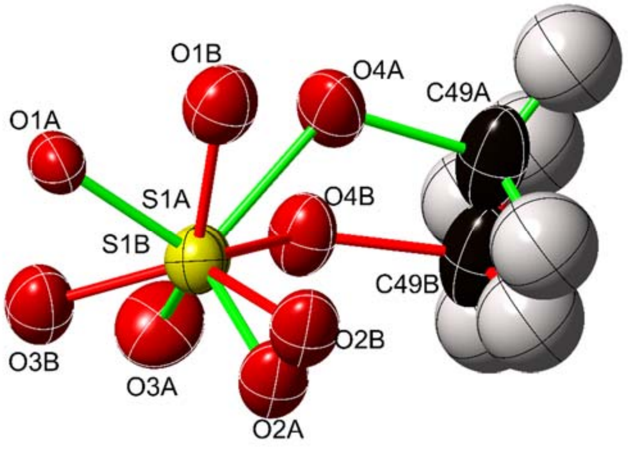

green bonds (S1A, O1A-O4A and C49A, 92%) and red bonds (S1B, O1B-O4B and C49B, 8%)

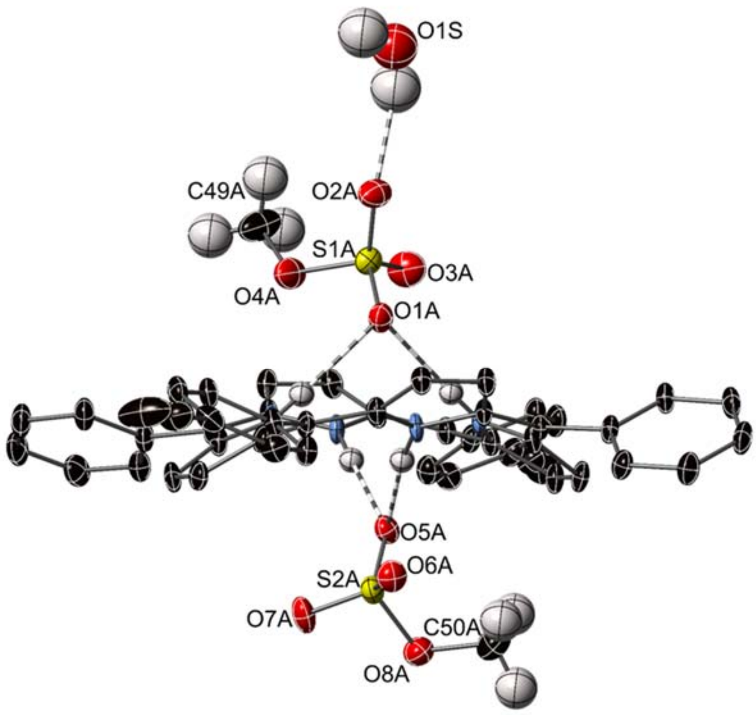

c) A labelled plot of the methylsulfate anions and water solvate molecule in the asymmetric unit of compound [H_4**22**][$MeSO_4$]$_2$·¼H_2O. Thermal ellipsoids are shown at 50% probability level, and H-atoms not involved in hydrogen bonding are omitted from this view. Disorder of the methylsulfate anions is omitted from this view, with only the primary component shown.

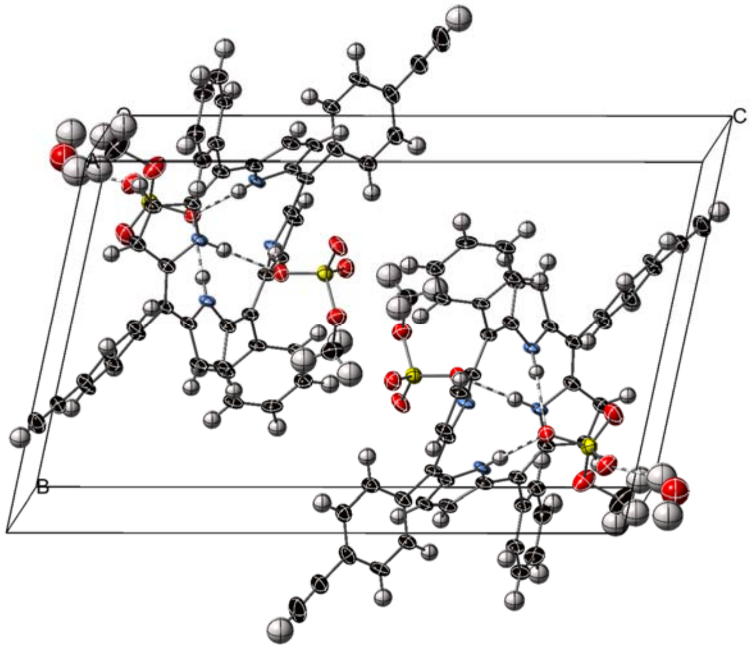

d) The molecules within the unit cell of Compound [H_4**22**][$MeSO_4$]$_2$·¼H_2O (Z = 2). Thermal ellipsoids are shown at 50% probability level.

2.2.17 Figure S2.2.17; $[H_8\textbf{23}][ClO_4]_4{\cdot}2H_2O$ 5,5'-Bis(15-hexyl-10,20-bis(4-methoxyphenyl)porphyrindi-ium) tetrakis(perchlorate) diaqua solvate

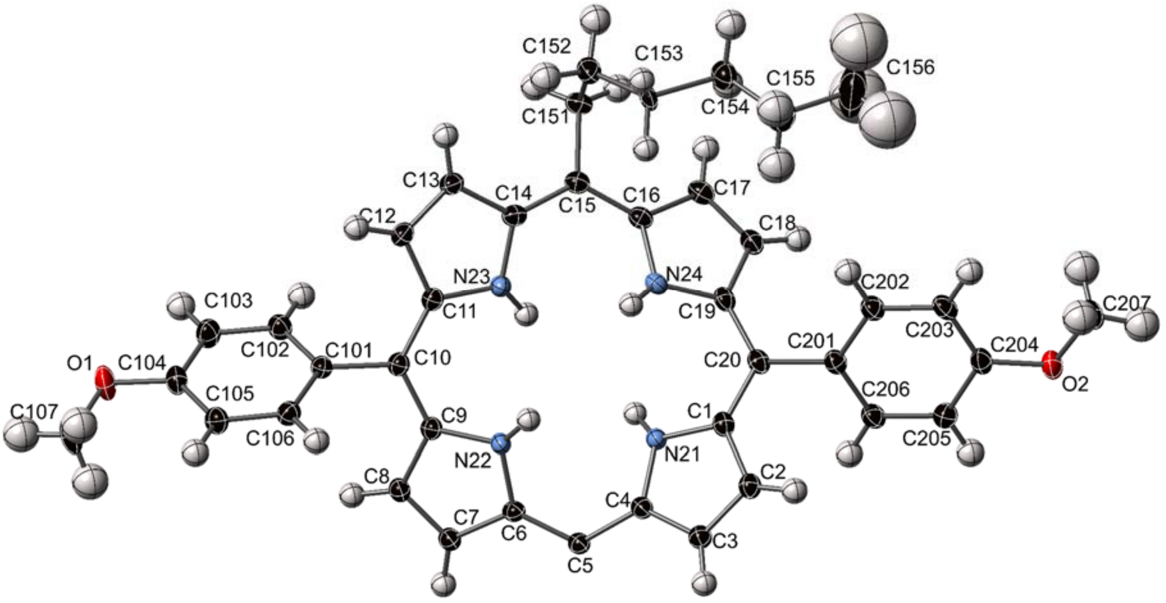

a) A labelled plot of the porphyrin component of the asymmetric unit of compound $[H_8\textbf{23}][ClO_4]_4{\cdot}2H_2O$, 5,5'-Bis(15-hexyl-10,20-bis(4-methoxyphenyl)porphyrindi-ium) tetrakis(perchlorate) diaqua solvate. This molecular fragment is linked to another equivalent porphyrin at the 5- position, and these two halves of the bis-porphyrin are related by a two-fold rotation axis which runs through the centre of the C5 – C5' bond, parallel to the crystallographic *b*-axis, Thermal ellipsoids are shown at 50% probability level.

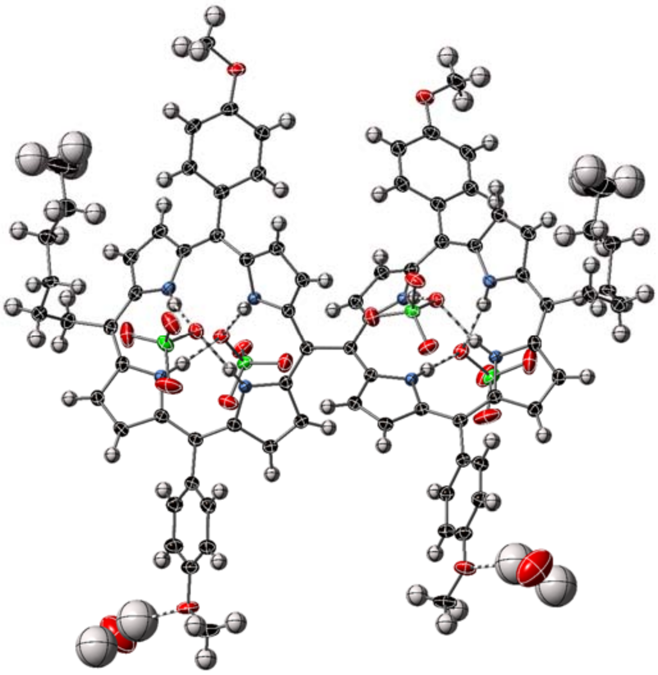

b) A plot of the atoms within the molecular unit, which is twice the asymmetric unit of the crystal structure of compound $[H_8\textbf{23}][ClO_4]_4{\cdot}2H_2O$.

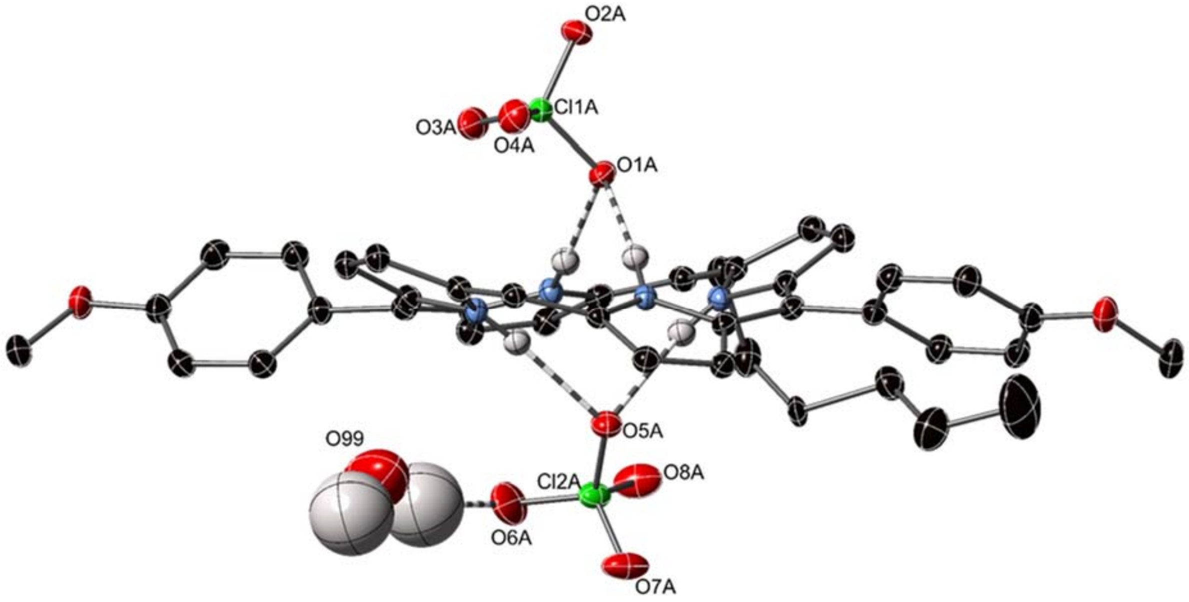

c) A labelled plot of the perchlorate anions and water solvate within the asymmetric unit of compound [H_8**23**][ClO_4]$_4$·2H_2O. Hydrogen bonds are indicated by striped bonds, H-atoms not involved in hydrogen bonding are omitted from this view. Disorder of the *n*-hexyl group at the 15 position of the porphyrin ring is omitted, with only the primary component (C153-C156, 63%) shown in this view.

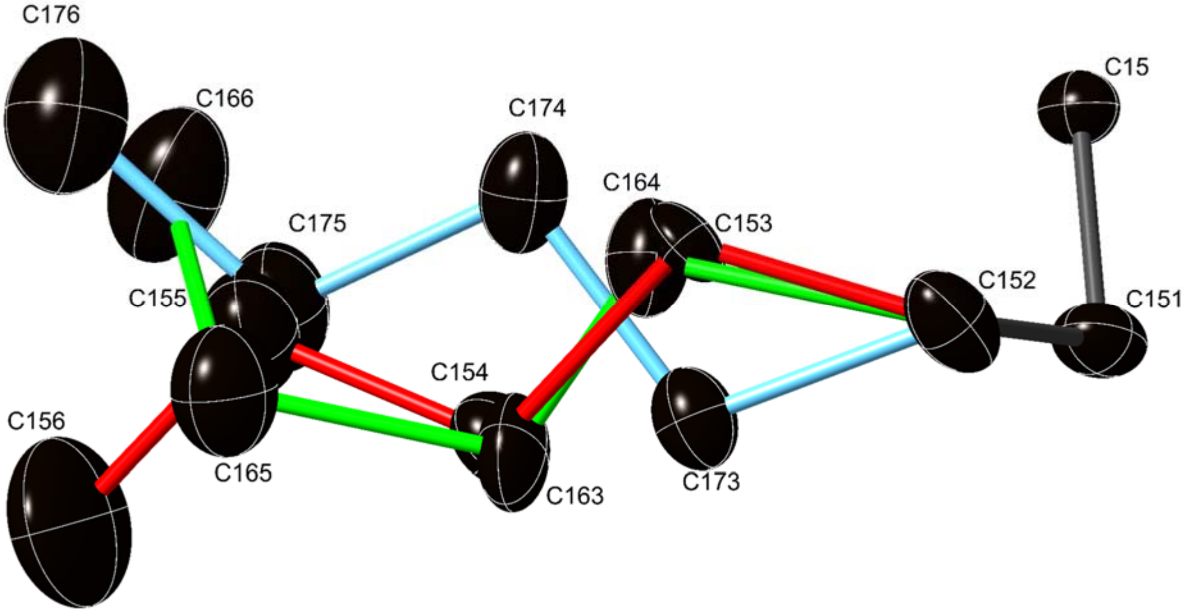

d) A view of the disorder of the alkyl chain at the 15 position of the porphyrin ring. This hexyl chain was modelled as disordered over 3 orientations, sharing the positions C151 and C152 (100%, black bonds), and disordered C153-C156 (63%, red bonds) C163-C166 (26%, green bonds) and C173-C176 (11%, blue bonds). Hydrogen atoms attached to each of these carbon atoms are omitted from view.

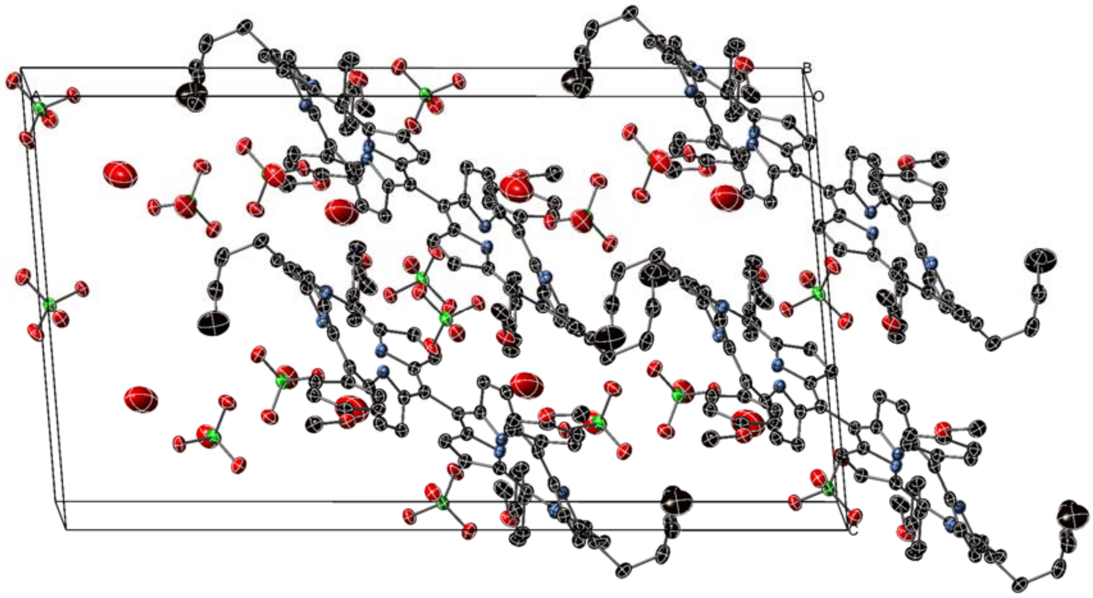

e) The molecules within the unit cell of compound [H_8**23**][ClO_4]$_4$·2H_2O (Z = 4). Thermal ellipsoids are shown at 50% probability level. H-atoms have been omitted from view, as have the minor components of the hexyl group disorder.

2.2.18 Figure S2.2.18; [H_8**24**][CF_3CO_2]$_4$·14H_2O 5,5'-bis(10,15,20-triphenylporphyrindi-ium) tetrakis(trifluoroacetate)

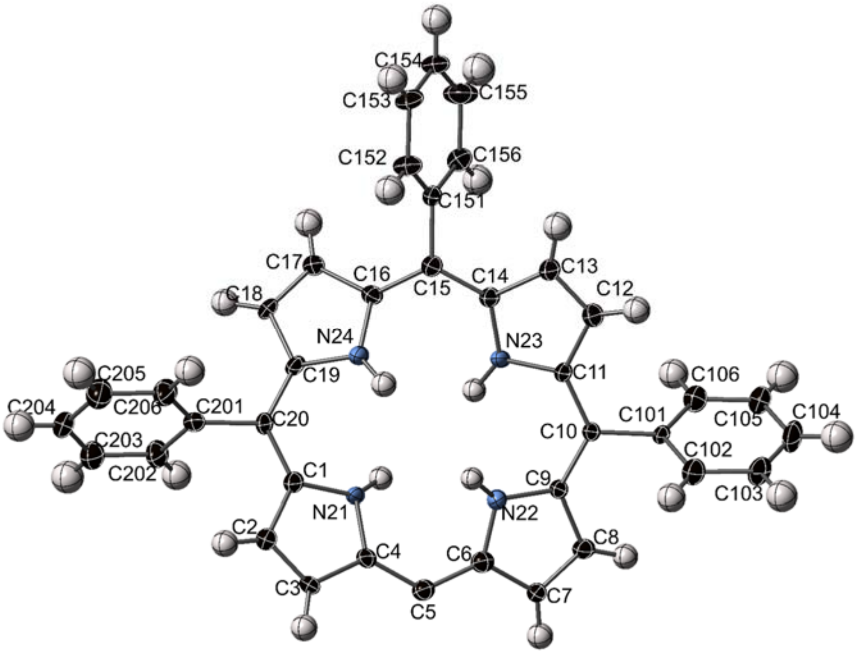

a) A labelled plot of the porphyrin component of the asymmetric unit of compound [H_8**24**][CF_3CO_2]$_4$·14H_2O, 5,5'-bis(10,15,20-triphenylporphyrindi-ium) tetrakis(trifluoroacetate). This molecular fragment is linked to another equivalent porphyrin at the 5- position, and these two halves of the bis-porphyrin are related by a two-fold rotation axis which runs through the centre of the C5 – C5' bond, parallel to the crystallographic *b*- axis, thermal ellipsoids are shown at 50% probability level.

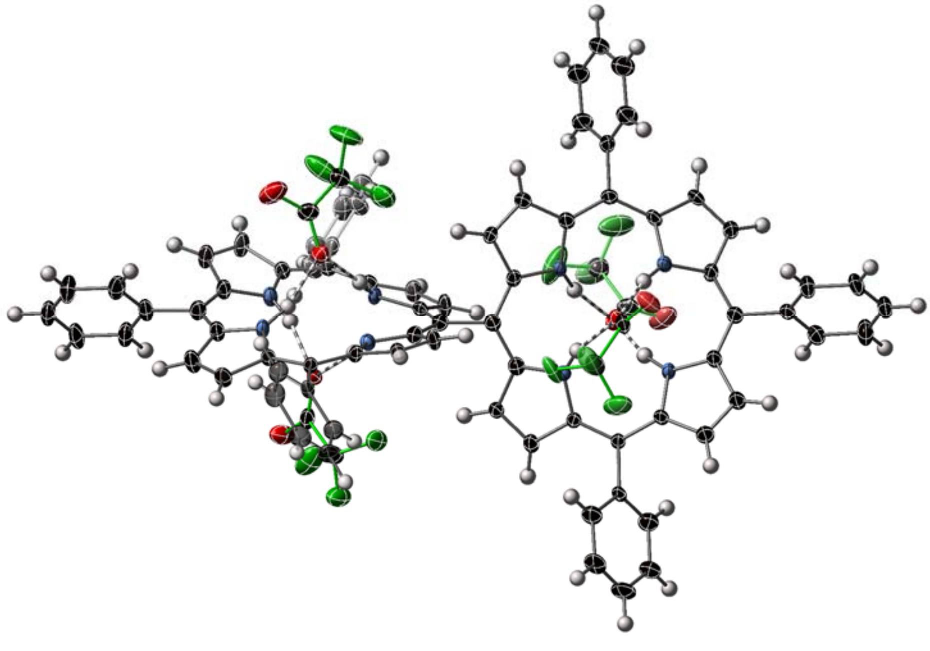

b) A labelled plot molecular unit of Compound [H_8**24**][CF_3CO_2]$_4$·14H_2O, which comprises two of the asymmetric units. Non-H atoms are represented as thermal ellipsoids, H-atoms are represented as spheres of radius 0.3 Å. Thermal ellipsoids are shown at 50% probability level.

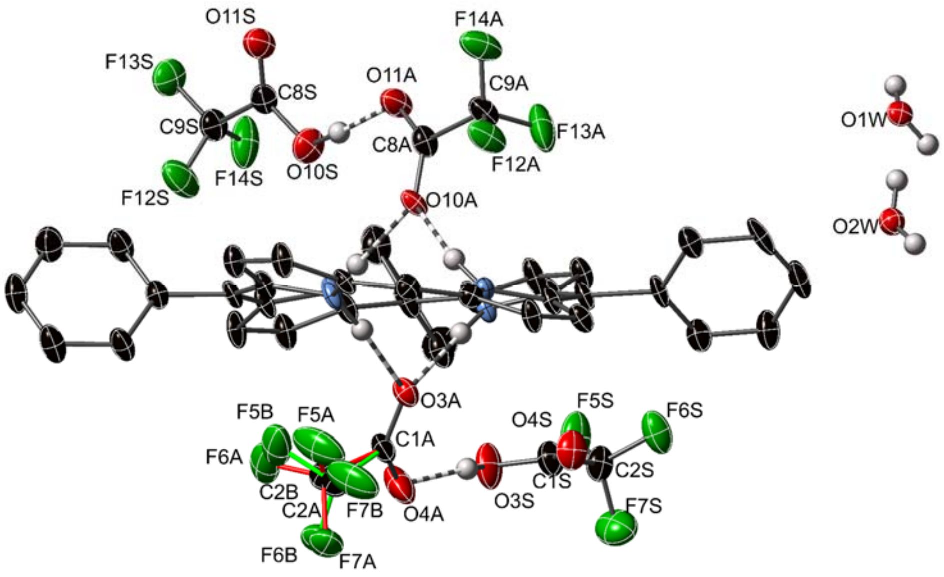

c) A labelled plot of the anions and solvent molecules in the asymmetric unit of compound $[H_8\mathbf{24}][CF_3CO_2]_4\cdot 14H_2O$. Non-H atoms are represented as thermal ellipsoids, H-atoms are represented as spheres of radius 0.3 Å, H-atoms not involved in hydrogen bonding have been omitted. Thermal ellipsoids are shown at 50% probability level.

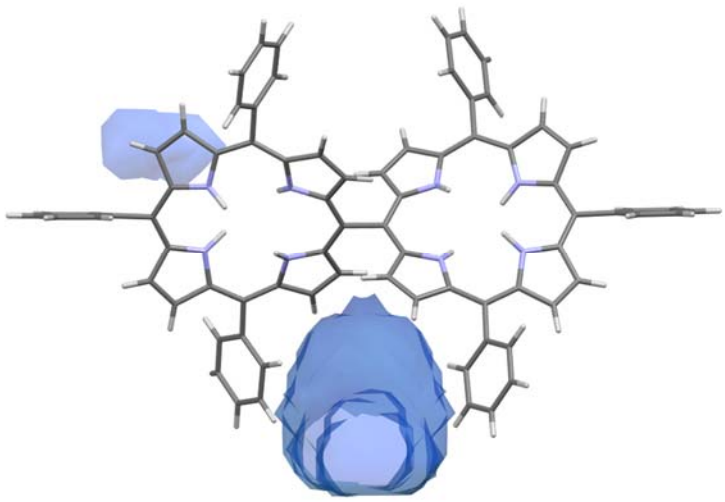

d) A plot of solvent voids within the crystal structure of compound $[H_8\mathbf{24}][CF_3CO_2]_4\cdot 14H_2O$ which had to be accounted for with Squeeze, in Platon. Atoms of the main fragment, anions, solvent are omitted from view. 4 equivalent disconnected voids, one per porphyrin dimer, are present within the unit cell, each containing 14 disordered water molecules, assigned from the electron density (550 e- per unit cell).

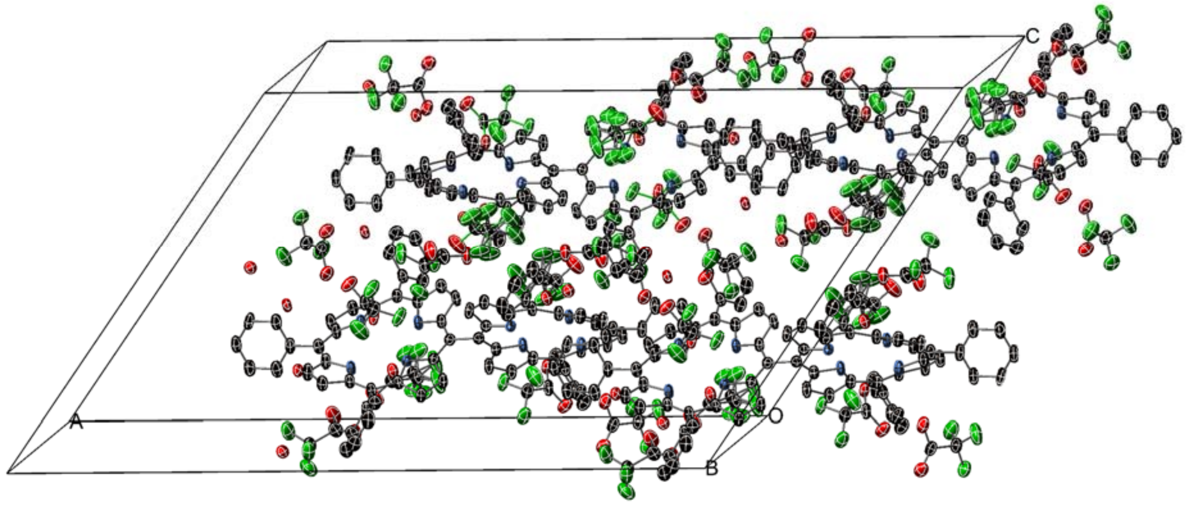

e) A plot of atoms within the unit cell of compound [H_8**24**][CF_3CO_2]$_4$·14H_2O (Z = 4). H-atoms are omitted, Thermal parameters are shown at a 50% probability level.

2.3 Normal-coordinate Structural Decomposition (NSD)

Normal-coordinate structural decomposition was performed using a new implementation (Kingsbury and Senge) of the porphyrin NSD method, with this tool available online at https://chemistry.tcd.ie/staff/people/mos/NSD.html using the methodology of Jentzen *et al* (W. Jentzen, X. Z. Song, and J. A. Shelnutt, **1997**, *J. Phys. Chem. B, 101,* 9, 1684-1699). A publication relating to the use and interpretation of these results is currently in preparation.

The critical values in this analysis are the B_{2u}(1) and B_{2u}(2), for saddle-shaped porphyrins, B_{2g}(1) and B_{2g}(2) for 5,15-disubstituted porphyrins, and B_{1g}(2), which is indicative of freebases. A_{1g}(2) and A_{1g}(3) are generally correlated with saddle-shaped out-of-plane distortions, representing the in-plane compensation for these out-of-plane distortions.

2.3.1 NSD result generated from H_2**11** (5,15-diphenylporphyrin)

Summary of the NSD (in Å):

**basis**	**Δ_{ip}**	**δ_{ip}**	**B_{2g}**	**B_{1g}**	**$E_u(x)$**	**$E_u(y)$**	**A_{1g}**	**A_{2g}**
min.	0.38	0.02	0.31	-0.06	0.00	0.00	0.22	0.01
ext.	0.40	0.01	0.31	-0.06	0.00	0.00	0.22	0.01
			0.06	-0.08	0.00	0.00	-0.02	0.00
total	0.41	0.00	0.31	-0.06	0.00	0.00	0.22	0.01
			0.06	-0.09	0.00	0.00	-0.02	0.00
			0.01	-0.07	0.00	0.00	0.05	0.00
			0.00	0.00	0.00	0.00	0.00	0.00
			0.00	0.01	0.00	0.00	0.02	0.00
			0.01	-0.01	0.00	0.00	0.01	
					0.00	0.00		
					0.00	0.00		
					0.00	0.00		
					0.00	0.00		
					0.00	0.00		
comp.	0.41	0.00	0.31	0.13	0.00	0.00	0.23	0.02

**basis**	**Δ_{oop}**	**δ_{oop}**	**B_{2u}**	**B_{1u}**	**A_{2u}**	**$E_g(x)$**	**$E_g(y)$**	**A_{1u}**
min.	0.21	0.06	0.00	0.00	0.00	0.12	-0.17	0.00
ext.	0.32	0.00	0.00	0.00	0.00	0.13	-0.17	0.00
			0.00	0.00	0.00	0.23	-0.06	0.00
total	0.32	0.00	0.00	0.00	0.00	0.13	-0.17	0.00
			0.00	0.00	0.00	0.23	-0.06	0.00
			0.00	0.00	0.00	0.02	0.03	
						0.00	0.01	
						0.00	-0.01	
comp.	0.32	0.00	0.00	0.00	0.00	0.26	0.19	0.00

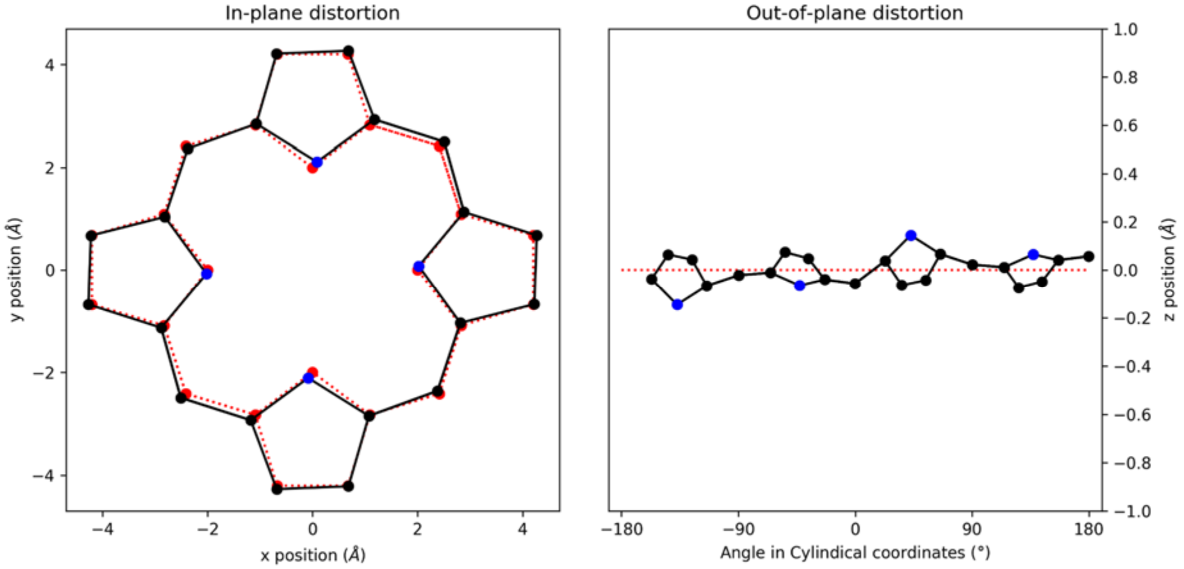

Figure S2.01; (a) out-of-plane and (b) in-plane skeletal plots of the porphyrin core.
Porphyrin is represented in black(C) and blue(N), with the reference structure (CuTPP) in red lines.

2.3.2 NSD result generated from H_2**11**·CH_2Cl_2 (5,15-diphenylporphyrin DCM)

Summary of the NSD (in Å):

**basis**	**Δ_{ip}**	**δ_{ip}**	**B_{2g}**	**B_{1g}**	**$E_u(x)$**	**$E_u(y)$**	**A_{1g}**	**A_{2g}**
min.	0.40	0.01	-0.35	-0.04	-0.01	-0.01	0.19	0.00
ext.	0.41	0.00	-0.36	-0.04	-0.01	-0.01	0.19	0.00
			-0.07	-0.02	0.00	0.01	0.00	0.00
total	0.42	0.00	-0.35	-0.04	-0.01	-0.01	0.19	0.00
			-0.07	-0.02	0.00	0.01	0.00	0.00
			-0.01	-0.02	0.00	0.00	0.05	-0.01
			0.01	0.00	0.00	0.00	-0.02	0.00
			0.00	-0.01	0.00	0.00	0.00	0.00
			-0.01	-0.01	0.00	0.00	0.02	
					0.00	0.00		
					0.00	0.00		
					0.00	0.00		
					0.00	0.00		
					0.00	-0.01		
comp.	0.42	0.00	0.36	0.05	0.01	0.02	0.20	0.01

**basis**	**Δ_{oop}**	**δ_{oop}**	**B_{2u}**	**B_{1u}**	**A_{2u}**	**$E_g(x)$**	**$E_g(y)$**	**A_{1u}**
min.	0.46	0.00	0.04	-0.45	0.08	0.02	0.01	0.00
ext.	0.46	0.00	0.03	-0.45	0.08	0.02	0.01	0.00
			-0.05	-0.01	-0.02	-0.02	0.00	-0.01
total	0.47	0.00	0.03	-0.45	0.08	0.02	0.01	0.00
			-0.05	-0.01	-0.02	-0.02	0.00	-0.01
			0.00	-0.01	0.01	0.00	0.00	
						0.00	0.00	
						0.00	0.00	
comp.	0.47	0.00	0.06	0.45	0.08	0.03	0.01	0.01

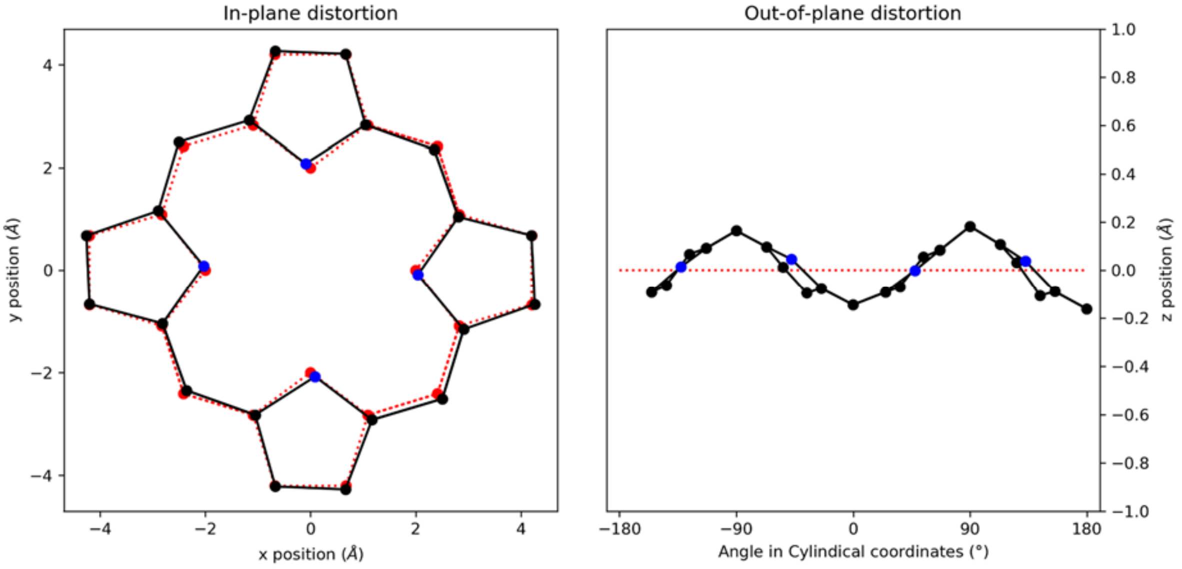

Figure S2.02; (a) out-of-plane and (b) in-plane skeletal plots of the porphyrin core.
Porphyrin is represented in black(C) and blue(N), with the reference structure (CuTPP) in red lines.

2.3.3 NSD result generated from [H_4**11**][CF_3CO_2]$_2$ (5,15-diphenylporphyrindi-ium bis(trifluoroacetate))

Summary of the NSD (in Å):

basis	Δ_{ip}	δ_{ip}	B_{2g}	B_{1g}	$E_u(x)$	$E_u(y)$	A_{1g}	A_{2g}
min.	0.30	0.14	-0.28	-0.07	0.01	-0.01	0.09	-0.01
ext.	0.35	0.10	-0.28	-0.07	0.01	-0.01	0.09	-0.01
			-0.04	0.00	0.01	-0.02	-0.17	-0.01
total	0.48	0.00	-0.28	-0.07	0.01	-0.01	0.11	-0.01
			-0.04	0.00	0.01	-0.02	-0.17	-0.01
			-0.02	0.06	0.01	-0.01	0.31	0.00
			0.00	0.00	0.00	0.01	-0.01	0.00
			0.00	0.00	0.01	0.00	0.00	0.00
			-0.01	-0.01	0.00	0.00	-0.01	
					0.00	0.00		
					0.00	0.00		
					0.00	0.00		
					0.00	0.00		
					0.00	0.00		
comp.	0.48	0.00	0.28	0.09	0.02	0.02	0.37	0.02

basis	Δ_{oop}	δ_{oop}	B_{2u}	B_{1u}	A_{2u}	$E_g(x)$	$E_g(y)$	A_{1u}
min.	1.84	0.31	1.83	-0.09	0.18	-0.05	0.01	0.04
ext.	1.91	0.00	1.82	-0.09	0.18	-0.05	0.01	0.04
			-0.55	-0.01	-0.01	0.05	-0.05	-0.01
total	1.92	0.00	1.82	-0.09	0.18	-0.05	0.01	0.04
			-0.55	-0.01	-0.01	0.05	-0.05	-0.01
			-0.05	0.00	0.01	0.02	-0.01	
						0.00	-0.01	
						0.00	0.00	
comp.	1.92	0.00	1.90	0.09	0.18	0.07	0.05	0.04

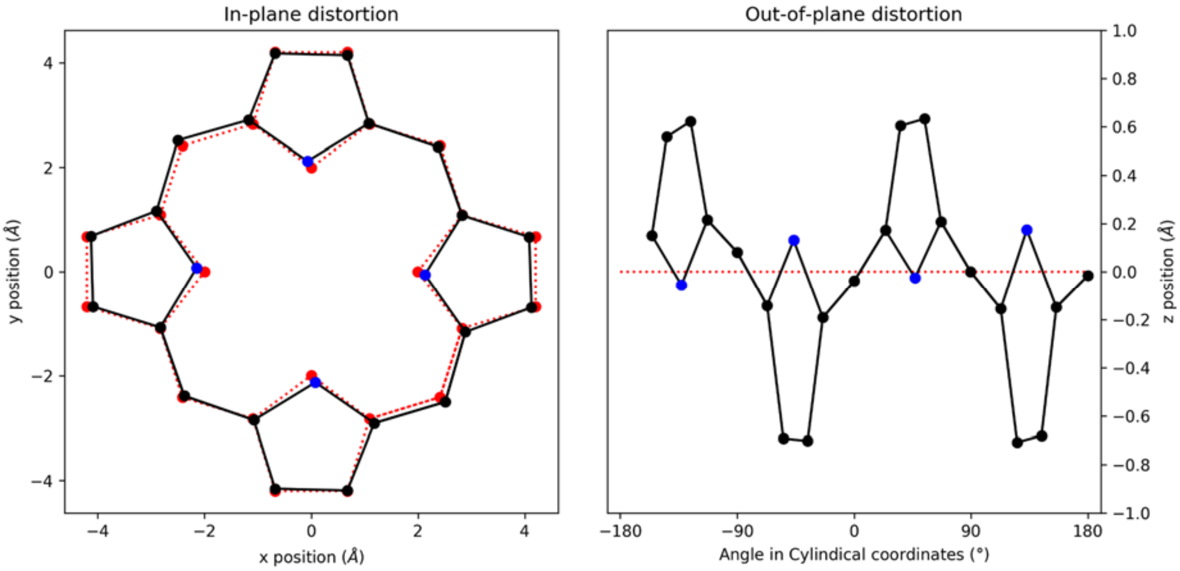

Figure S2.03; (a) out-of-plane and (b) in-plane skeletal plots of the porphyrin core.
Porphyrin is represented in black(C) and blue(N), with the reference structure (CuTPP) in red lines.

2.3.4 NSD result generated from [H_4**11**][CF_3CO_2]$_2$·2CF_3CO_2H (5,15-diphenylporphyrindi-ium bis(trifluoroacetate) bis(trifluoroacetic acid solvate))

Summary of the NSD (in Å):

**basis**	**Δ_{ip}**	**δ_{ip}**	**B_{2g}**	**B_{1g}**	**$E_u(x)$**	**$E_u(y)$**	**A_{1g}**	**A_{2g}**
min.	0.25	0.06	0.12	-0.05	0.01	-0.01	0.21	0.02
ext.	0.28	0.05	0.12	-0.05	0.01	-0.01	0.20	0.02
			0.03	0.00	0.01	-0.01	-0.12	0.04
total	0.36	0.00	0.12	-0.05	0.01	-0.01	0.22	0.02
			0.03	0.00	0.01	-0.01	-0.11	0.04
			0.02	0.00	0.01	0.00	0.22	0.01
			-0.01	0.00	0.00	0.00	-0.01	0.01
			0.00	0.00	0.00	-0.01	-0.02	0.00
			0.02	0.00	0.00	0.00	0.00	
					0.01	0.00		
					0.00	0.00		
					0.00	0.00		
					0.00	0.00		
					0.00	0.00		
comp.	0.36	0.00	0.13	0.05	0.02	0.02	0.33	0.05

**basis**	**Δ_{oop}**	**δ_{oop}**	**B_{2u}**	**B_{1u}**	**A_{2u}**	**$E_g(x)$**	**$E_g(y)$**	**A_{1u}**
min.	1.32	0.24	-1.28	-0.29	0.00	-0.04	0.01	0.02
ext.	1.40	0.00	-1.27	-0.29	0.00	-0.04	0.01	0.02
			0.49	0.00	0.02	-0.01	0.01	-0.01
total	1.40	0.00	-1.27	-0.29	0.00	-0.04	0.01	0.02
			0.50	0.00	0.02	-0.01	0.01	-0.01
			0.04	0.00	0.00	0.00	0.00	
						0.00	0.00	
						-0.01	-0.01	
comp.	1.40	0.00	1.37	0.29	0.02	0.04	0.02	0.03

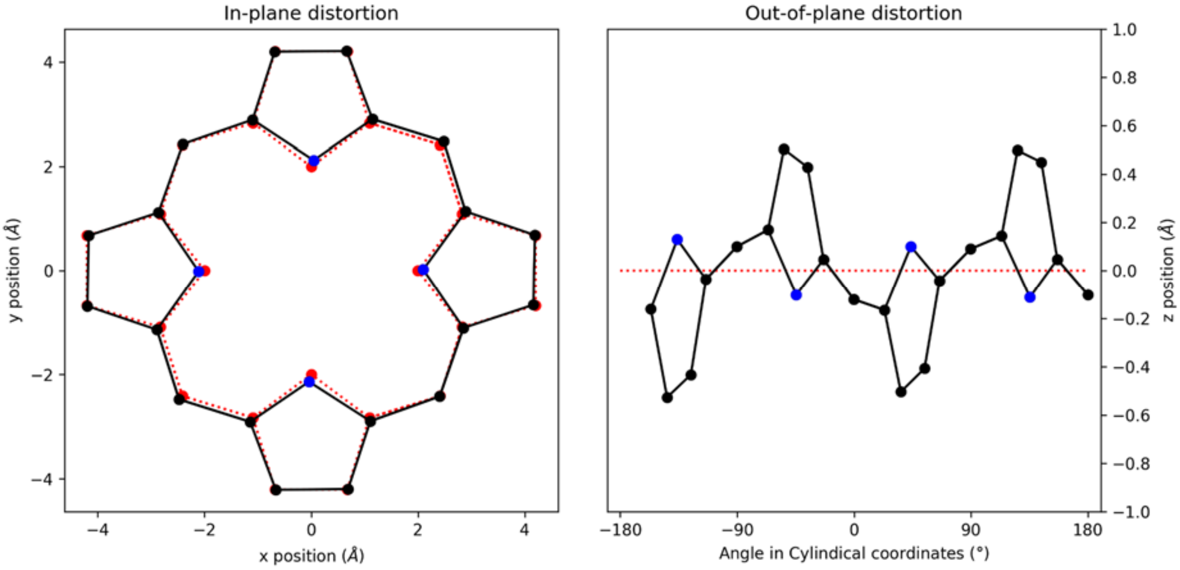

Figure S2.04; (a) out-of-plane and (b) in-plane skeletal plots of the porphyrin core.
Porphyrin is represented in black(C) and blue(N), with the reference structure (CuTPP) in red lines.

2.3.5 NSD result generated from H_2**13**·⅓(CH_2Cl_2) (5,15-bis(3-pentyl)porphyrin 1/3(DCM))

Summary of the NSD (in Å):

**basis**	Δ_{ip}	δ_{ip}	B_{2g}	B_{1g}	$E_u(x)$	$E_u(y)$	A_{1g}	A_{2g}
min.	0.73	0.02	0.69	-0.07	0.00	0.00	0.23	0.00
ext.	0.74	0.01	0.69	-0.07	0.00	0.00	0.23	0.00
			0.11	-0.06	0.00	0.00	-0.02	0.00
total	0.74	0.00	0.69	-0.07	0.00	0.00	0.24	0.00
			0.11	-0.06	0.00	0.00	-0.02	0.00
			0.01	-0.04	0.00	0.00	0.06	0.00
			-0.01	0.00	0.00	0.00	-0.01	0.00
			0.01	0.00	0.00	0.00	0.01	0.00
			0.01	-0.01	0.00	0.00	0.02	
					0.00	0.00		
					0.00	0.00		
					0.00	0.00		
					0.00	0.00		
					0.00	0.00		
comp.	0.74	0.00	0.70	0.10	0.00	0.00	0.25	0.01

**basis**	Δ_{oop}	δ_{oop}	B_{2u}	B_{1u}	A_{2u}	$E_g(x)$	$E_g(y)$	A_{1u}
min.	0.02	0.00	0.00	0.00	0.00	-0.02	0.00	0.00
ext.	0.04	0.00	0.00	0.00	0.00	-0.02	0.00	0.00
			0.00	0.00	0.00	0.03	0.02	0.00
total	0.04	0.00	0.00	0.00	0.00	-0.02	0.00	0.00
			0.00	0.00	0.00	0.03	0.02	0.00
			0.00	0.00	0.00	0.01	0.00	
						0.00	0.00	
						0.00	0.00	
comp.	0.04	0.00	0.00	0.00	0.00	0.04	0.02	0.00

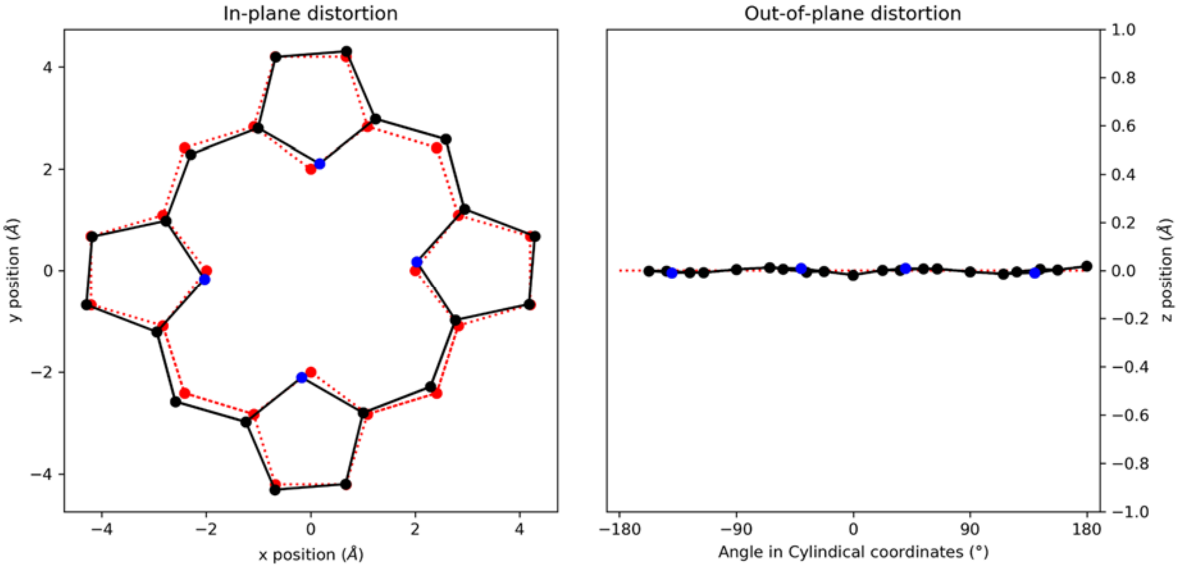

Figure S2.05; (a) out-of-plane and (b) in-plane skeletal plots of the porphyrin core.
Porphyrin is represented in black(C) and blue(N), with the reference structure (CuTPP) in red lines.

2.3.6 NSD result generated from $[H_4\mathbf{13}][CF_3CO_2]_2$ (5,15-bis(3-pentyl)porphyrindi-ium bis(trifluoroacetate))

Summary of the NSD (in Å):

basis	Δ_{ip}	δ_{ip}	B_{2g}	B_{1g}	$E_u(x)$	$E_u(y)$	A_{1g}	A_{2g}
min.	0.72	0.08	-0.67	0.05	0.00	0.00	0.27	0.01
ext.	0.75	0.05	-0.67	0.05	0.00	0.00	0.27	0.01
			-0.16	0.00	0.00	0.00	-0.10	0.01
total	0.78	0.00	-0.67	0.05	0.00	0.00	0.28	0.01
			-0.16	0.00	0.00	0.00	-0.10	0.01
			-0.03	0.00	0.00	0.00	0.22	0.01
			-0.02	0.00	0.00	0.00	-0.02	0.00
			0.00	0.00	0.00	0.00	0.00	-0.01
			-0.01	0.00	0.00	0.00	0.01	
					0.00	0.00		
					0.00	0.00		
					0.00	0.00		
					0.00	0.00		
					0.00	0.00		
comp.	0.78	0.00	0.69	0.05	0.00	0.00	0.37	0.02

basis	Δ_{oop}	δ_{oop}	B_{2u}	B_{1u}	A_{2u}	$E_g(x)$	$E_g(y)$	A_{1u}
min.	0.98	0.12	0.00	0.00	0.00	0.76	0.62	0.00
ext.	1.04	0.00	0.00	0.00	0.00	0.76	0.63	0.00
			0.00	0.00	0.00	0.20	0.27	0.00
total	1.05	0.00	0.00	0.00	0.00	0.76	0.63	0.00
			0.00	0.00	0.00	0.20	0.27	0.00
			0.00	0.00	0.00	-0.01	0.03	
						0.01	0.02	
						0.04	0.04	
comp.	1.05	0.00	0.00	0.00	0.00	0.79	0.69	0.00

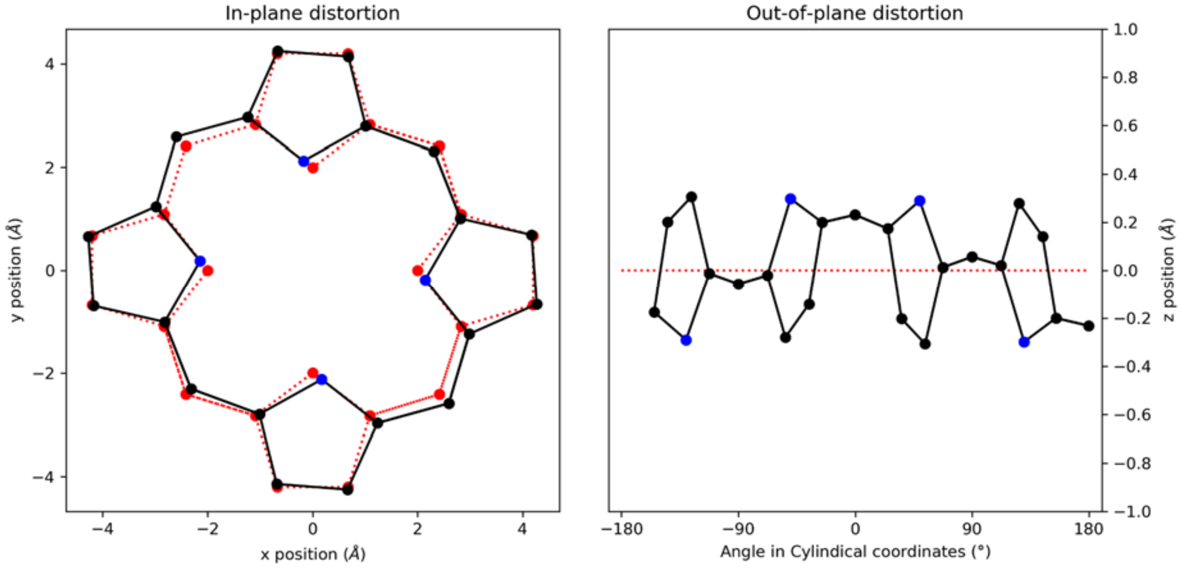

Figure S2.06; (a) out-of-plane and (b) in-plane skeletal plots of the porphyrin core.
Porphyrin is represented in black(C) and blue(N), with the reference structure (CuTPP) in red lines.

2.3.7 NSD result generated from $[H_4\mathbf{14}][CF_3CO_2]_2$ (5,15-triphenylporphyrindi-ium bis(trifluoroacetate))

Summary of the NSD (in Å):

basis	Δ_{ip}	δ_{ip}	B_{2g}	B_{1g}	$E_u(x)$	$E_u(y)$	A_{1g}	A_{2g}
min.	0.22	0.14	-0.19	-0.03	0.00	-0.01	0.11	0.01
ext.	0.30	0.10	-0.19	-0.03	0.00	-0.01	0.11	0.01
			-0.03	-0.01	-0.07	0.01	-0.18	0.02
total	0.44	0.00	-0.19	-0.03	0.00	-0.01	0.12	0.01
			-0.03	-0.01	-0.07	0.01	-0.17	0.02
			-0.01	0.00	0.00	0.00	0.32	0.00
			0.00	0.00	0.03	-0.01	-0.02	0.00
			-0.01	0.00	0.04	-0.01	0.00	0.00
			0.00	0.00	0.01	-0.01	-0.01	
					0.00	0.00		
					0.00	0.00		
					-0.01	0.00		
					-0.01	0.01		
					-0.01	0.00		
comp.	0.44	0.00	0.19	0.04	0.09	0.03	0.38	0.02

basis	Δ_{oop}	δ_{oop}	B_{2u}	B_{1u}	A_{2u}	$E_g(x)$	$E_g(y)$	A_{1u}
min.	1.76	0.39	1.75	0.10	0.00	0.04	-0.16	0.02
ext.	1.86	0.00	1.74	0.10	0.00	0.05	-0.16	0.02
			-0.62	0.01	-0.03	0.01	0.00	-0.01
total	1.86	0.00	1.74	0.10	0.00	0.05	-0.16	0.02
			-0.62	0.01	-0.03	0.01	0.00	-0.01
			-0.05	0.00	0.01	0.01	0.02	
						0.00	0.01	
						0.00	-0.02	
comp.	1.86	0.00	1.85	0.11	0.03	0.05	0.16	0.02

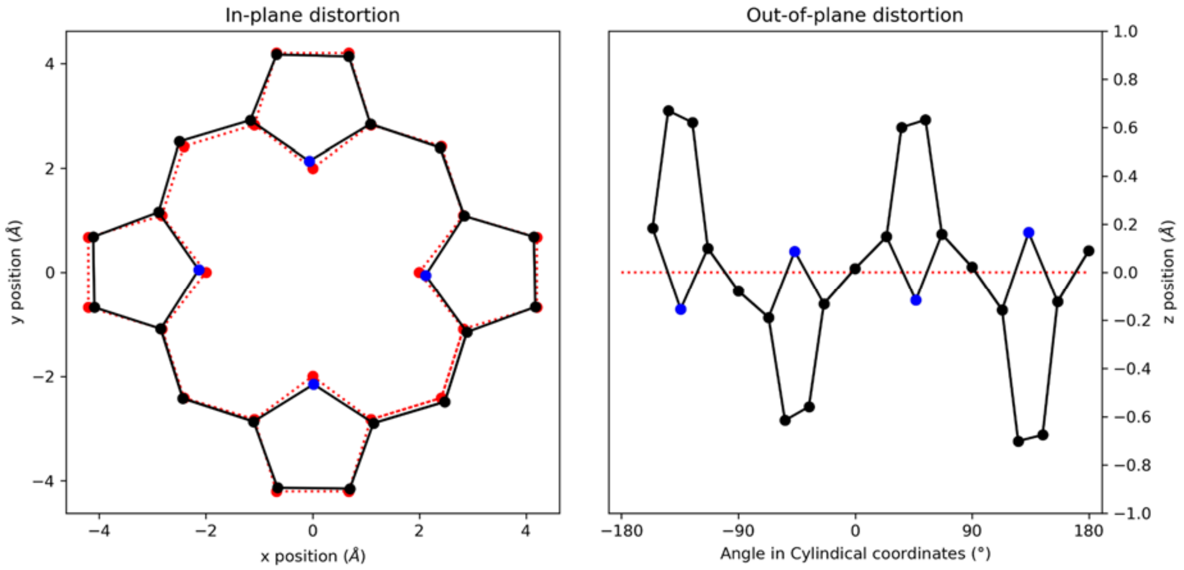

Figure S2.07; (a) out-of-plane and (b) in-plane skeletal plots of the porphyrin core. Porphyrin is represented in black(C) and blue(N), with the reference structure (CuTPP) in red lines.

2.3.8 NSD result generated from H_2**15** (5,15-bis(4-butoxyphenyl)porphyrin)

Summary of the NSD (in Å):

**basis**	**Δ_{ip}**	**δ_{ip}**	**B_{2g}**	**B_{1g}**	**$E_u(x)$**	**$E_u(y)$**	**A_{1g}**	**A_{2g}**
min.	0.39	0.01	-0.33	0.04	0.00	0.00	0.21	0.01
ext.	0.39	0.00	-0.33	0.04	0.00	0.00	0.21	0.01
			-0.04	0.04	0.00	0.00	-0.02	0.00
total	0.40	0.00	-0.33	0.04	0.00	0.00	0.21	0.01
			-0.04	0.04	0.00	0.00	-0.02	0.00
			-0.01	0.03	0.00	0.00	0.04	0.00
			0.00	0.00	0.00	0.00	0.01	0.00
			0.00	0.00	0.00	0.00	0.02	0.00
			-0.01	0.01	0.00	0.00	0.01	
					0.00	0.00		
					0.00	0.00		
					0.00	0.00		
					0.00	0.00		
					0.00	0.00		
comp.	0.40	0.00	0.33	0.07	0.00	0.00	0.22	0.01

**basis**	**Δ_{oop}**	**δ_{oop}**	**B_{2u}**	**B_{1u}**	**A_{2u}**	**$E_g(x)$**	**$E_g(y)$**	**A_{1u}**
min.	0.06	0.00	0.00	0.00	0.00	-0.06	-0.02	0.00
ext.	0.06	0.00	0.00	0.00	0.00	-0.06	-0.02	0.00
			0.00	0.00	0.00	0.00	-0.01	0.00
total	0.06	0.00	0.00	0.00	0.00	-0.06	-0.02	0.00
			0.00	0.00	0.00	0.00	-0.01	0.00
			0.00	0.00	0.00	0.00	0.01	
						0.01	0.01	
						0.00	0.00	
comp.	0.06	0.00	0.00	0.00	0.00	0.06	0.02	0.00

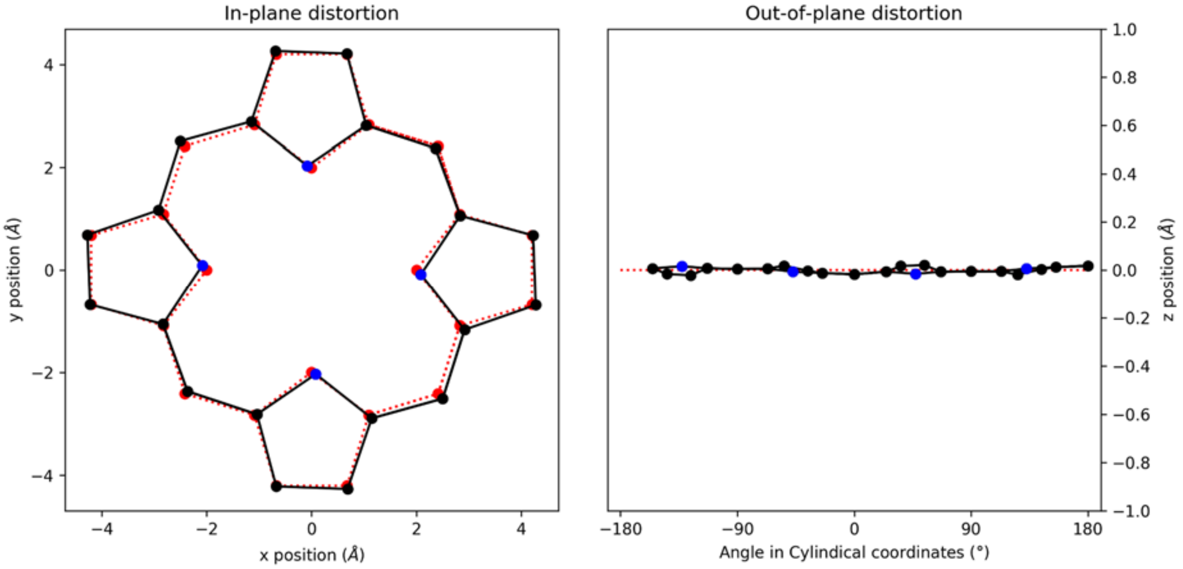

Figure S2.08; (a) out-of-plane and (b) in-plane skeletal plots of the porphyrin core.
Porphyrin is represented in black(C) and blue(N), with the reference structure (CuTPP) in red lines.

2.3.9 NSD result generated from $[H_4\textbf{17}][CF_3CO_2]_2\cdot 2CF_3CO_2H$ (5,15-bis(4-methoxyphenyl)porphyrindi-ium bis(trifluoroacetate) bis(trifluoroacetic acid solvate))

Summary of the NSD (in Å):

basis	Δ_{ip}	δ_{ip}	B_{2g}	B_{1g}	$E_u(x)$	$E_u(y)$	A_{1g}	A_{2g}
min.	0.33	0.06	-0.19	-0.03	0.01	-0.02	0.27	0.01
ext.	0.36	0.04	-0.19	-0.03	0.01	-0.02	0.27	0.01
			-0.04	0.00	0.01	-0.03	-0.11	0.02
total	0.42	0.00	-0.19	-0.03	0.00	-0.02	0.28	0.01
			-0.04	0.00	0.01	-0.03	-0.11	0.02
			-0.02	0.02	0.01	-0.01	0.21	0.00
			0.01	0.00	-0.01	0.01	0.00	0.00
			0.00	0.00	-0.02	0.03	0.00	0.00
			-0.01	-0.01	0.00	0.00	-0.01	
					0.00	0.00		
					0.00	0.00		
					0.00	0.00		
					0.00	0.00		
					0.00	0.00		
comp.	0.42	0.00	0.20	0.04	0.03	0.05	0.37	0.02

basis	Δ_{oop}	δ_{oop}	B_{2u}	B_{1u}	A_{2u}	$E_g(x)$	$E_g(y)$	A_{1u}
min.	1.19	0.24	-1.16	-0.15	-0.10	0.16	-0.11	-0.02
ext.	1.28	0.00	-1.16	-0.15	-0.10	0.16	-0.11	-0.02
			0.49	0.00	0.01	0.02	0.03	0.01
total	1.28	0.00	-1.15	-0.15	-0.10	0.16	-0.11	-0.02
			0.49	0.00	0.01	0.02	0.03	0.01
			0.03	0.00	0.00	0.00	0.01	
						0.00	0.00	
						0.02	-0.01	
comp.	1.28	0.00	1.26	0.15	0.10	0.16	0.12	0.02

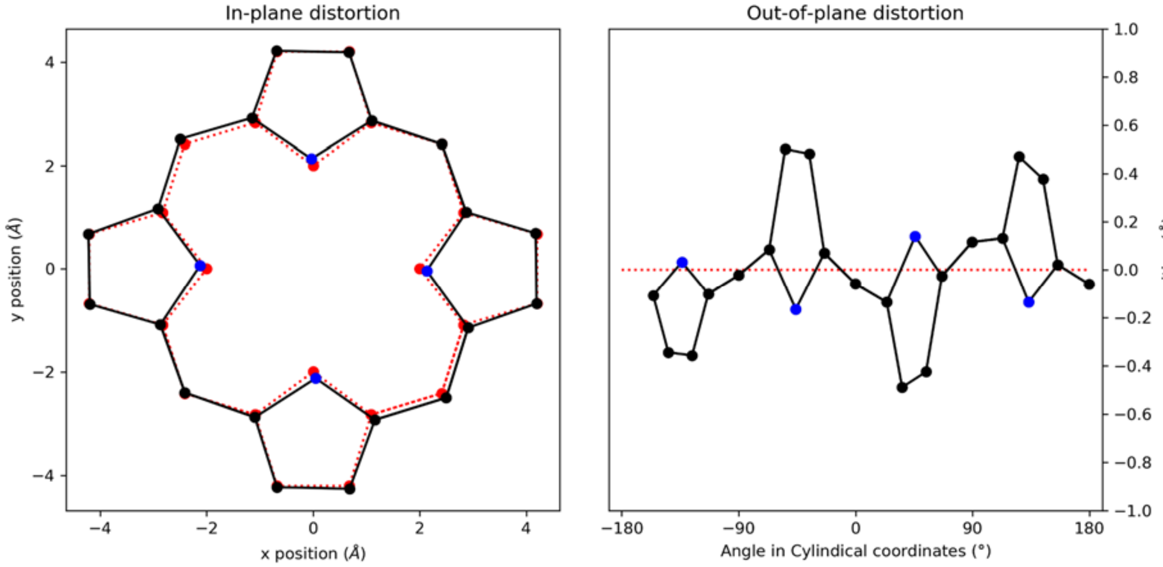

Figure S2.09; (a) out-of-plane and (b) in-plane skeletal plots of the porphyrin core.
Porphyrin is represented in black(C) and blue(N), with the reference structure (CuTPP) in red lines.

2.3.10 NSD result generated from $[H_4\mathbf{18}][ClO_4]_2$ (5,15-bis(4-bromophenyl)porphyrindi-ium bis(perchlorate)) (HE)

Summary of the NSD (in Å):

basis	Δ_{ip}	δ_{ip}	B_{2g}	B_{1g}	$E_u(x)$	$E_u(y)$	A_{1g}	A_{2g}
min.	0.33	0.16	0.31	0.09	0.00	-0.03	0.06	-0.02
ext.	0.38	0.12	0.31	0.09	0.00	-0.03	0.06	-0.02
			0.03	0.00	0.00	-0.01	-0.18	-0.07
total	0.52	0.00	0.31	0.09	0.00	-0.03	0.07	-0.02
			0.03	0.00	0.00	-0.01	-0.18	-0.07
			0.02	-0.08	0.01	0.00	0.33	0.00
			-0.02	-0.01	-0.01	0.01	-0.02	0.00
			0.01	0.01	0.00	0.01	-0.02	0.00
			0.01	0.02	0.00	0.00	0.00	
					0.01	0.00		
					0.00	0.00		
					0.00	0.00		
					0.00	0.00		
					0.00	0.00		
comp.	0.52	0.00	0.32	0.12	0.01	0.03	0.38	0.07

basis	Δ_{oop}	δ_{oop}	B_{2u}	B_{1u}	A_{2u}	$E_g(x)$	$E_g(y)$	A_{1u}
min.	1.92	0.26	1.87	-0.33	-0.23	-0.05	0.04	-0.05
ext.	1.97	0.00	1.86	-0.33	-0.23	-0.05	0.04	-0.05
			-0.50	0.00	0.01	0.00	-0.01	0.02
total	1.97	0.00	1.86	-0.33	-0.23	-0.05	0.04	-0.05
			-0.51	0.00	0.01	0.00	-0.01	0.02
			-0.04	0.00	-0.01	0.00	0.00	
						0.00	0.00	
						0.01	0.00	
comp.	1.97	0.00	1.93	0.33	0.23	0.05	0.04	0.06

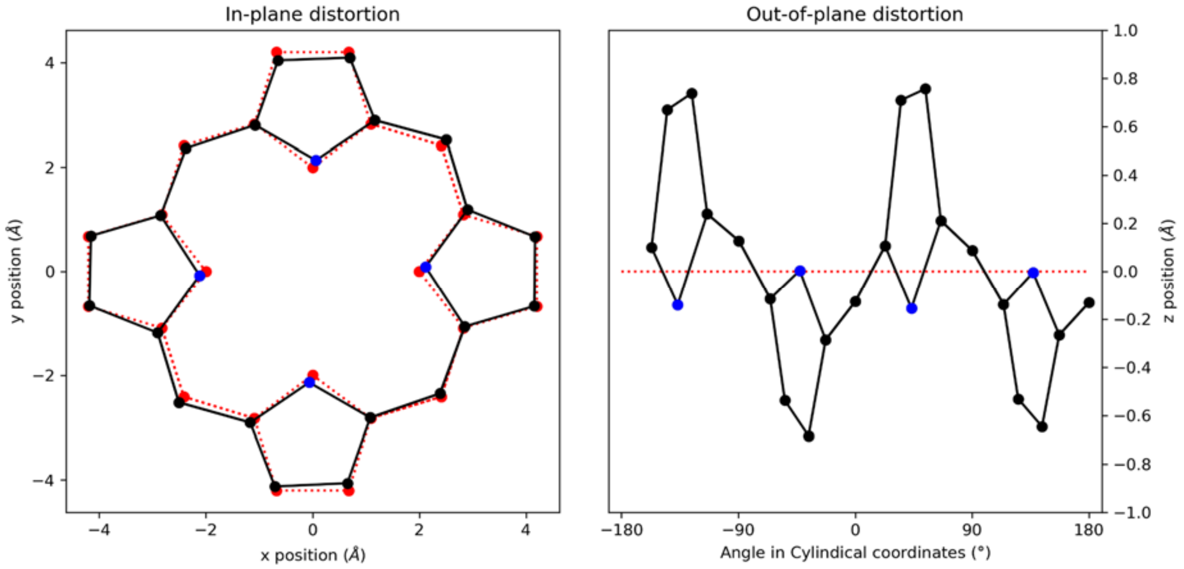

Figure S2.10; (a) out-of-plane and (b) in-plane skeletal plots of the porphyrin core.
Porphyrin is represented in black(C) and blue(N), with the reference structure (CuTPP) in red lines.

2.3.11 NSD result generated from [H_4**18**][ClO_4]$_2$ (5,15-bis(4-bromophenyl)porphyrindi-ium bis(perchlorate)) (MS)

Summary of the NSD (in Å):

basis	Δ_{ip}	δ_{ip}	B_{2g}	B_{1g}	$E_u(x)$	$E_u(y)$	A_{1g}	A_{2g}
min.	0.33	0.14	-0.31	0.09	0.01	0.03	0.06	0.01
ext.	0.38	0.11	-0.31	0.09	0.01	0.03	0.06	0.02
			-0.03	0.00	0.00	0.00	-0.17	0.07
total	0.50	0.00	-0.31	0.09	0.01	0.03	0.08	0.02
			-0.03	0.00	0.00	0.00	-0.17	0.07
			-0.02	-0.08	0.01	0.00	0.31	0.01
			0.01	0.00	-0.01	-0.01	-0.02	0.01
			-0.01	0.00	0.00	-0.01	-0.01	0.01
			-0.01	0.01	0.00	0.00	-0.01	
					0.00	0.00		
					0.00	0.00		
					0.00	0.00		
					0.00	0.00		
					0.00	0.00		
comp.	0.50	0.00	0.31	0.12	0.01	0.03	0.37	0.07

basis	Δ_{oop}	δ_{oop}	B_{2u}	B_{1u}	A_{2u}	$E_g(x)$	$E_g(y)$	A_{1u}
min.	1.93	0.27	-1.88	-0.33	0.23	-0.05	-0.04	-0.05
ext.	1.98	0.00	-1.87	-0.33	0.23	-0.05	-0.04	-0.05
			0.52	0.00	-0.01	0.00	0.01	0.02
total	1.98	0.00	-1.87	-0.33	0.23	-0.05	-0.04	-0.05
			0.52	0.00	-0.01	0.00	0.01	0.02
			0.04	-0.01	0.01	0.00	0.00	
						0.00	0.01	
						0.01	0.00	
comp.	1.98	0.00	1.94	0.33	0.23	0.05	0.04	0.06

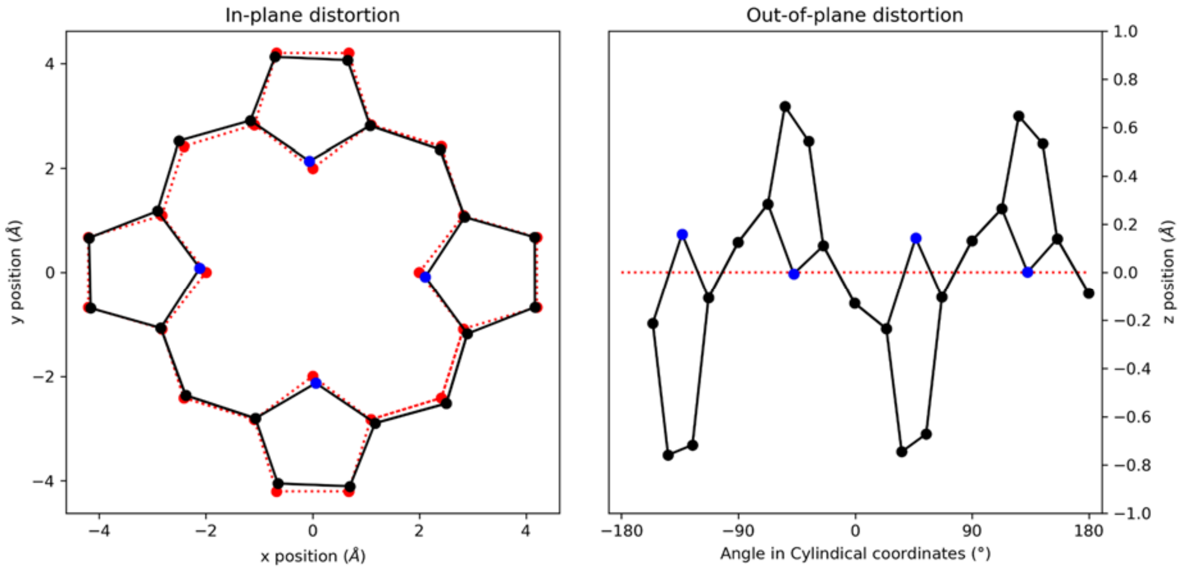

Figure S2.11; (a) out-of-plane and (b) in-plane skeletal plots of the porphyrin core.
Porphyrin is represented in black(C) and blue(N), with the reference structure (CuTPP) in red lines.

2.3.12 NSD result generated from $[H_4\mathbf{18}][CF_3CO_2]_2 \cdot 2CF_3CO_2H$ (5,15-bis(4-methylthiophenyl)porphyrindi-ium bis(trifluoroacetate) bis(trifluoroacetic acid) solvate) (HE)

Summary of the NSD (in Å):

basis	Δ_{ip}	δ_{ip}	B_{2g}	B_{1g}	$E_u(x)$	$E_u(y)$	A_{1g}	A_{2g}
min.	0.28	0.07	0.20	0.01	0.02	-0.01	0.20	0.01
ext.	0.31	0.06	0.20	0.01	0.02	-0.01	0.20	0.01
			0.04	0.00	0.02	0.01	-0.12	0.03
total	0.40	0.00	0.20	0.01	0.01	-0.01	0.21	0.01
			0.04	0.00	0.02	0.01	-0.12	0.03
			0.01	-0.01	0.01	0.00	0.23	0.01
			-0.01	0.00	-0.01	-0.02	0.00	0.00
			0.01	0.00	-0.03	-0.03	-0.01	0.00
			0.01	0.00	-0.01	0.00	0.00	
					0.00	0.00		
					0.00	0.00		
					0.00	0.00		
					0.00	0.00		
					0.00	0.00		
comp.	0.40	0.00	0.20	0.02	0.04	0.04	0.33	0.03

basis	Δ_{oop}	δ_{oop}	B_{2u}	B_{1u}	A_{2u}	$E_g(x)$	$E_g(y)$	A_{1u}
min.	1.36	0.26	-1.33	-0.18	0.04	-0.14	-0.12	0.01
ext.	1.44	0.00	-1.32	-0.18	0.04	-0.14	-0.12	0.01
			0.51	0.00	-0.01	0.01	-0.02	-0.01
total	1.44	0.00	-1.32	-0.18	0.04	-0.14	-0.12	0.01
			0.51	0.00	-0.01	0.01	-0.02	-0.01
			0.04	0.00	0.00	0.00	0.00	
						0.00	0.00	
						-0.01	-0.01	
comp.	1.44	0.00	1.42	0.18	0.04	0.15	0.12	0.02

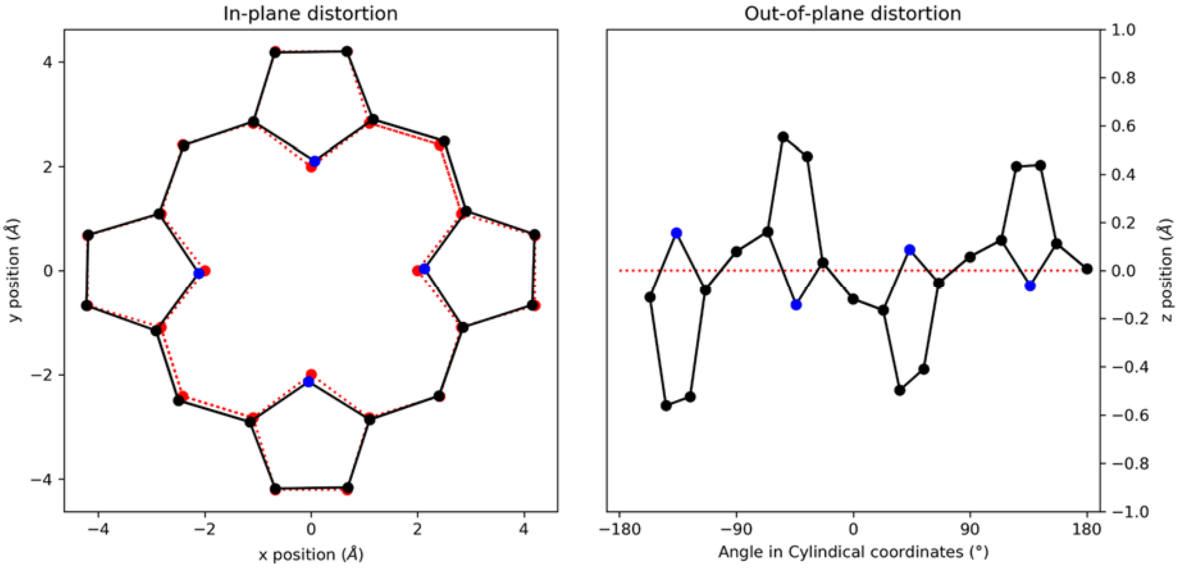

Figure S2.12; (a) out-of-plane and (b) in-plane skeletal plots of the porphyrin core. Porphyrin is represented in black(C) and blue(N), with the reference structure (CuTPP) in red lines.

2.3.13 NSD result generated from [H_4**18**][CF_3CO_2]$_2$·2CF_3CO_2H (5,15-bis(4-methylthiophenyl)porphyrindi-ium bis(trifluoroacetate) bis(trifluoroacetic acid) solvate) (MS)

Summary of the NSD (in Å):

basis	Δ_{ip}	δ_{ip}	B_{2g}	B_{1g}	$E_u(x)$	$E_u(y)$	A_{1g}	A_{2g}
min.	0.28	0.07	0.20	0.02	0.01	-0.01	0.20	0.01
ext.	0.31	0.06	0.20	0.02	0.01	-0.01	0.20	0.01
			0.04	0.00	0.01	0.01	-0.12	0.03
total	0.40	0.00	0.20	0.02	0.01	-0.01	0.21	0.01
			0.04	0.00	0.01	0.01	-0.12	0.03
			0.01	-0.01	0.01	0.00	0.23	0.01
			-0.01	0.00	-0.01	-0.02	0.00	0.00
			0.01	0.00	-0.03	-0.03	-0.01	0.00
			0.01	0.00	-0.01	0.00	0.00	
					0.00	0.00		
					0.00	0.00		
					0.00	0.00		
					0.00	0.00		
					0.00	0.00		
comp.	0.40	0.00	0.20	0.02	0.04	0.04	0.33	0.03

basis	Δ_{oop}	δ_{oop}	B_{2u}	B_{1u}	A_{2u}	$E_g(x)$	$E_g(y)$	A_{1u}
min.	1.36	0.26	-1.33	-0.17	0.04	-0.14	-0.12	0.01
ext.	1.44	0.00	-1.32	-0.17	0.04	-0.14	-0.12	0.01
			0.51	0.00	-0.01	0.01	-0.02	-0.01
total	1.44	0.00	-1.32	-0.17	0.04	-0.14	-0.12	0.01
			0.51	0.00	-0.01	0.01	-0.02	-0.01
			0.04	0.00	0.00	0.00	0.00	
						0.00	0.00	
						-0.01	-0.01	
comp.	1.44	0.00	1.42	0.17	0.04	0.15	0.12	0.02

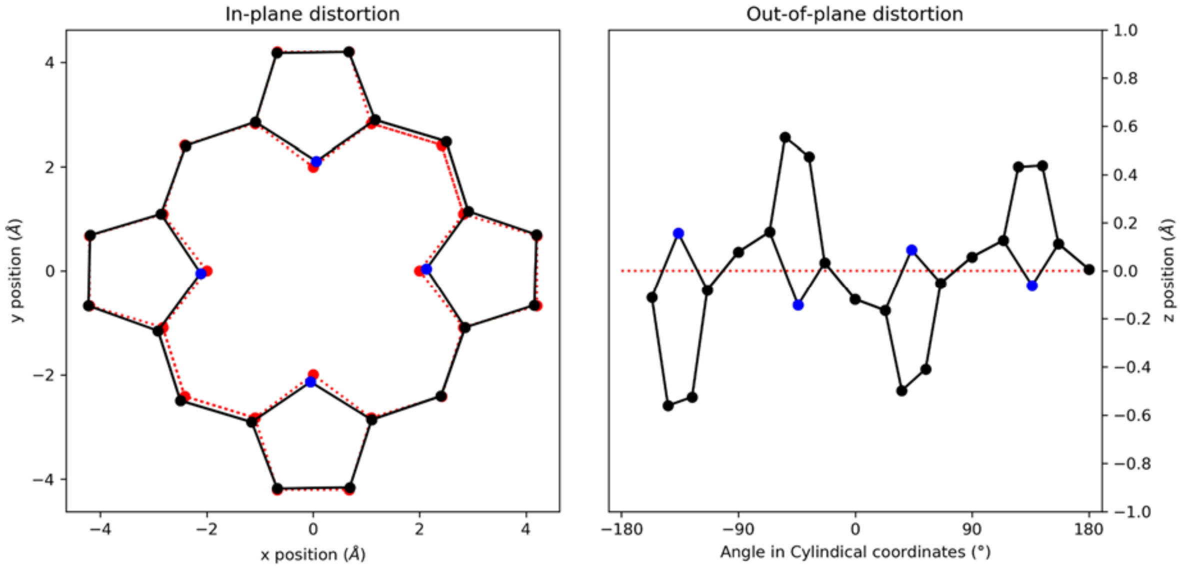

Figure S2.13; (a) out-of-plane and (b) in-plane skeletal plots of the porphyrin core.
Porphyrin is represented in black(C) and blue(N), with the reference structure (CuTPP) in red lines.

2.3.14 NSD result generated from H_2**16** (5,15-diphenyl-10-(thiophen-3-yl)porphyrin)

Summary of the NSD (in Å):

**basis**	Δ_{ip}	δ_{ip}	B_{2g}	B_{1g}	$E_u(x)$	$E_u(y)$	A_{1g}	A_{2g}
min.	0.30	0.01	-0.22	-0.06	0.00	0.00	0.20	0.00
ext.	0.30	0.00	-0.22	-0.07	0.00	0.00	0.20	0.00
			-0.04	-0.04	0.02	-0.01	-0.01	-0.01
total	0.31	0.00	-0.22	-0.07	0.00	0.00	0.20	0.00
			-0.04	-0.04	0.02	-0.01	-0.01	-0.01
			0.00	-0.03	0.00	0.00	0.05	0.00
			0.01	-0.01	-0.01	0.00	-0.01	0.00
			0.00	0.00	0.00	0.00	0.01	0.00
			0.00	-0.01	0.00	0.01	0.02	
					0.01	-0.01		
					0.00	0.01		
					0.00	0.01		
					0.00	0.00		
					0.00	0.00		
comp.	0.31	0.00	0.22	0.08	0.03	0.02	0.21	0.01

**basis**	Δ_{oop}	δ_{oop}	B_{2u}	B_{1u}	A_{2u}	$E_g(x)$	$E_g(y)$	A_{1u}
min.	0.43	0.01	-0.10	0.38	-0.11	-0.03	0.14	0.00
ext.	0.44	0.00	-0.09	0.38	-0.11	-0.03	0.14	0.00
			0.06	0.01	-0.02	-0.04	0.01	0.01
total	0.44	0.00	-0.09	0.38	-0.11	-0.03	0.14	0.00
			0.06	0.01	-0.02	-0.04	0.01	0.01
			-0.01	0.00	0.00	-0.01	-0.01	
						-0.01	-0.01	
						0.00	0.01	
comp.	0.44	0.00	0.11	0.38	0.11	0.05	0.14	0.01

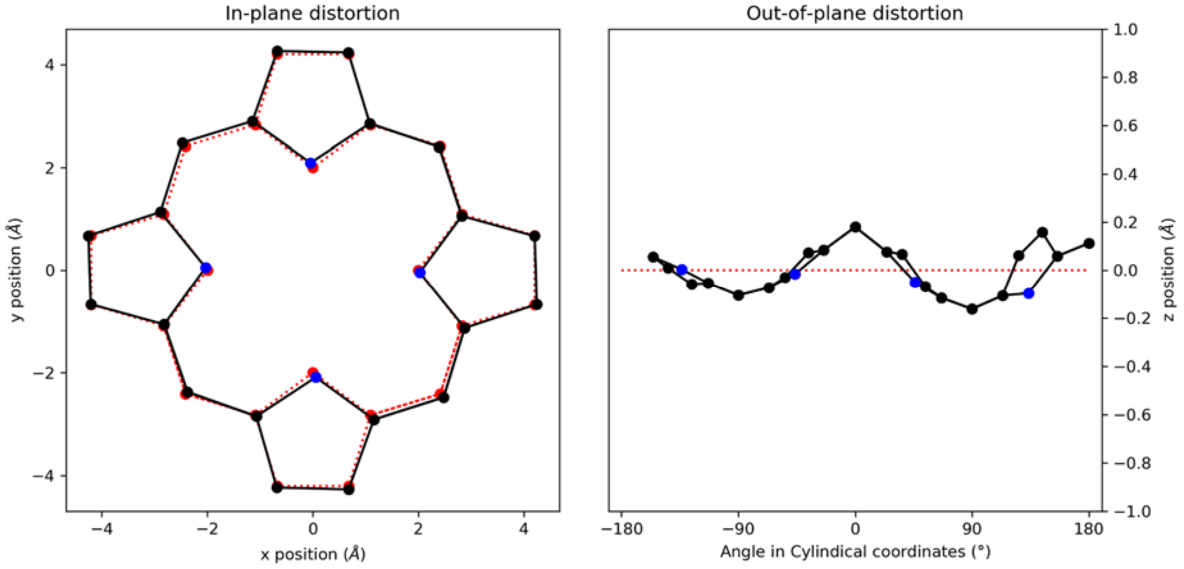

Figure S2.14; (a) out-of-plane and (b) in-plane skeletal plots of the porphyrin core. Porphyrin is represented in black(C) and blue(N), with the reference structure (CuTPP) in red lines.

2.3.15 NSD result generated from [H_4**20**][ClO_4]$_2$ (5-bromo-10,20-diphenylporphyrindi-ium diperchlorate)

Summary of the NSD (in Å):

basis	Δ_{ip}	δ_{ip}	B_{2g}	B_{1g}	$E_u(x)$	$E_u(y)$	A_{1g}	A_{2g}
min.	0.13	0.21	-0.11	0.02	0.01	-0.03	-0.04	0.02
ext.	0.26	0.16	-0.11	0.02	0.01	-0.03	-0.04	0.02
			-0.02	0.00	0.01	-0.01	-0.22	0.06
total	0.47	0.00	-0.11	0.02	0.01	-0.03	-0.02	0.02
			-0.02	0.00	0.01	-0.01	-0.22	0.06
			-0.03	0.00	0.00	-0.01	0.39	0.00
			0.01	0.00	-0.01	0.01	-0.02	0.01
			0.01	0.00	-0.02	0.04	-0.03	0.00
			-0.01	0.01	0.00	0.01	-0.02	
					0.00	0.01		
					0.00	0.00		
					0.00	0.00		
					0.01	0.00		
					0.00	-0.01		
comp.	0.47	0.00	0.12	0.03	0.04	0.05	0.45	0.07

basis	Δ_{oop}	δ_{oop}	B_{2u}	B_{1u}	A_{2u}	$E_g(x)$	$E_g(y)$	A_{1u}
min.	2.33	0.34	2.31	0.27	-0.02	-0.11	0.05	0.02
ext.	2.39	0.00	2.30	0.27	-0.02	-0.11	0.05	0.02
			-0.58	0.01	0.01	0.01	0.02	-0.01
total	2.39	0.00	2.30	0.27	-0.02	-0.11	0.05	0.02
			-0.59	0.01	0.01	0.01	0.02	-0.01
			-0.06	0.01	0.00	0.01	0.00	
						0.00	0.00	
						-0.01	0.00	
comp.	2.39	0.00	2.37	0.27	0.02	0.11	0.05	0.02

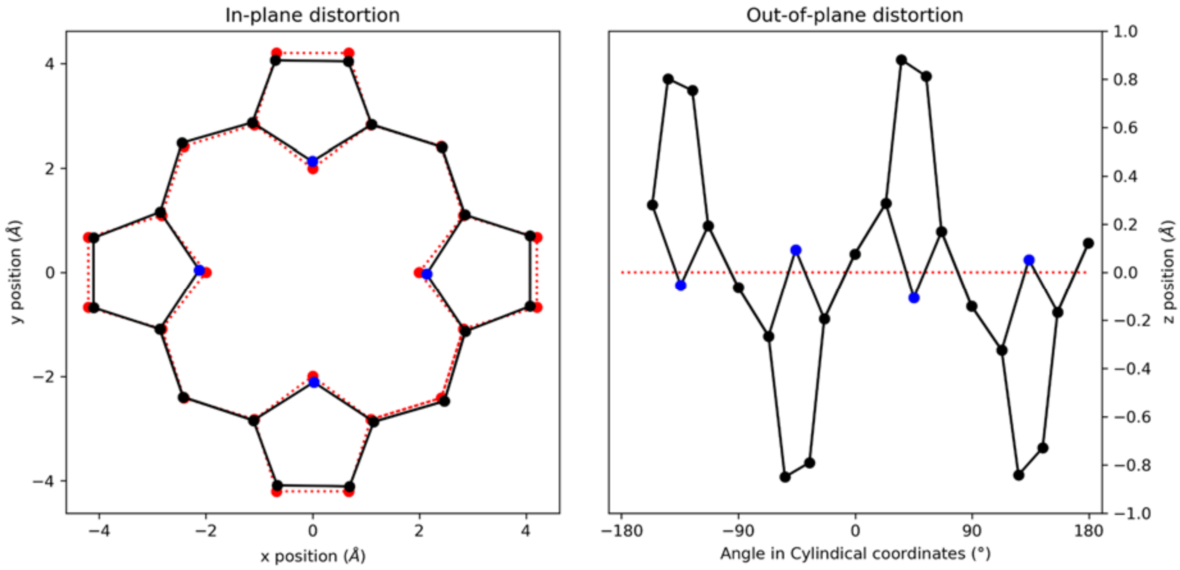

Figure S2.15; (a) out-of-plane and (b) in-plane skeletal plots of the porphyrin core.
Porphyrin is represented in black(C) and blue(N), with the reference structure (CuTPP) in red lines.

2.3.16 NSD result generated from file [H_4**21**][CF_3CO_2]$_2$·2CF_3CO_2H (5,15-dibromo-10,20-bis(4-tolyl)porphyrindi-ium bis(trifluoroacetate)bis(trifluoroacetic acid) solvate)

Summary of the NSD (in Å):

**basis**	**Δ_{ip}**	**δ_{ip}**	**B_{2g}**	**B_{1g}**	**$E_u(x)$**	**$E_u(y)$**	**A_{1g}**	**A_{2g}**
min.	0.16	0.11	-0.04	0.05	-0.01	0.01	0.14	-0.01
ext.	0.22	0.09	-0.04	0.05	-0.01	0.01	0.14	-0.01
			0.01	0.00	-0.01	0.00	-0.14	-0.06
total	0.38	0.00	-0.04	0.05	-0.01	0.01	0.16	-0.01
			0.01	0.00	-0.01	0.00	-0.14	-0.06
			-0.03	-0.03	0.00	0.00	0.29	-0.01
			0.02	0.00	0.00	-0.01	-0.02	-0.01
			0.00	0.00	0.02	0.00	-0.01	-0.01
			-0.01	0.00	0.00	0.01	-0.01	
					-0.01	0.00		
					0.00	0.00		
					0.00	0.00		
					0.00	0.00		
					0.00	0.00		
comp.	0.38	0.00	0.06	0.06	0.03	0.01	0.36	0.06

**basis**	**Δ_{oop}**	**δ_{oop}**	**B_{2u}**	**B_{1u}**	**A_{2u}**	**$E_g(x)$**	**$E_g(y)$**	**A_{1u}**
min.	1.73	0.32	1.69	-0.33	-0.07	0.01	-0.03	0.00
ext.	1.81	0.00	1.68	-0.33	-0.07	0.01	-0.03	0.00
			-0.56	-0.01	0.04	-0.02	-0.05	-0.01
total	1.81	0.00	1.68	-0.33	-0.07	0.01	-0.03	0.00
			-0.56	-0.01	0.04	-0.02	-0.05	-0.01
			-0.05	-0.01	-0.01	0.00	-0.02	
						0.00	-0.01	
						0.00	0.00	
comp.	1.81	0.00	1.78	0.33	0.08	0.03	0.06	0.01

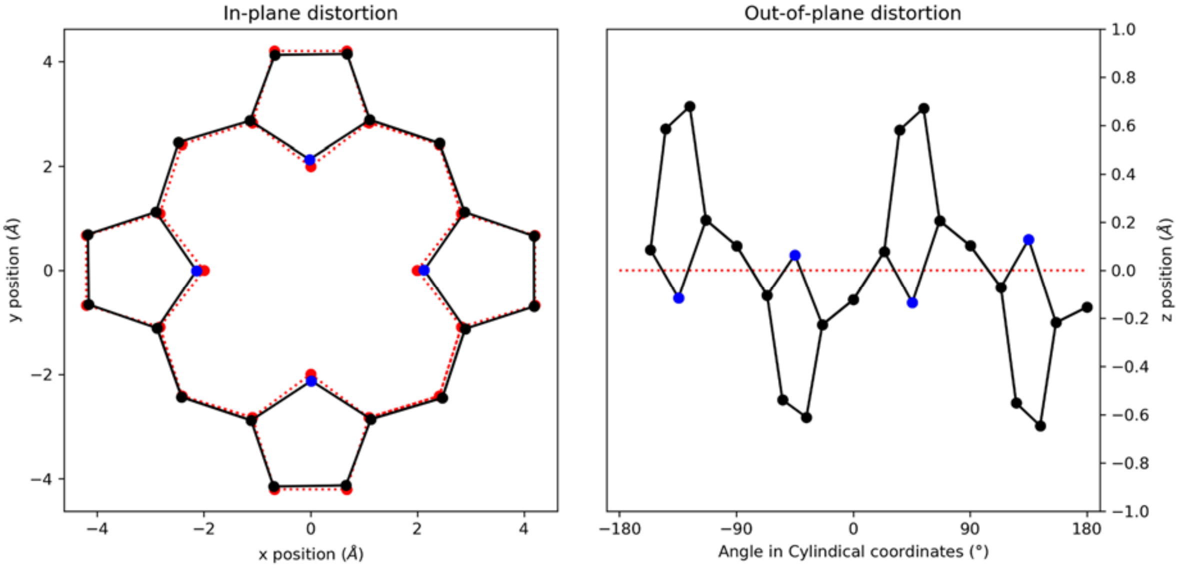

Figure S2.16; (a) out-of-plane and (b) in-plane skeletal plots of the porphyrin core.
Porphyrin is represented in black(C) and blue(N), with the reference structure (CuTPP) in red lines.

2.3.17 NSD result generated from $[H_8\mathbf{23}][ClO_4]_4{\cdot}2H_2O$ (5,5'-Bis(15-hexyl-10,20-bis(4-methoxyphenyl)porphyrindi-ium) tetrakis(perchlorate) diaqua solvate)

Summary of the NSD (in Å):

basis	Δ_{ip}	δ_{ip}	B_{2g}	B_{1g}	$E_u(x)$	$E_u(y)$	A_{1g}	A_{2g}
min.	0.10	0.24	-0.01	0.04	0.00	0.05	-0.06	0.05
ext.	0.30	0.16	-0.01	0.04	-0.01	0.06	-0.06	0.05
			0.00	0.01	-0.06	0.06	-0.24	0.11
total	0.49	0.00	-0.01	0.04	-0.01	0.05	-0.04	0.05
			0.00	0.01	-0.06	0.05	-0.24	0.11
			0.00	0.05	0.00	0.03	0.38	0.01
			-0.01	0.00	0.03	-0.03	-0.02	0.01
			0.00	0.00	0.02	-0.07	-0.01	0.01
			0.00	0.00	0.00	-0.01	-0.03	
					0.01	0.00		
					0.00	0.00		
					-0.01	0.01		
					0.00	0.01		
					-0.01	0.01		
comp.	0.49	0.00	0.01	0.07	0.08	0.11	0.45	0.12

basis	Δ_{oop}	δ_{oop}	B_{2u}	B_{1u}	A_{2u}	$E_g(x)$	$E_g(y)$	A_{1u}
min.	2.34	0.37	2.28	0.47	0.11	0.23	-0.12	0.01
ext.	2.41	0.01	2.27	0.47	0.11	0.23	-0.12	0.01
			-0.60	0.01	0.01	0.01	0.06	0.00
total	2.41	0.00	2.27	0.47	0.11	0.23	-0.12	0.01
			-0.61	0.01	0.01	0.01	0.06	0.00
			-0.05	0.01	0.00	-0.03	0.03	
						0.00	0.00	
						0.02	-0.02	
comp.	2.41	0.00	2.35	0.47	0.11	0.23	0.14	0.01

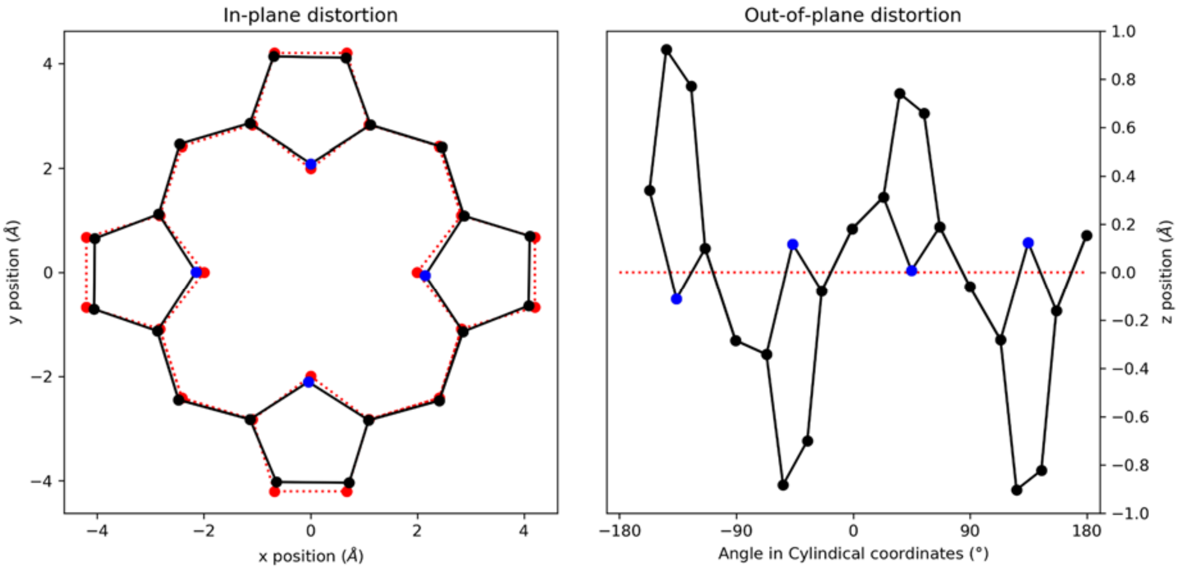

Figure S2.17; (a) out-of-plane and (b) in-plane skeletal plots of the porphyrin core.
Porphyrin is represented in black(C) and blue(N), with the reference structure (CuTPP) in red lines.

2.3.18 NSD result generated from [H_4**22**][$MeSO_4$]$_2$·¼H_2O (5,15-bis(4-ethynylphenyl)-10,20-diphenylporphyrindi-ium bis(methylsulfate) hemiaqua solvate)

Summary of the NSD (in Å):

basis	Δ_{ip}	δ_{ip}	B_{2g}	B_{1g}	$E_u(x)$	$E_u(y)$	A_{1g}	A_{2g}
min.	0.18	0.34	0.00	0.01	0.04	-0.01	-0.17	0.00
ext.	0.36	0.24	0.00	0.01	0.04	-0.01	-0.17	0.00
			0.00	-0.03	0.04	-0.01	-0.31	0.01
total	0.60	0.00	0.00	0.00	0.04	-0.01	-0.15	0.00
			0.00	-0.03	0.04	-0.01	-0.31	0.01
			0.00	-0.09	0.01	0.00	0.47	0.00
			-0.01	0.00	-0.01	0.01	-0.03	0.00
			0.00	-0.01	-0.01	0.00	-0.02	0.00
			0.00	0.01	0.00	0.00	-0.05	
					0.00	0.00		
					0.00	0.00		
					0.01	0.00		
					0.00	0.00		
					0.00	0.00		
comp.	0.60	0.00	0.01	0.10	0.06	0.02	0.59	0.01

basis	Δ_{oop}	δ_{oop}	B_{2u}	B_{1u}	A_{2u}	$E_g(x)$	$E_g(y)$	A_{1u}
min.	2.64	0.42	-2.63	-0.03	0.17	0.02	-0.05	-0.01
ext.	2.70	0.00	-2.62	-0.03	0.17	0.02	-0.05	-0.01
			0.64	0.00	0.03	-0.01	0.04	0.00
total	2.70	0.00	-2.62	-0.03	0.17	0.02	-0.05	-0.01
			0.65	0.00	0.03	-0.01	0.04	0.00
			0.06	0.00	0.00	0.00	0.02	
						0.00	0.00	
						0.00	-0.01	
comp.	2.70	0.00	2.70	0.03	0.18	0.02	0.06	0.01

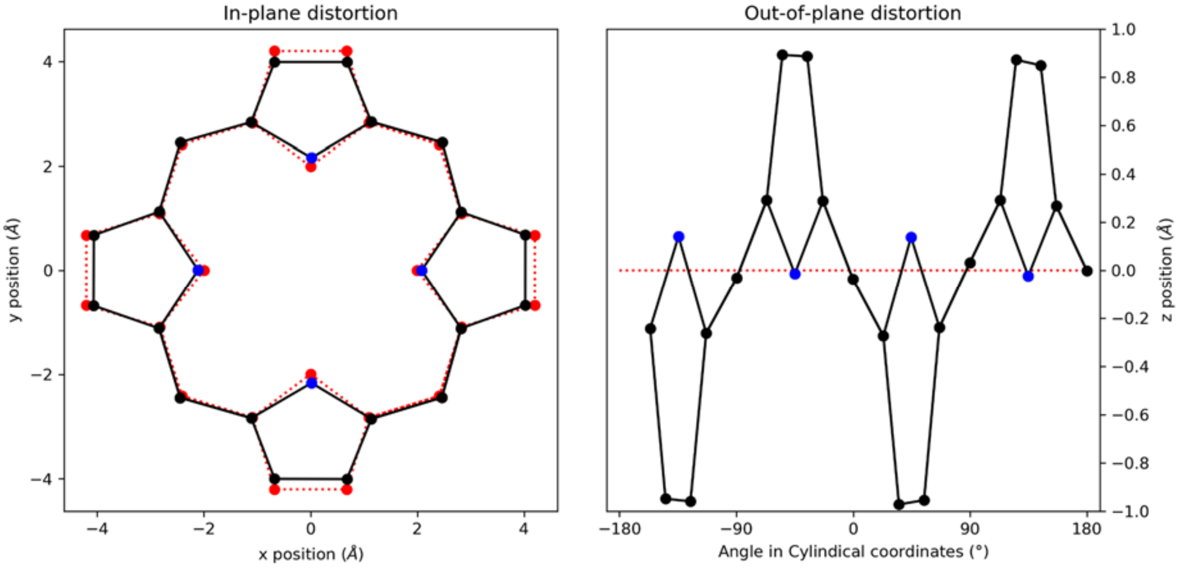

Figure S2.18; (a) out-of-plane and (b) in-plane skeletal plots of the porphyrin core. Porphyrin is represented in black(C) and blue(N), with the reference structure (CuTPP) in red lines.

2.3.19 NSD result generated from $[H_4\mathbf{18}][CF_3CO_2]_2{\cdot}2CF_3CO_2H$ (5,15-bis(4-bromophenyl)porphyrindi-ium bis(trifluoroacetate) bis(trifluoroacetic acid) solvate)

Summary of the NSD (in Å):

**basis**	**Δ_{ip}**	**δ_{ip}**	**B_{2g}**	**B_{1g}**	**$E_u(x)$**	**$E_u(y)$**	**A_{1g}**	**A_{2g}**
min.	0.32	0.07	-0.18	-0.05	0.00	-0.02	0.26	0.00
ext.	0.35	0.05	-0.18	-0.05	0.00	-0.02	0.26	0.00
			-0.03	-0.01	0.02	-0.03	-0.12	0.02
total	0.42	0.00	-0.18	-0.05	0.00	-0.02	0.27	0.00
			-0.03	-0.01	0.02	-0.03	-0.12	0.02
			-0.01	0.01	0.00	0.00	0.22	0.00
			0.01	0.00	-0.02	0.02	0.00	0.00
			-0.01	0.00	-0.03	0.03	-0.01	0.01
			-0.01	0.00	0.00	0.00	0.00	
					-0.01	0.00		
					0.00	0.00		
					0.00	0.00		
					0.00	0.00		
					0.01	0.00		
comp.	0.42	0.00	0.19	0.05	0.04	0.05	0.37	0.02

**basis**	**Δ_{oop}**	**δ_{oop}**	**B_{2u}**	**B_{1u}**	**A_{2u}**	**$E_g(x)$**	**$E_g(y)$**	**A_{1u}**
min.	1.28	0.25	-1.25	-0.12	-0.06	0.16	-0.14	-0.02
ext.	1.36	0.00	-1.24	-0.12	-0.06	0.16	-0.14	-0.02
			0.49	0.00	0.02	0.02	0.03	0.02
total	1.36	0.00	-1.24	-0.12	-0.06	0.16	-0.14	-0.02
			0.49	0.00	0.02	0.02	0.03	0.02
			0.04	0.00	-0.01	-0.01	0.00	
						0.01	0.00	
						0.02	-0.02	
comp.	1.36	0.00	1.34	0.12	0.06	0.16	0.14	0.02

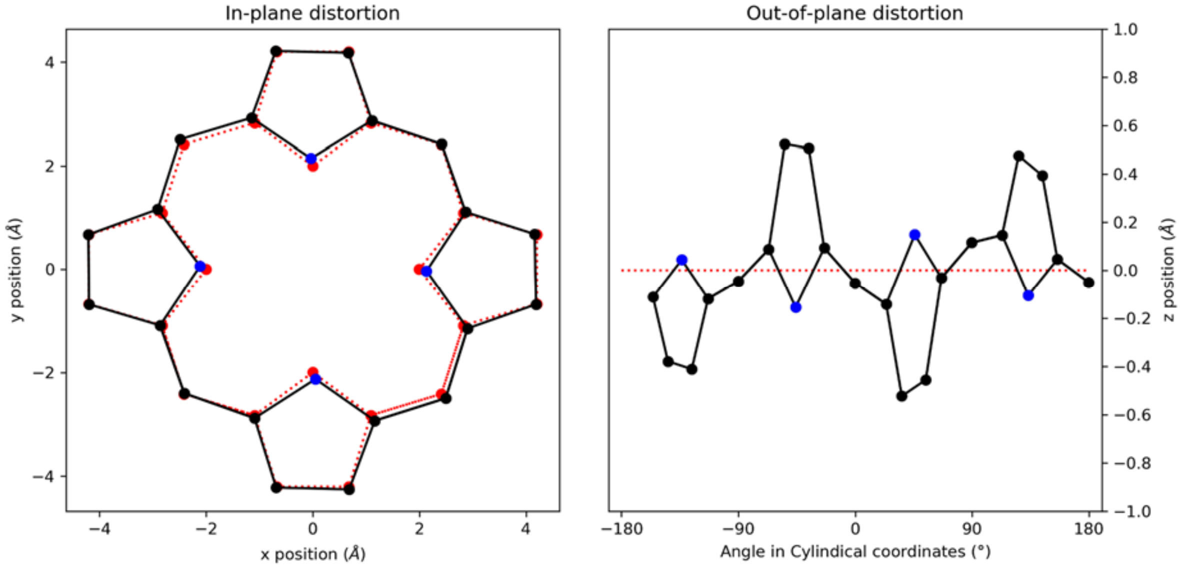

Figure S2.19; (a) out-of-plane and (b) in-plane skeletal plots of the porphyrin core.
Porphyrin is represented in black(C) and blue(N), with the reference structure (CuTPP) in red lines.

2.3.20 NSD result generated from [H_8**24**][CF_3CO_2]$_4$·14H_2O (5,5'-bis(10,15,20-triphenylporphyrindi-ium) tetrakis(trifluoroacetate) aqua solvate)

Summary of the NSD (in Å):

**basis**	Δ_{ip}	δ_{ip}	B_{2g}	B_{1g}	$E_u(x)$	$E_u(y)$	A_{1g}	A_{2g}
min.	0.20	0.09	0.07	0.03	0.00	-0.01	0.18	0.00
ext.	0.25	0.07	0.07	0.03	0.00	-0.01	0.18	0.00
			0.00	-0.01	0.01	0.00	-0.15	0.00
total	0.37	0.00	0.07	0.03	0.00	-0.01	0.20	0.00
			0.00	-0.01	0.01	0.00	-0.15	0.00
			0.00	0.00	0.02	0.00	0.26	0.00
			0.00	-0.01	0.01	0.00	-0.03	0.01
			0.01	0.01	0.03	0.01	0.00	0.00
			-0.01	0.01	0.01	0.01	-0.01	
					0.00	-0.01		
					0.00	0.00		
					0.01	0.01		
					0.01	0.01		
					0.00	0.00		
comp.	0.37	0.00	0.07	0.03	0.04	0.02	0.36	0.01

**basis**	Δ_{oop}	δ_{oop}	B_{2u}	B_{1u}	A_{2u}	$E_g(x)$	$E_g(y)$	A_{1u}
min.	1.55	0.31	-1.54	0.01	-0.01	0.05	0.03	0.01
ext.	1.63	0.00	-1.53	0.01	-0.01	0.05	0.03	0.01
			0.55	-0.01	-0.03	0.04	0.07	-0.01
total	1.64	0.00	-1.53	0.01	-0.01	0.05	0.03	0.01
			0.55	-0.01	-0.03	0.04	0.07	-0.01
			0.04	0.00	-0.01	-0.01	0.03	
						0.00	0.02	
						0.01	0.00	
comp.	1.64	0.00	1.63	0.02	0.04	0.07	0.09	0.02

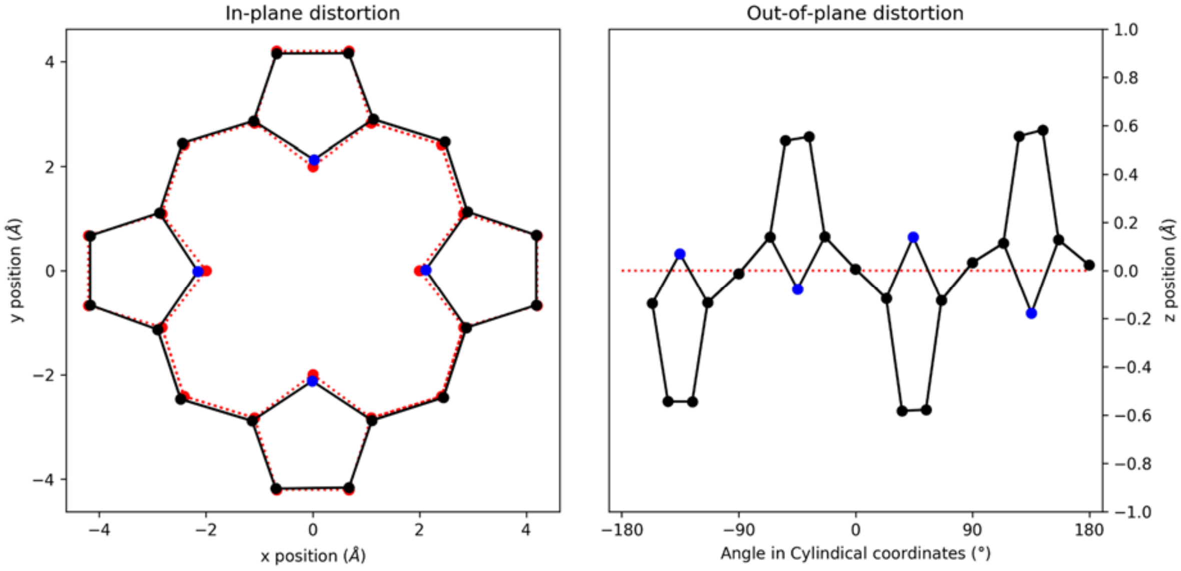

Figure S2.20; (a) out-of-plane and (b) in-plane skeletal plots of the porphyrin core. Porphyrin is represented in black(C) and blue(N), with the reference structure (CuTPP) in red lines.

2.3.21 NSD result generated from CCDC entry ODEFOS (H_2**11** (monoclinic), 5,15-diphenylporphyrin)

Summary of the NSD (in Å):

**basis**	Δ_{ip}	δ_{ip}	B_{2g}	B_{1g}	$E_u(x)$	$E_u(y)$	A_{1g}	A_{2g}
min.	0.45	0.00	-0.42	-0.02	-0.01	0.00	0.17	-0.01
ext.	0.46	0.00	-0.42	-0.02	-0.01	0.00	0.17	-0.01
			-0.09	-0.06	0.00	0.01	-0.01	-0.01
total	0.47	0.00	-0.42	-0.03	-0.01	0.00	0.17	-0.01
			-0.09	-0.06	0.00	0.01	-0.01	-0.01
			-0.01	-0.04	-0.01	0.00	0.05	0.00
			0.00	0.00	0.00	0.00	-0.01	0.00
			-0.01	0.01	0.00	0.00	0.01	0.00
			-0.01	-0.01	0.00	0.00	0.01	
					0.00	0.00		
					0.00	0.00		
					0.00	0.00		
					0.00	0.00		
					0.00	0.00		
comp.	0.47	0.00	0.43	0.08	0.01	0.01	0.18	0.01
**basis**	Δ_{oop}	δ_{oop}	B_{2u}	B_{1u}	A_{2u}	$E_g(x)$	$E_g(y)$	A_{1u}
min.	0.43	0.00	0.13	-0.40	0.05	0.07	-0.03	0.00
ext.	0.44	0.00	0.13	-0.40	0.05	0.07	-0.03	0.00
			-0.07	0.00	-0.01	-0.06	0.08	-0.01
total	0.45	0.00	0.13	-0.40	0.05	0.07	-0.03	0.00
			-0.07	0.00	-0.01	-0.06	0.08	-0.01
			0.00	0.00	0.00	-0.02	0.02	
						-0.01	0.00	
						0.01	-0.01	
comp.	0.45	0.00	0.15	0.40	0.05	0.09	0.09	0.01

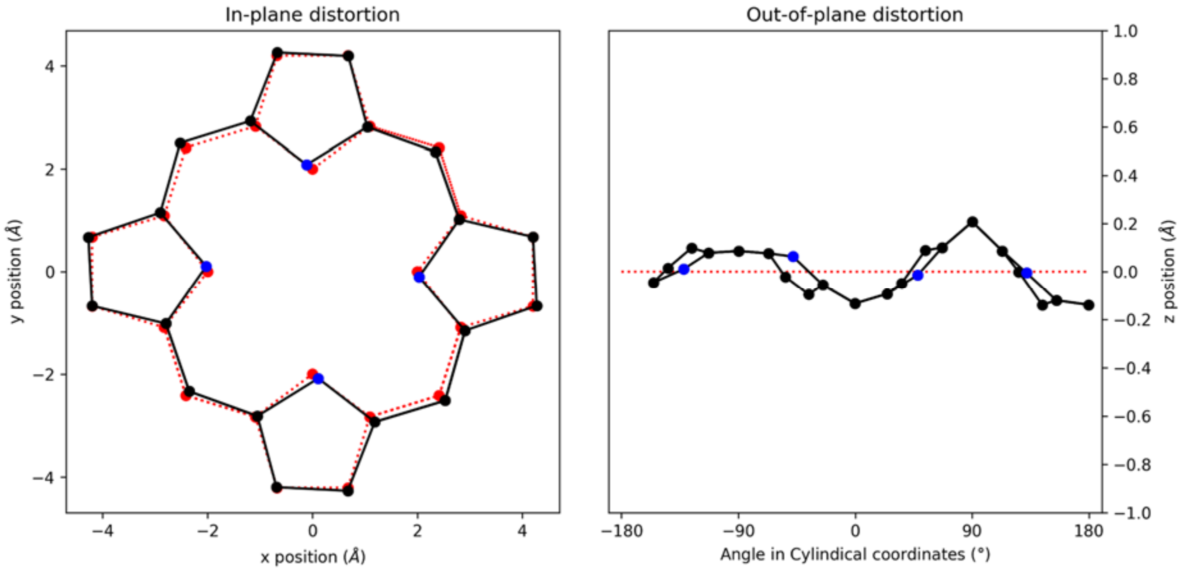

Figure S2.21; (a) out-of-plane and (b) in-plane skeletal plots of the porphyrin core. Porphyrin is represented in black(C) and blue(N), with the reference structure (CuTPP) in red lines.

2.3.22 NSD result generated from CCDC entry MOSQEQ (H_2**14** 5,10,15-triphenylporphyrin)

Summary of the NSD (in Å):

**basis**	**Δ_{ip}**	**δ_{ip}**	**B_{2g}**	**B_{1g}**	**$E_u(x)$**	**$E_u(y)$**	**A_{1g}**	**A_{2g}**
min.	0.27	0.00	-0.21	-0.03	-0.02	0.03	0.16	0.01
ext.	0.28	0.00	-0.21	-0.03	-0.02	0.03	0.16	0.02
			-0.05	-0.07	0.04	-0.02	0.00	0.01
total	0.29	0.00	-0.21	-0.03	-0.02	0.03	0.16	0.02
			-0.05	-0.07	0.04	-0.02	0.00	0.01
			-0.01	-0.05	0.01	0.00	0.04	0.00
			0.00	0.00	-0.01	0.00	-0.01	0.00
			0.00	0.01	0.00	-0.01	0.01	0.01
			-0.01	-0.01	0.01	0.00	0.02	
					0.00	0.00		
					-0.01	-0.01		
					0.00	0.00		
					0.00	0.00		
					0.00	0.00		
comp.	0.29	0.00	0.21	0.09	0.04	0.03	0.17	0.02

**basis**	**Δ_{oop}**	**δ_{oop}**	**B_{2u}**	**B_{1u}**	**A_{2u}**	**$E_g(x)$**	**$E_g(y)$**	**A_{1u}**
min.	0.70	0.01	0.22	0.64	0.12	-0.14	0.03	0.01
ext.	0.72	0.00	0.22	0.64	0.12	-0.14	0.03	0.01
			-0.04	0.01	-0.03	-0.13	-0.05	0.00
total	0.72	0.00	0.22	0.64	0.12	-0.14	0.03	0.01
			-0.04	0.01	-0.03	-0.13	-0.05	0.00
			0.00	0.01	0.00	-0.01	-0.01	
						0.01	-0.01	
						0.00	0.01	
comp.	0.72	0.00	0.23	0.64	0.12	0.19	0.06	0.01

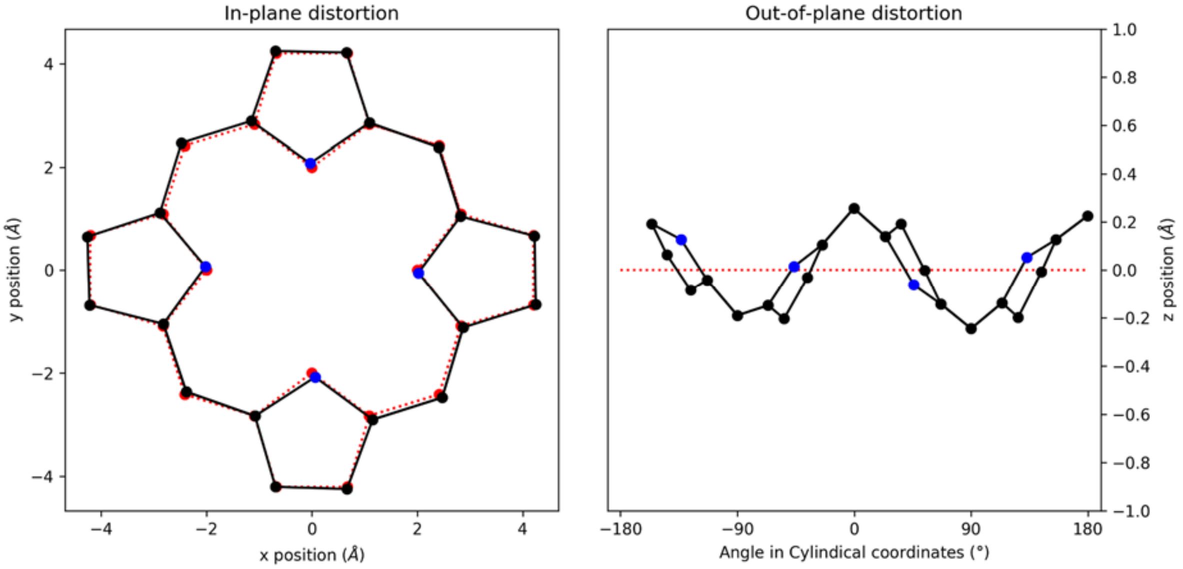

Figure S2.22; (a) out-of-plane and (b) in-plane skeletal plots of the porphyrin core.
Porphyrin is represented in black(C) and blue(N), with the reference structure (CuTPP) in red lines.

S3. Supplemental Images and tables

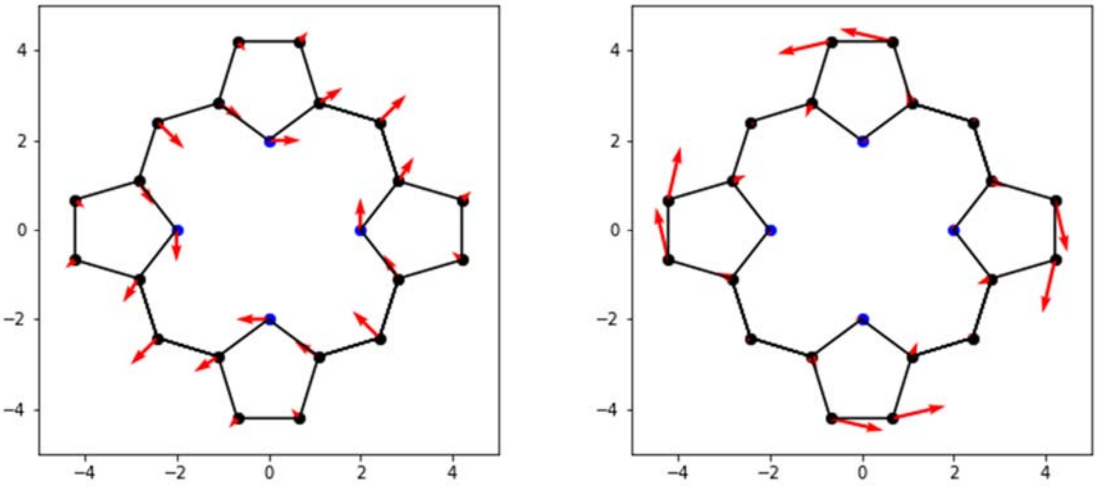

Figure S3.1; The NSD distortion modes (a) B_{2g}(1) and (b) B_{2g}(2) from the reference structure (CuTPP); arrows represent a 1 Å deviation along each distortion mode.

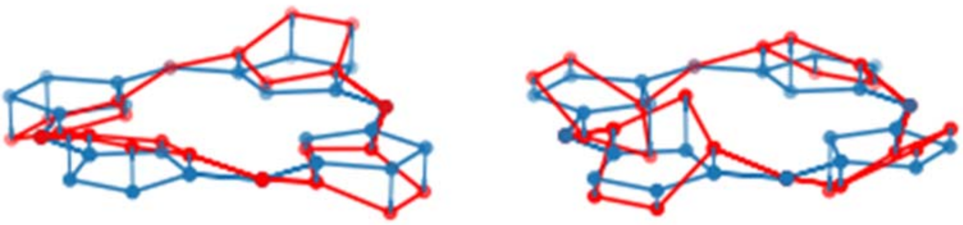

Figure S3.2; The NSD-identified distortion modes (a) B_{2u}*(1)* and (b) B_{2u}*(2)*; The reference CuTPP structure is shown in blue, and the red skeleton represents a 4 Å deviation along each distortion mode.

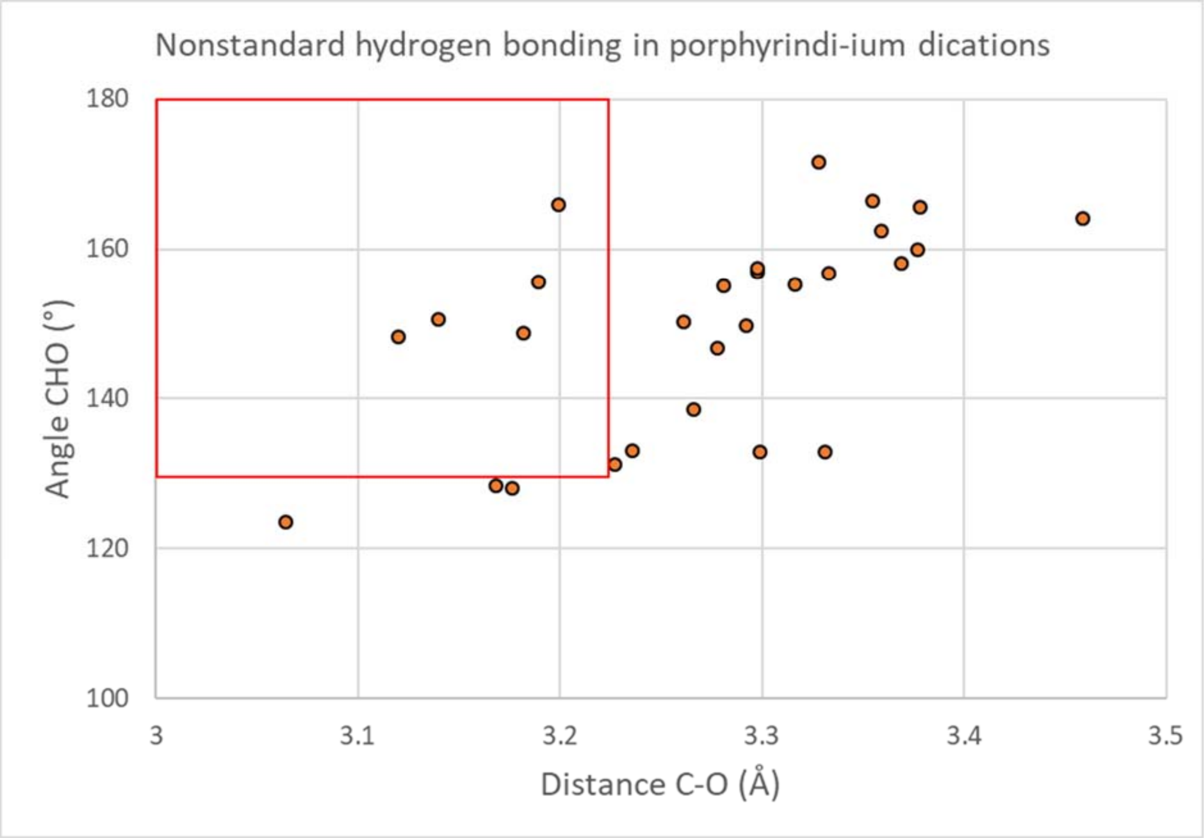

Figure S3.3; a plot of the critical parameters for non-standard hydrogen bonding in crystal structures of porphyrindi-ium dications. The red box indicates those relationships which fall into the definition of strong H-bonds (i.e. less than the sum of the van der Waals radii of the donor and acceptor atoms and approximately linear) as outlined by Steiner[32]

Table S3.4; Non-standard C-H···O close contacts in previously reported porphyrin molecules from the CCDC CSD

CCDC identifier	Link to paper (doi)	Compound Name	D-A distance	D-H-A angle
Meso- donors				
MANHOZ	http://dx.doi.org/10.1016/j.tet.2005.01.128	5-(3,5-Dimethoxyphenyl)porphyrin bis(trifluoroacetate)	3.215	167.106
NAWYOZ	http://dx.doi.org/10.1002/anie.199724971	5,11,17,23-tetrakis((1,2,6,7-Tetraethylporphyrin-15-yl)-nickel)-25,26,27,28-tetrahydroxycalix(4)arene methanol solvate	3.106	152.632
Beta- donors				
AYULOX	http://dx.doi.org/10.1039/C6TC03957J	1-methyl-4-(10,15,20-tris(1-methylpyridinium-4-yl)porphyrin-5-yl)pyridinium 4-(10,15,20-tris(4-sulfonatophenyl)porphyrin-5-yl)benzenesulfonate hydrate	3.17	161.212
ECIKIL	http://dx.doi.org/10.1002/ejoc.200500685	alpha-5,15-beta-10,20-bis(2,2'-(3,3'-(2,2-bis(Ethoxycarbonyl)propane-1,3-diyl)dibenzoylamino)diphenyl)porphyrin	3.181	144.756
EMOREF	http://dx.doi.org/10.1016/j.ica.2016.05.038	2,2',2'',2'''-(porphyrin-5,10,15,20-tetrayltetrakis((2,3,5,6-tetrafluoro-4,1-phenylene)oxy))tetraethanol dimethyl sulfoxide solvate	3.217	143.15
ERAKOY	http://dx.doi.org/10.1021/ic902309f	(5,10,15,20-tetrakis(2-(Pivaloylamino)phenyl)porphyrinato)-(2-methylimidazole-N$3!)-(2-methylimidazolato)-cobalt(iii) benzene solvate	3.184	141.78
HABSEL	http://dx.doi.org/10.1002/cssc.201402242	N-phenyl-6-(10,15,20-tris(3,4,5-trimethoxyphenyl)porphyrin-5-yl)dibenzo[b,d]furan-4-carboxamide acetonitrile solvate	3.194	161.396
HONVOX	http://dx.doi.org/10.1246/cl.140392	4,4',4'',4'''-Porphyrin-5,10,15,20-tetrayltetrakis(1-(3-(acetylsulfanyl)propyl)pyridinium) 4,4',4'',4'''-porphyrin-5,10,15,20-tetrayltetrabenzenesulfonate acetonitrile solvate tetrahydrate	3.167	145.788
KIBMEN	SENGE, M.O.; Z Naturforsch B: Chem Sci, 2000, 55, 336	22,24-Dihydro-5,10,15,20-tetraisopropylporphyrindi-ium bis(trifluoroacetate) trifluoroacetic acid solvate chloroform solvate	3.193	150.339
LIHMAR	http://dx.doi.org/10.1039/b616884a	20-(2,7-Di-t-butyl-4-carboxy-9,9-dimethylxanthen-5-yl)-5,10,15-tris(perfluorophenyl)porphyrin	3.153	165.789
NALZAD	http://dx.doi.org/10.1021/ic200243y	(mu!2$-4'-Methyl-N-(4-(10,15,20-triphenylporphyrin-5-yl)phenyl)-2,2'-bipyridine-4-carboxamide)-tricarbonyl-isothiocyanato-palladium(ii)-rhenium(i) benzene solvate	3.186	149.176
PEMQEF	http://dx.doi.org/10.1021/ja307349d	(mu!2$-5,15:10,20-bis(2,2'-((2-carboxypropane-1,3-diyl)bis(benzene-3,1-diylcarbonylimino))diphenyl)porphyrinato)-bis(acetato)-diaqua-di-bismuth(iii) chloroform unknown solvate	3.139	146.089
QOLZUM	http://dx.doi.org/10.1021/ja003184q	(5,10,15,20-tetrakis(Pentafluorophenyl)porphyrinato)-dioxo-osmium methanol solvate	3.163	147.185
VATYAQ	http://dx.doi.org/10.1021/ja982052i	(alpha,alpha,alpha,alpha)-5,10,15,20-tetrakis(2-(4-Chlorophenylurea)phenyl)porphyrin tetra-n-butylammonium bromide dimethylsulfoxide solvate	3.169	140.482
WEYYUW	http://dx.doi.org/10.1039/C3CE27064E	Dimethanol-(5,10,15,20-tetraphenylporphyrinato)-manganese bis(mu-2-cyano)-dicyano-(5,10,15,20-tetraphenylporphyrinato)-bis(1,2-bis(pyridine-2-carboxamido)-4-methylbenzenate)-di-cobalt-manganese methanol solvate dihydrate	3.203	142.485

Table S3.5; Non-standard C-H···X (X = O, S, F) close contacts from the porphyrin core in porphyrindi-ium molecules reported in this paper.

Structure	Donor	H	Acceptor	Distance C···O (Å)	Angle C-H···O (°)
$[H_4$**11**$][CF_3CO_2]_2$	C10	H10	O2A	3.298	156.96
	C18	H18	O2B	3.064	123.5
	C20	H20	O2B	3.182	148.8
	C2	H2	O2B	3.299	132.94
$[H_4$**11**$][CF_3CO_2]_2{\cdot}2CF_3CO_2H$	C308	H308	O4A	3.333	156.84
	C307	H307	O4S	3.176	128.12
	C302	H302	O2A	3.236	133.06
	C318	H318	O1B	3.14	150.65
	C12	H12	O8A	3.266	138.54
	C2	H2	O3S	3.369	158.14
	C17	H17	O2B	3.281	155.1
	C18	H18	O6A	3.278	146.7
$[H_4$**13**$][CF_3CO_2]_2$	C3	H3A	O1A	3.189	155.6
	C10	H10A	O1A	3.292	149.8
$[H_4$**14**$][CF_3CO_2]_2$	C20	H20	O2A	3.199	165.9
	C17	H17	O3A	3.316	155.29
$[H_4$**17**$][CF_3CO_2]_2{\cdot}2CF_3CO_2H$	C2	H2	O1	3.298	157.38
	C8	H8	O3S	3.378	165.65
$[H_4$**18**$][ClO_4]_2$	C10	H10A	O6	3.261	150.2
	C12	H12A	O6	3.331	132.9
	C20	H20A	O3A	3.12	148.3
	C2	H2A	O3A	3.168	128.35
$[H_4$**18**$][CF_3CO_2]_2{\cdot}2CF_3CO_2H$	C8	H8A	O1S	3.359	162.38
	C18	H18A	O2A	3.459	164.12
$[H_4$**19**$][CF_3CO_2]_2{\cdot}2CF_3CO_2H$	C8	H8A	O6S	3.328	171.6
	C12	H12A	O8S	3.377	159.94
	C18	H18A	S1	3.681	159.25
$[H_4$**21**$][CF_3CO_2]_2{\cdot}2CF_3CO_2H$	C3	H3	O6S	3.227	131.17
	C12	H12	O5S	3.355	166.45
	C17	H17	F7S	3.51	157.86
